# Supplementary material for: L-DOPA and Droxidopa: From Force Field Development to Molecular Docking into Human β2-Adrenergic Receptor
Source: Life (Basel). 2022 Sep 6;12(9):1393. doi: 10.3390/life12091393 (PMC9501711; doi:10.3390/life12091393)
Supplement: Supplementary file 1 [file life-12-01393-s001.zip › life-1817394-supplementary.pdf]

**Supporting Information**

# L-DOPA and Droxidopa: from force development to molecular docking into Human $\beta_2$-Adrenergic Receptor

Andrea Catte,[1§] Akash Deep Biswas,[1§] Giordano Mancini,[1] and Vincenzo Barone[1]

[1]Scuola Normale Superiore, Piazza dei Cavalieri 7, I-56126 Pisa, Italy
[§]Contributed equally to this work

E-mail: vincenzo.barone@sns.it; andrea.catte@sns.it

**Table of Contents**

**Calculation of torsional profiles with subtracted Coulomb and Lennard-Jones contributions**
**Figure S1.** Geometry optimized chemical structures of protonated adrenaline, zwitterionic L-DOPA and Droxidopa.
**Figure S2.** Torsional profiles derived from QM calculations and JOYCE for protonated adrenaline.
**Table S1.** Partial atomic charges and OPLS AA force field Lennard-Jones parameters of adrenaline.
**Figure S3.** OPLS AA force field parameters of adrenaline: bond lengths and force constants.
**Figure S4.** OPLS AA force field parameters of adrenaline: bond angles and force constants.
**Figure S5.** OPLS AA force field parameters of adrenaline: dihedral angles, force constants and multiplicity.
**Table S2.** Partial atomic charges and OPLS AA force field Lennard-Jones parameters of L-DOPA.
**Figure S6.** OPLS AA force field parameters of L-DOPA: bond lengths and force constants.
**Figure S7.** OPLS AA force field parameters of L-DOPA: bond angles and force constants.
**Figure S8.** OPLS AA force field parameters of L-DOPA: dihedral angles, force constants and multiplicity.
**Table S3.** OPLS AA force field Lennard-Jones parameters of Droxidopa.
**Figure S9.** OPLS AA force field parameters of Droxidopa: bond lengths and force constants.
**Figure S10.** OPLS AA force field parameters of Droxidopa: bond angles and force constants.
**Figure S11.** OPLS AA force field parameters of Droxidopa: dihedral angles, force constants and multiplicity.
**Figure S12.** Comparison of Dihedrals distributions in vacuum and water to torsional profiles obtained by QM calculations and JOYCE for protonated adrenaline.
**Table S4.** Coulomb and Lennard-Jones energies of adrenaline, L-DOPA and Droxidopa.
**Figure S13.** Comparison of protonated adrenaline dihedral angles torsional energy profiles from QM, JOYCE, QM after the subtraction of electrostatic and LJ energy contributions and fittings calculations.
**Figure S14.** Comparison of zwitterionic L-DOPA dihedral angles torsional energy profiles from QM, JOYCE, QM after the subtraction of electrostatic and Lennard-Jones (LJ) energy contributions and fittings calculations.

**Figure S15.** Comparison of zwitterionic Droxidopa dihedral angles torsional energy profiles from QM, JOYCE, QM after the subtraction of electrostatic and LJ energy contributions and fittings calculations.
**Figure S16.** Comparison of protonated adrenaline QM torsional energy profiles with Helmholtz free energy variations obtained from MD simulations performed using JOYCE and OPLS FFs.
**Figure S17.** Comparison of lowest energy binding poses obtained from AD4 and Vina molecular docking calculations for L-DOPA docked into rigid $\beta_2$AR models.
**Figure S18.** Comparison of lowest energy binding poses obtained from AD4 and Vina molecular docking calculations for L-DOPA docked into flexible $\beta_2$AR models.
**Figure S19.** Comparison of lowest energy binding poses obtained from AD4 and Vina molecular docking calculations for Droxidopa docked into rigid $\beta_2$AR models.
**Figure S20.** Comparison of lowest energy binding poses obtained from AD4 and Vina molecular docking calculations for Droxidopa docked into rigid $\beta_2$AR models.
**Table S5.** Percentage of hydrogen bond formation for different $\beta_2$AR-Ligand complexes from 1 μs all atom MD simulations performed with CHARMM and OPLS AA FF parameters.
**Figure S21.** Percentage of contacts of ligands with the receptor hydrophobic residues extracted from MD simulations of $\beta_2$AR-catecholamine complexes performed with CHARMM and OPLS AA FFs.
**Figure S22.** Hydrogen bond network of adrenaline with key amino acids of $\beta_2$AR binding pocket using CHARMM and OPLS AA FFs.
**Figure S23.** Normalized number of contacts of adrenaline with $\beta_2$AR residues.
**Figure S24.** Comparison of lowest energy binding poses from Vina molecular docking calculations for carbidopa, foslevodopa and foscarbidopa.

**adrenaline.itp:** OPLS AA force field parameters of adrenaline generated with JOYCE.
**dopa.itp:** OPLS AA force field parameters of L-DOPA generated with JOYCE.
**droxidopa.itp:** OPLS AA force field parameters of Droxidopa generated with JOYCE.

**Calculation of torsional profiles with subtracted Coulomb and Lennard-Jones contributions**

The combination rule for the OPLS AA force field is a geometric average of C6 and C12 values in the topology file, which correspond to sigma (in nm) and epsilon (in kJ/mol) values in the ffnonbonded.itp file, as expressed by the following equations:

(S1) $$\sigma_{ij} = \left(\sigma_{ii}\sigma_{jj}\right)^{1/2}$$

(S2) $$\varepsilon_{ij} = \left(\varepsilon_{ii}\varepsilon_{jj}\right)^{1/2}$$

which have been used to estimate Coulomb (electrostatic) and Lennard-Jones (van der Waals) contributions to torsional profiles of dihedral angles of adrenaline, L-DOPA and Droxidopa molecules. The Coulomb and Lennard-Jones energy contributions were calculated using the following equations:

(S3) $$Vc\left(r_{ij}\right) = \frac{q_i q_j}{4\pi\varepsilon_0\varepsilon_r r_{ij}}$$

(S4) $$V_{LJ}\left(r_{ij}\right) = 4\varepsilon_{ij}\left(\frac{\sigma_{ij}}{r_{ij}}\right)^{12} - 4\varepsilon_{ij}\left(\frac{\sigma_{ij}}{r_{ij}}\right)^{6}$$

equations S1 and S2, and the distance matrices obtained as output from Gaussian QM calculations for each dihedral. Minimum and maximum values of electrostatic and LJ energies for each dihedral of adrenaline, L-DOPA and Droxidopa are reported in **Table S4**. Then, electrostatic and Lennard-Jones terms estimated from equations S3 and S4 were subtracted from QM profiles energetic deltas with the fudge (0.5 for QQ and 0.5 for LJ interactions) applied only to 1-4 interactions, which is in good agreement with the usual treatment of these interactions in the OPLS AA force field, using the following equation for the torsional energy rescaling:

(S5) E_new = (QM_E - QM min) - (Q_E - Q_E(QM_min)) - (Q_LJ – Q_LJ(QM_min))

where QM_E is the QM torsional profile energy, QM min is the minimum energy in the QM profile, and Q_E and Q_LJ are eletrostatic and van der Waals contributions, respectively. Finally, these new energetic profiles were used to generate new force field parameters for adrenaline, L-DOPA and Droxidopa molecules by fitting them with linear combinations of cosine functions, depending on the multiplicity of the dihedral angle of interest.

**Figure S1**

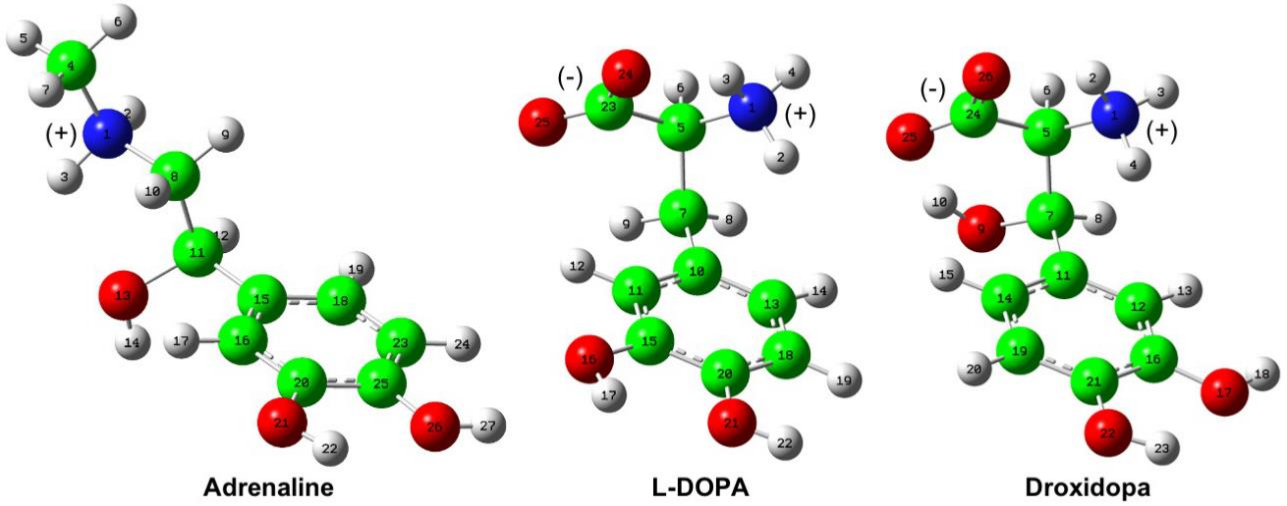


**Figure S1** Geometry optimized chemical structures of protonated adrenaline, zwitterionic L-DOPA and Droxidopa with atom numbers used for the force field development.

**Figure S2**

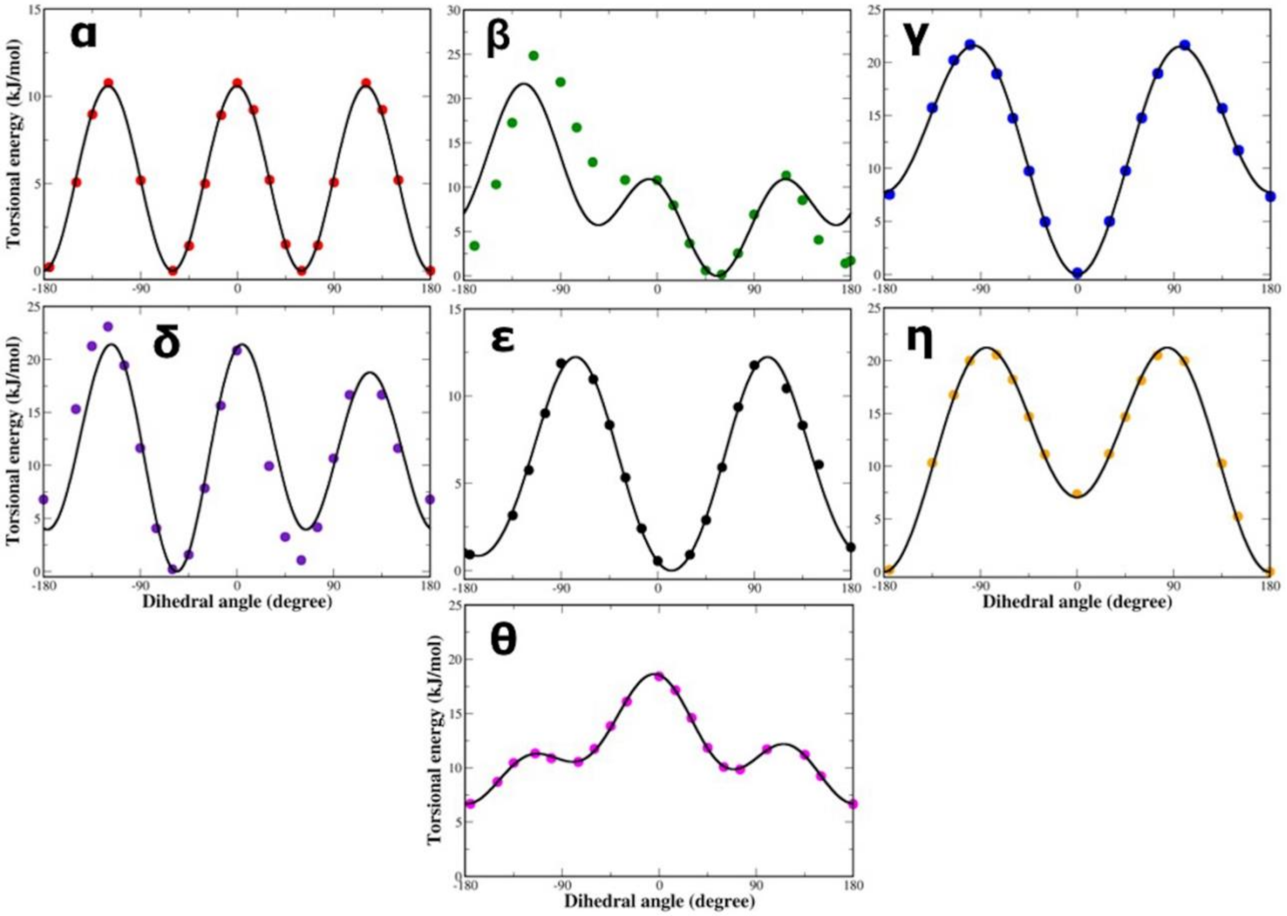


**Figure S2** Torsional profiles derived from QM calculations (dots) and JOYCE (lines) for protonated adrenaline. Each panel refers to dihedral angles defined in Figure 1. Energies are reported in kilojoules per mole (1 kJ/mol = 0.24 kcal/mol).

**Table S1**

Partial atomic charges and OPLS AA force field Lennard-Jones parameters of Adrenaline (ALE).

| Atom number | Atom name | Atom type | Adrenaline | | | |
|---|---|---|---|---|---|---|
| | | | Mass (u) | Charge (e) | σ (nm) | ε (kJ/mol) |
| 1 | N | N | 14.0027 | -0.446343 | 0.325 | 0.71128 |
| 2 | H1 | H | 1.00800 | 0.382023 | 0 | 0 |
| 3 | H2 | H | 1.00800 | 0.363936 | 0 | 0 |
| 4 | C1 | C | 12.0110 | -0.076781 | 0.35 | 0.276144 |
| 5 | H3 | H | 1.00800 | 0.137222 | 0 | 0 |
| 6 | H4 | H | 1.00800 | 0.135202 | 0 | 0 |
| 7 | H5 | H | 1.00800 | 0.135338 | 0 | 0 |
| 8 | CA | CA | 12.0110 | -0.024055 | 0.35 | 0.276144 |
| 9 | HA1 | HA | 1.00800 | 0.138286 | 0.25 | 0.12552 |
| 10 | HA2 | HA | 1.00800 | 0.133064 | 0.25 | 0.12552 |
| 11 | CB | CB | 12.0110 | 0.027731 | 0.35 | 0.276144 |
| 12 | HB1 | HC | 1.00800 | 0.117273 | 0.25 | 0.12552 |
| 13 | OG | OH | 15.9994 | -0.422795 | 0.307 | 0.71128 |
| 14 | HG | HH | 1.00800 | 0.347155 | 0 | 0 |
| 15 | CG | CC | 12.0110 | -0.029545 | 0.355 | 0.29288 |
| 16 | CD1 | CC | 12.0110 | -0.104846 | 0.355 | 0.29288 |
| 17 | HD1 | HC | 1.00800 | 0.107227 | 0.242 | 0.12552 |
| 18 | CD2 | CC | 12.0110 | -0.101815 | 0.355 | 0.29288 |
| 19 | HD2 | HC | 1.00800 | 0.103567 | 0.242 | 0.12552 |
| 20 | CE1 | CC | 12.0110 | 0.073348 | 0.355 | 0.29288 |
| 21 | OE1 | OH | 15.9994 | -0.394652 | 0.307 | 0.71128 |
| 22 | HE1 | HH | 1.00800 | 0.340755 | 0 | 0 |
| 23 | CE2 | CC | 12.0110 | -0.107124 | 0.355 | 0.29288 |
| 24 | HE2 | HC | 1.00800 | 0.112182 | 0.242 | 0.12552 |
| 25 | CZ | CZ | 12.0110 | 0.071847 | 0.355 | 0.29288 |
| 26 | OH | OH | 15.9994 | -0.381798 | 0.307 | 0.71128 |
| 27 | HH | HH | 1.00800 | 0.363632 | 0 | 0 |

**Figure S3**

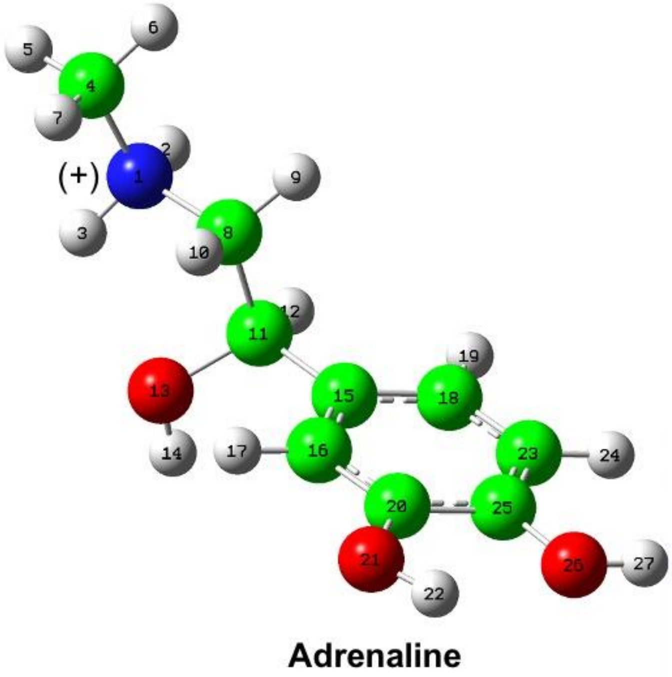


| $a_i$ | $a_j$ | type | $r_{eq}$ (nm) | $k_r$ (kJ/mol nm$^2$) |
|---|---|---|---|---|
| 1 | 2 | 1 | 0.1025 | 393683.477 |
| 1 | 4 | 1 | 0.1496 | 196521.091 |
| 4 | 5 | 1 | 0.1094 | 328457.447 |
| 1 | 8 | 1 | 0.1501 | 179667.704 |
| 8 | 9 | 1 | 0.1094 | 323493.654 |
| 8 | 11 | 1 | 0.1524 | 182391.941 |
| 11 | 12 | 1 | 0.1102 | 301357.367 |
| 11 | 13 | 1 | 0.1436 | 199950.22 |
| 13 | 14 | 1 | 0.0966 | 486572.973 |
| 11 | 15 | 1 | 0.1512 | 205697.663 |
| 15 | 16 | 1 | 0.1403 | 339484.191 |
| 16 | 17 | 1 | 0.109 | 332540.269 |
| 20 | 21 | 1 | 0.1368 | 307423.405 |
| 21 | 22 | 1 | 0.0969 | 480865.258 |
| 25 | 26 | 1 | 0.1372 | 306132.262 |

**Figure S3** OPLS AA force field parameters of adrenaline: bond lengths and force constants. For clarity, only a selection of the most representative bonds is reported in the table. A complete list of bonds parameters can be found in the attached adrenaline.itp file.

**Figure S4**

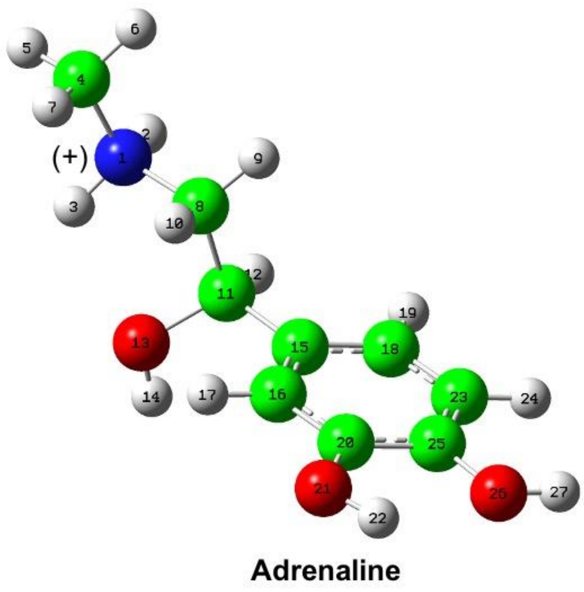


| $a_i$ | $a_j$ | $a_k$ | type | $\theta_{eq}$ (dgr) | $k_\theta$ (kJ/mol rad$^2$) |
|---|---|---|---|---|---|
| 2 | 1 | 3 | 1 | 105.8 | 366.874 |
| 2 | 1 | 4 | 1 | 109.51 | 373.8285 |
| 2 | 1 | 8 | 1 | 109.42 | 381.2262 |
| 1 | 4 | 5 | 1 | 108.48 | 468.3697 |
| 4 | 1 | 8 | 1 | 114.27 | 472.0079 |
| 5 | 4 | 6 | 1 | 110.52 | 309.0556 |
| 1 | 8 | 9 | 1 | 108.31 | 451.2013 |
| 1 | 8 | 11 | 1 | 109.59 | 643.5652 |
| 11 | 15 | 16 | 1 | 120.12 | 588.9987 |
| 15 | 16 | 17 | 1 | 120.97 | 293.3534 |
| 15 | 16 | 20 | 1 | 120.2 | 495.6412 |
| 16 | 20 | 21 | 1 | 119.58 | 534.1722 |
| 16 | 20 | 25 | 1 | 119.91 | 361.9651 |
| 20 | 21 | 22 | 1 | 108.56 | 522.9635 |
| 21 | 20 | 25 | 1 | 120.51 | 771.4094 |

**Figure S4** OPLS AA force field parameters of adrenaline: bond angles and force constants. For clarity, only a selection of the most representative angles is reported in the table. A complete list of angles parameters can be found in the attached adrenaline.itp file.

**Figure S5**

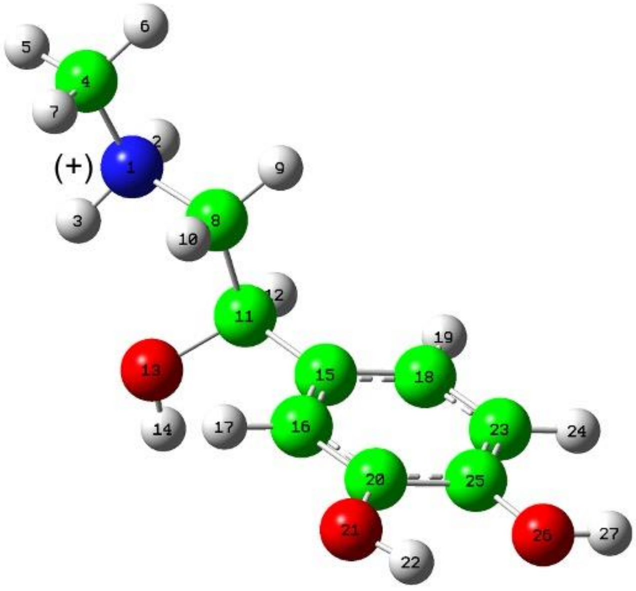


| $a_i$ | $a_j$ | $a_k$ | $a_l$ | type | $\delta_{eq}$ (dgr) | $k_\delta$ (kJ/mol) | n |
|---|---|---|---|---|---|---|---|
| 3 | 1 | 4 | 6 | 1 | 1.11 | 5.3 | 3 |
| 2 | 1 | 8 | 9 | 1 | 270.19 | 5.179 | 1 |
| 2 | 1 | 8 | 9 | 1 | -171.66 | 2.193 | 2 |
| 2 | 1 | 8 | 9 | 1 | 1.25 | 3.196 | 3 |
| 20 | 25 | 26 | 27 | 1 | -179.55 | 3.882 | 1 |
| 20 | 25 | 26 | 27 | 1 | 179.73 | 8.726 | 2 |
| 9 | 8 | 11 | 12 | 1 | -162.12 | 2.055 | 1 |
| 9 | 8 | 11 | 12 | 1 | 63.42 | 1.109 | 2 |
| 9 | 8 | 11 | 12 | 1 | -0.25 | 8.553 | 3 |
| 9 | 8 | 11 | 12 | 1 | -6.16 | 0.163 | 4 |
| 12 | 11 | 15 | 18 | 1 | 180.84 | 0.004 | 1 |
| 12 | 11 | 15 | 18 | 1 | -152.83 | 5.737 | 2 |
| 12 | 11 | 15 | 18 | 1 | 161.46 | 0.004 | 3 |
| 12 | 11 | 15 | 18 | 1 | 17.84 | 0.217 | 4 |
| 16 | 20 | 21 | 22 | 1 | -0.56 | 3.524 | 1 |

**Figure S5** OPLS AA force field parameters of adrenaline: dihedral angles, force constants and multiplicity.

**Table S2**

Partial atomic charges and OPLS AA force field Lennard-Jones parameters of L-DOPA (DAH).

| Atom number | Atom name | Atom type | L-DOPA | | | |
|---|---|---|---|---|---|---|
| | | | Mass (u) | Charge (e) | σ (nm) | ε (kJ/mol) |
| 1 | N | N | 14.0027 | -0.564145 | 0.325 | 0.71128 |
| 2 | H1 | H | 1.00800 | 0.38029 | 0 | 0 |
| 3 | H2 | H | 1.00800 | 0.371986 | 0 | 0 |
| 4 | H3 | H | 1.00800 | 0.328559 | 0 | 0 |
| 5 | CA | CA | 12.0110 | 0.028023 | 0.35 | 0.276144 |
| 6 | HA | HA | 1.00800 | 0.12435 | 0.25 | 0.12552 |
| 7 | CB | CB | 12.0110 | -0.134714 | 0.35 | 0.276144 |
| 8 | HB1 | HA | 1.00800 | 0.102968 | 0.25 | 0.12552 |
| 9 | HB2 | HA | 1.00800 | 0.10481 | 0.25 | 0.12552 |
| 10 | CG | CC | 12.0110 | -0.022917 | 0.355 | 0.29288 |
| 11 | CD1 | CC | 12.0110 | -0.109878 | 0.355 | 0.29288 |
| 12 | HD1 | HC | 1.00800 | 0.104159 | 0.242 | 0.12552 |
| 13 | CD2 | CC | 12.0110 | -0.109974 | 0.355 | 0.29288 |
| 14 | HD2 | HC | 1.00800 | 0.100022 | 0.242 | 0.12552 |
| 15 | CE1 | CC | 12.0110 | 0.067123 | 0.355 | 0.29288 |
| 16 | OE1 | OH | 15.9994 | -0.399226 | 0.307 | 0.71128 |
| 17 | HE1 | HH | 1.00800 | 0.338272 | 0 | 0 |
| 18 | CE2 | CC | 12.0110 | -0.11227 | 0.355 | 0.29288 |
| 19 | HE2 | HC | 1.00800 | 0.108509 | 0.242 | 0.12552 |
| 20 | CZ | CZ | 12.0110 | 0.062947 | 0.355 | 0.29288 |
| 21 | OH | OH | 15.9994 | -0.388243 | 0.307 | 0.71128 |
| 22 | HH | HH | 1.00800 | 0.360275 | 0 | 0 |
| 23 | C | C | 12.0110 | 0.185232 | 0.375 | 0.43932 |
| 24 | O1 | O | 15.9994 | -0.468657 | 0.296 | 0.87864 |
| 25 | O2 | O | 15.9994 | -0.457493 | 0.296 | 0.87864 |

**Figure S6**

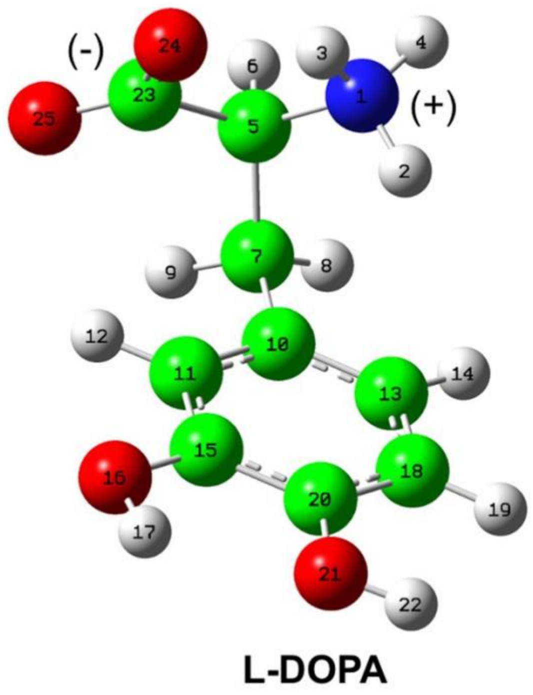


| $a_i$ | $a_j$ | type | $r_{eq}$ (nm) | $k_r$ (kJ/mol nm$^2$) |
|---|---|---|---|---|
| 1 | 2 | 1 | 0.1026 | 368489.034 |
| 1 | 5 | 1 | 0.1506 | 180341.536 |
| 5 | 6 | 1 | 0.1095 | 318058.259 |
| 5 | 7 | 1 | 0.1536 | 180195.043 |
| 7 | 8 | 1 | 0.1098 | 314597.457 |
| 7 | 10 | 1 | 0.1512 | 214106.063 |
| 10 | 11 | 1 | 0.1405 | 332239.63 |
| 11 | 12 | 1 | 0.109 | 331723.534 |
| 15 | 16 | 1 | 0.1368 | 305030.302 |
| 16 | 17 | 1 | 0.0969 | 480979.109 |
| 15 | 20 | 1 | 0.1408 | 309763.805 |
| 20 | 21 | 1 | 0.1374 | 305476.385 |
| 21 | 22 | 1 | 0.0966 | 480979.109 |
| 5 | 23 | 1 | 0.156 | 136630.345 |
| 23 | 24 | 1 | 0.1269 | 478155.015 |

**Figure S6** OPLS AA force field parameters of L-DOPA: bond lengths and force constants. For clarity, only a selection of the most representative bonds is reported in the table. A complete list of bonds parameters can be found in the attached dopa.itp file.

**Figure S7**

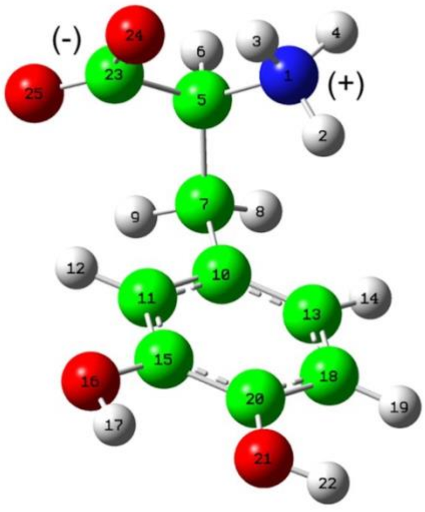


| $a_i$ | $a_j$ | $a_k$ | type | $\theta_{eq}$ (dgr) | $k_\theta$ (kJ/mol rad$^2$) |
|---|---|---|---|---|---|
| 2 | 1 | 3 | 1 | 108.76 | 338.0992 |
| 2 | 1 | 5 | 1 | 111.79 | 354.8638 |
| 1 | 5 | 6 | 1 | 107.34 | 467.6459 |
| 1 | 5 | 7 | 1 | 111.77 | 482.7422 |
| 1 | 5 | 23 | 1 | 105.54 | 528.7628 |
| 6 | 5 | 7 | 1 | 109.7 | 396.4863 |
| 6 | 5 | 23 | 1 | 107.64 | 316.9483 |
| 5 | 7 | 8 | 1 | 109.02 | 342.6356 |
| 5 | 7 | 10 | 1 | 112.82 | 622.4218 |
| 7 | 5 | 23 | 1 | 114.51 | 523.3779 |
| 9 | 7 | 10 | 1 | 111.11 | 348.8275 |
| 7 | 10 | 11 | 1 | 120.42 | 495.9408 |
| 10 | 11 | 12 | 1 | 120.47 | 295.1435 |
| 11 | 10 | 13 | 1 | 118.84 | 539.073 |
| 11 | 15 | 16 | 1 | 119.53 | 574.6185 |
| 11 | 15 | 20 | 1 | 120.05 | 379.8506 |
| 15 | 16 | 17 | 1 | 108.53 | 518.3672 |
| 16 | 15 | 20 | 1 | 120.42 | 796.0599 |
| 13 | 18 | 19 | 1 | 120.37 | 295.1435 |
| 13 | 18 | 20 | 1 | 119.76 | 379.8506 |
| 19 | 18 | 20 | 1 | 119.86 | 327.6687 |
| 15 | 20 | 18 | 1 | 119.9 | 99.2215 |
| 15 | 20 | 21 | 1 | 115.28 | 699.1805 |
| 20 | 21 | 22 | 1 | 110.6 | 449.2292 |
| 5 | 23 | 24 | 1 | 114.7 | 372.9536 |
| 24 | 23 | 25 | 1 | 128.67 | 683.7864 |

**Figure S7** OPLS AA force field parameters of L-DOPA: bond angles and force constants. For clarity, only a selection of the most representative angles is reported in the table. A complete list of angles parameters can be found in the attached dopa.itp file.

**Figure S8**

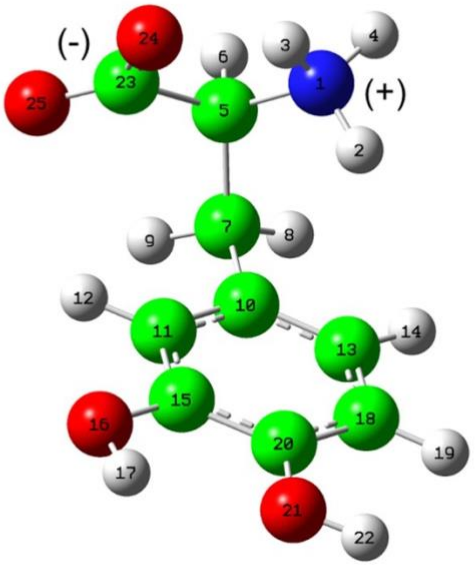


L-DOPA

| $a_i$ | $a_j$ | $a_k$ | $a_l$ | type | $\delta_{eq}$ (dgr) | $k_\delta$ (kJ/mol) | n |
|---|---|---|---|---|---|---|---|
| 6 | 5 | 23 | 24 | 1 | 320.79 | 9.731 | 2 |
| 6 | 5 | 23 | 25 | 1 | 318.43 | 9.731 | 2 |
| 2 | 1 | 5 | 6 | 1 | 127.76 | 2.482 | 3 |
| 3 | 1 | 5 | 6 | 1 | 140.68 | 2.482 | 3 |
| 18 | 20 | 21 | 22 | 1 | 0 | 3.251 | 1 |
| 18 | 20 | 21 | 22 | 1 | 179.99 | 8.441 | 2 |
| 6 | 5 | 7 | 8 | 1 | -193.73 | 5.697 | 1 |
| 6 | 5 | 7 | 8 | 1 | -94.39 | 1.592 | 2 |
| 6 | 5 | 7 | 8 | 1 | -14.05 | 6.757 | 3 |
| 6 | 5 | 7 | 8 | 1 | 8.32 | 0.158 | 4 |
| 8 | 7 | 10 | 13 | 1 | 242.4 | 0.135 | 1 |
| 8 | 7 | 10 | 13 | 1 | 199.06 | 8.06 | 2 |
| 8 | 7 | 10 | 13 | 1 | 252.02 | 0.038 | 3 |
| 8 | 7 | 10 | 13 | 1 | -62.1 | 2.03 | 4 |
| 8 | 7 | 10 | 11 | 1 | 242.4 | 0.135 | 1 |
| 8 | 7 | 10 | 11 | 1 | 199.06 | 8.06 | 2 |
| 8 | 7 | 10 | 11 | 1 | 252.02 | 0.038 | 3 |
| 8 | 7 | 10 | 11 | 1 | -62.1 | 2.03 | 4 |
| 11 | 15 | 16 | 17 | 1 | 359.82 | 3.153 | 1 |
| 11 | 15 | 16 | 17 | 1 | 179.64 | 9.114 | 2 |

**Figure S8** OPLS AA force field parameters of L-DOPA: dihedral angles, force constants and multiplicity.

**Table S3**

Partial atomic charges and OPLS AA force field Lennard-Jones parameters of Droxidopa (DRO).

| Atom number | Atom name | Atom type | Droxidopa | | | |
|---|---|---|---|---|---|---|
| | | | Mass (u) | Charge (e) | σ (nm) | ε (kJ/mol) |
| 1 | N | N | 14.0027 | -0.563874 | 0.325 | 0.71128 |
| 2 | H1 | H | 1.00800 | 0.334884 | 0 | 0 |
| 3 | H2 | H | 1.00800 | 0.383193 | 0 | 0 |
| 4 | H3 | H | 1.00800 | 0.347978 | 0 | 0 |
| 5 | CA | CA | 12.0110 | 0.025501 | 0.35 | 0.276144 |
| 6 | HA | HA | 1.00800 | 0.138072 | 0.25 | 0.12552 |
| 7 | CB | CB | 12.0110 | 0.018493 | 0.35 | 0.276144 |
| 8 | HB1 | HA | 1.00800 | 0.11258 | 0.25 | 0.12552 |
| 9 | OG | OH | 15.9994 | -0.469561 | 0.307 | 0.71128 |
| 10 | HG | HO | 1.00800 | 0.286741 | 0 | 0 |
| 11 | CG | CC | 12.0110 | -0.037767 | 0.355 | 0.29288 |
| 12 | CD1 | CC | 12.0110 | -0.106979 | 0.355 | 0.29288 |
| 13 | HD1 | HC | 1.00800 | 0.113467 | 0.242 | 0.12552 |
| 14 | CD2 | CC | 12.0110 | -0.106878 | 0.355 | 0.29288 |
| 15 | HD2 | HC | 1.00800 | 0.096655 | 0.242 | 0.12552 |
| 16 | CE1 | CC | 12.0110 | 0.071455 | 0.355 | 0.29288 |
| 17 | OE1 | OH | 15.9994 | -0.382444 | 0.307 | 0.71128 |
| 18 | HE1 | HH | 1.00800 | 0.363284 | 0 | 0 |
| 19 | CE2 | CC | 12.0110 | -0.103248 | 0.355 | 0.29288 |
| 20 | HE2 | HC | 1.00800 | 0.108686 | 0.242 | 0.12552 |
| 21 | CZ | CZ | 12.0110 | 0.073032 | 0.355 | 0.29288 |
| 22 | OH | OH | 15.9994 | -0.393531 | 0.307 | 0.71128 |
| 23 | HH | HH | 1.00800 | 0.341308 | 0 | 0 |
| 24 | C | C | 12.0110 | 0.201167 | 0.375 | 0.43932 |
| 25 | O1 | O | 15.9994 | -0.421581 | 0.296 | 0.87864 |
| 26 | O2 | O | 15.9994 | -0.430621 | 0.296 | 0.87864 |

**Figure S9**

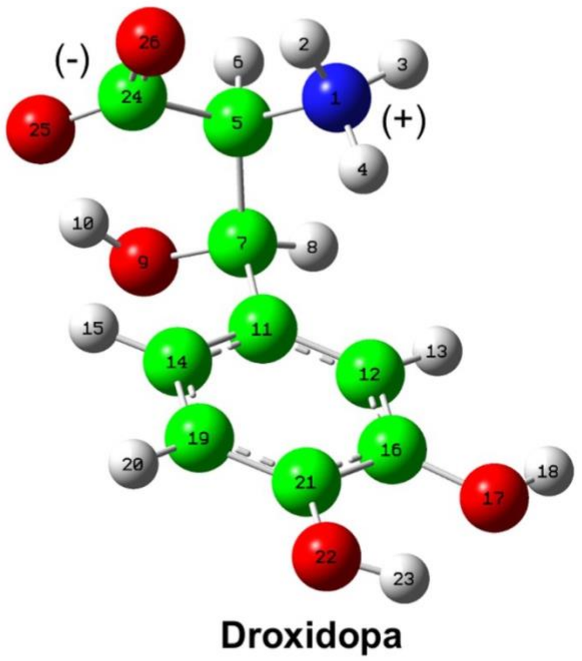


| $a_i$ | $a_j$ | type | $r_{eq}$ (nm) | $k_r$ (kJ/mol nm$^2$) |
|---|---|---|---|---|
| 1 | 2 | 1 | 0.1048 | 373470.846 |
| 1 | 5 | 1 | 0.1499 | 207081.212 |
| 5 | 6 | 1 | 0.1096 | 317299.775 |
| 5 | 7 | 1 | 0.1551 | 154162.502 |
| 7 | 8 | 1 | 0.1099 | 305252.01 |
| 7 | 9 | 1 | 0.142 | 225968.096 |
| 9 | 10 | 1 | 0.0987 | 383776.997 |
| 7 | 11 | 1 | 0.152 | 208485.422 |
| 11 | 12 | 1 | 0.1404 | 316021.4 |
| 12 | 13 | 1 | 0.1093 | 332268.673 |
| 16 | 17 | 1 | 0.1372 | 289583.572 |
| 17 | 18 | 1 | 0.0966 | 481033.117 |
| 16 | 21 | 1 | 0.1404 | 321359.557 |
| 5 | 24 | 1 | 0.1558 | 132339.541 |
| 24 | 25 | 1 | 0.1259 | 479501.584 |

**Figure S9** OPLS AA force field parameters of Droxidopa: bond lengths and force constants. For clarity, only a selection of the most representative bonds is reported in the table. A complete list of bonds parameters can be found in the attached droxidopa.itp file.

**Figure S10**

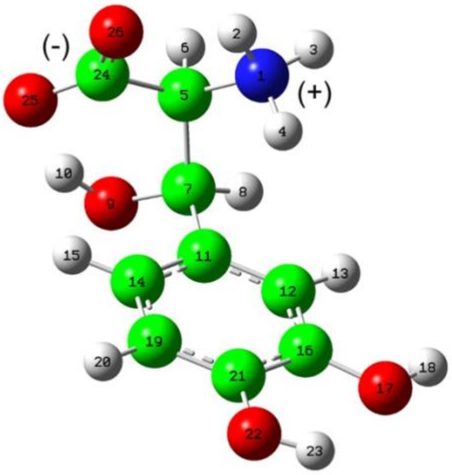


| $a_i$ | $a_j$ | $a_k$ | type | $\theta_{eq}$ (dgr) | $k_\theta$ (kJ/mol rad$^2$) |
|---|---|---|---|---|---|
| 2 | 1 | 3 | 1 | 111.49 | 343.4745 |
| 2 | 1 | 5 | 1 | 102.59 | 361.7045 |
| 1 | 5 | 6 | 1 | 107.66 | 519.9423 |
| 1 | 5 | 7 | 1 | 111.87 | 553.7967 |
| 1 | 5 | 24 | 1 | 105.89 | 484.0496 |
| 6 | 5 | 7 | 1 | 108.85 | 417.8032 |
| 6 | 5 | 24 | 1 | 107.9 | 291.6417 |
| 5 | 7 | 8 | 1 | 107.6 | 419.7621 |
| 5 | 7 | 9 | 1 | 108.48 | 744.6744 |
| 5 | 7 | 11 | 1 | 111.66 | 365.07 |
| 7 | 5 | 24 | 1 | 114.39 | 468.9387 |
| 8 | 7 | 9 | 1 | 106.95 | 492.3242 |
| 8 | 7 | 11 | 1 | 107.98 | 276.5572 |
| 7 | 9 | 10 | 1 | 105.6 | 632.6532 |
| 9 | 7 | 11 | 1 | 113.89 | 449.8951 |
| 7 | 11 | 12 | 1 | 119.34 | 687.2295 |
| 11 | 12 | 13 | 1 | 120.26 | 298.4541 |
| 12 | 11 | 14 | 1 | 119.01 | 496.0446 |
| 12 | 16 | 17 | 1 | 124.4 | 731.9646 |
| 12 | 16 | 21 | 1 | 120.09 | 377.2683 |
| 16 | 17 | 18 | 1 | 110.69 | 453.9904 |
| 17 | 16 | 21 | 1 | 115.5 | 634.4644 |
| 20 | 19 | 21 | 1 | 118.8 | 323.2446 |
| 16 | 21 | 19 | 1 | 119.52 | 209.44 |
| 16 | 21 | 22 | 1 | 120.77 | 671.056 |
| 21 | 22 | 23 | 1 | 108.62 | 481.9262 |
| 5 | 24 | 25 | 1 | 116.12 | 324.2452 |
| 25 | 24 | 26 | 1 | 128.37 | 813.2475 |

**Figure S10** OPLS AA force field parameters of Droxidopa: bond angles and force constants. For clarity, only a selection of the most representative angles is reported in the table. A complete list of angles parameters can be found in the attached droxidopa.itp file.

**Figure S11**

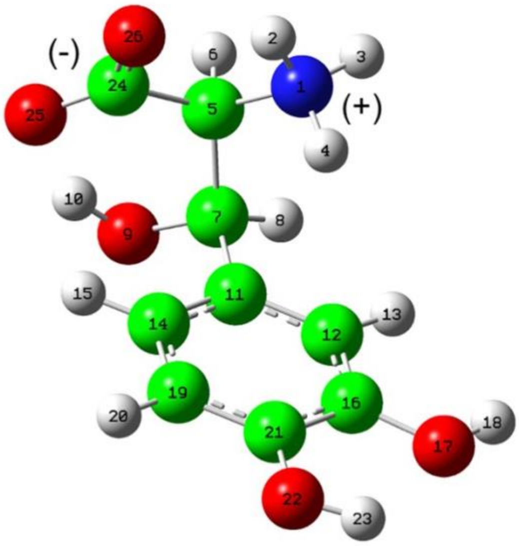


| $a_i$ | $a_j$ | $a_k$ | $a_l$ | type | $\delta_{eq}$ (dgr) | $k_\delta$ (kJ/mol) | n |
|---|---|---|---|---|---|---|---|
| 6 | 5 | 24 | 25 | 1 | 328.39 | 10.451 | 2 |
| 6 | 5 | 24 | 26 | 1 | 330.59 | 10.451 | 2 |
| 2 | 1 | 5 | 6 | 1 | 125.59 | 2.547 | 3 |
| 3 | 1 | 5 | 6 | 1 | 123.45 | 2.547 | 3 |
| 19 | 21 | 22 | 23 | 1 | 359.86 | 3.272 | 1 |
| 19 | 21 | 22 | 23 | 1 | 179.73 | 8.853 | 2 |
| 6 | 5 | 7 | 8 | 1 | -218.42 | 3.367 | 1 |
| 6 | 5 | 7 | 8 | 1 | -87.74 | 9.305 | 2 |
| 6 | 5 | 7 | 8 | 1 | -25.06 | 9.134 | 3 |
| 6 | 5 | 7 | 8 | 1 | -144.67 | 0.009 | 4 |
| 8 | 7 | 11 | 14 | 1 | -120.9 | 0.702 | 1 |
| 8 | 7 | 11 | 14 | 1 | 185.55 | 10.437 | 2 |
| 8 | 7 | 11 | 14 | 1 | 41.83 | 0.182 | 3 |
| 8 | 7 | 11 | 14 | 1 | 0 | 0.042 | 4 |
| 8 | 7 | 11 | 12 | 1 | -120.9 | 0.702 | 1 |
| 8 | 7 | 11 | 12 | 1 | 185.55 | 10.437 | 2 |
| 8 | 7 | 11 | 12 | 1 | 41.83 | 0.182 | 3 |
| 8 | 7 | 11 | 12 | 1 | 0 | 0.042 | 4 |
| 12 | 16 | 17 | 18 | 1 | 0 | 3.272 | 1 |
| 12 | 16 | 17 | 18 | 1 | 179.99 | 8.853 | 2 |
| 5 | 7 | 9 | 10 | 1 | -215.05 | 12.202 | 1 |
| 5 | 7 | 9 | 10 | 1 | 128.96 | 6.72 | 2 |
| 5 | 7 | 9 | 10 | 1 | 777.43 | 2.465 | 3 |
| 5 | 7 | 9 | 10 | 1 | 43.77 | 0.432 | 4 |

**Figure S11** OPLS AA force field parameters of Droxidopa: dihedral angles, force constants and multiplicity.

**Figure S12**

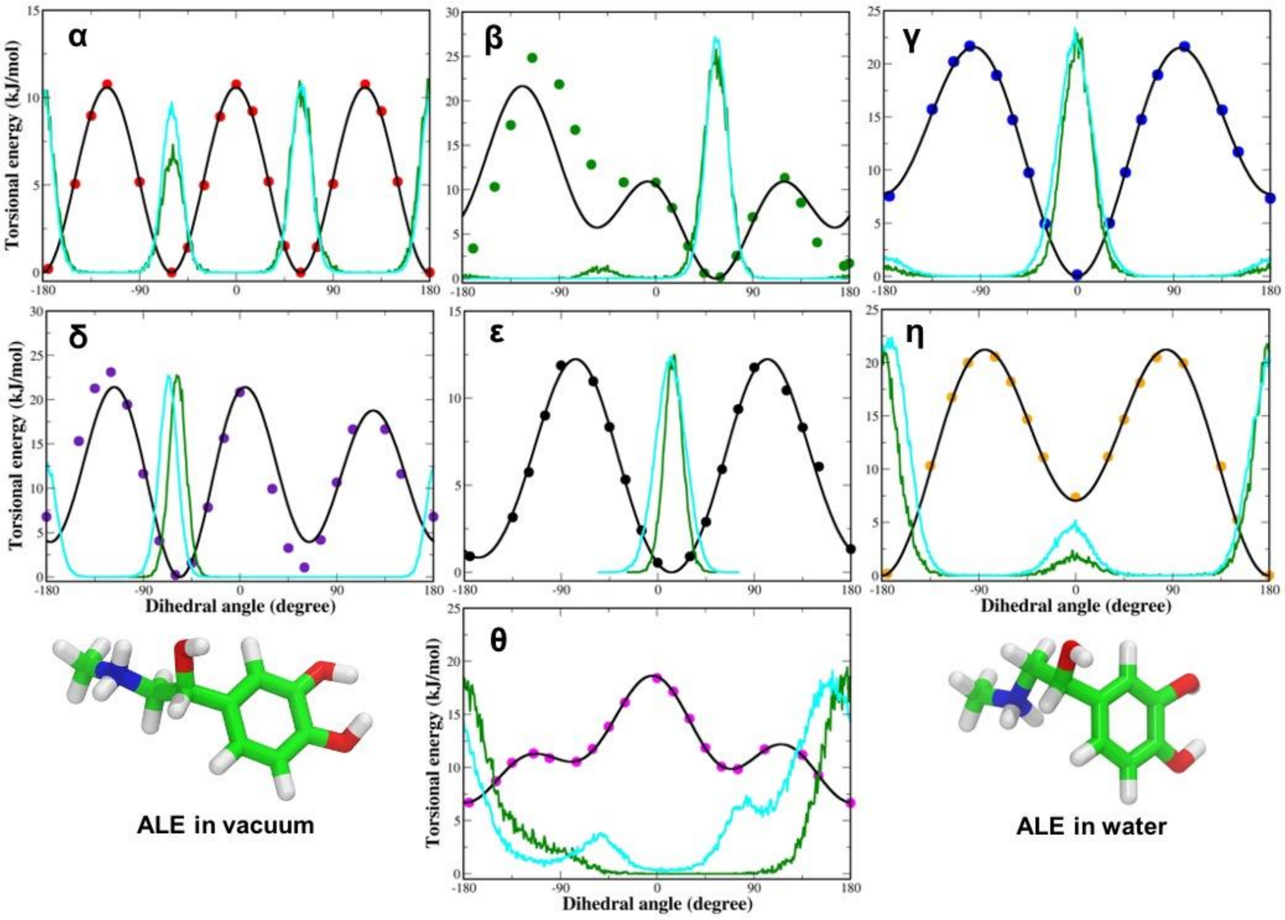


**Figure S12** (Top) Dihedrals distributions in vacuum (green) and water (cyan) are compared to torsional profiles obtained by QM calculations (dots) and JOYCE (lines) for protonated adrenaline. Each panel refers to dihedral angles defined in Figure 1. (Bottom) Adrenaline structures simulated for 20 ns and 100 ns in vacuum and water, respectively. Energies are reported in kilojoules per mole (1 kJ/mol = 0.24 kcal/mol).

**Table S4**

Minimum and maximum values of Coulomb ($V_C$) and Lennard-Jones ($V_{LJ}$) energies of each dihedral of Adrenaline, L-DOPA and Droxidopa molecules.

| Ligand | Dihedral | $V_C$ (kJ/mol) | $V_{LJ}$ (kJ/mol) |
|---|---|---|---|
| **Adrenaline** | α | 1.28 ÷ 1.30 | -30.8 ÷ -30.1 |
| | β | 1.23 ÷ 1.47 | -32.6 ÷ -29.4 |
| | γ | 1.34 ÷ 1.66 | -31.1 ÷ -29.6 |
| | δ | 1.04 ÷ 1.71 | -41.6 ÷ -26.8 |
| | ε | 1.29 ÷ 1.44 | -37.5 ÷ -30.6 |
| | η | 1.29 ÷ 1.66 | -31.2 ÷ -29.4 |
| | θ | 1.63 ÷ 1.93 | -27.2 ÷ -25.8 |
| **L-DOPA** | α | -3.7 ÷ -4.2 | -27.4 ÷ -41.4 |
| | β | -3.8 ÷ -4.2 | -37.3 ÷ -41.7 |
| | γ | -4.1 ÷ -4.5 | -41.0 ÷ -42.5 |
| | δ | -3.9 ÷ -4.5 | -35.0 ÷ -44.5 |
| | ε | -4.1 ÷ -4.4 | -39.5 ÷ -50.5 |
| | η | -4.1 ÷ -4.2 | -40.5 ÷ -42.5 |
| **Droxidopa** | α | -3.4 ÷ -3.8 | -36.4 ÷ -50.7 |
| | β | -3.5 ÷ -3.9 | -48.7 ÷ -51.2 |
| | γ | -3.6 ÷ -3.8 | -50.0 ÷ -51.3 |
| | δ | -3.6 ÷ -4.6 | -36.6 ÷ -59.2 |
| | ε | -3.6 ÷ -4.1 | -50.8 ÷ -61.5 |
| | η | -3.6 ÷ -4.0 | -49.6 ÷ -51.3 |
| | θ | -3.4 ÷ -4.1 | -45.6 ÷ -51.5 |

**Figure S13**

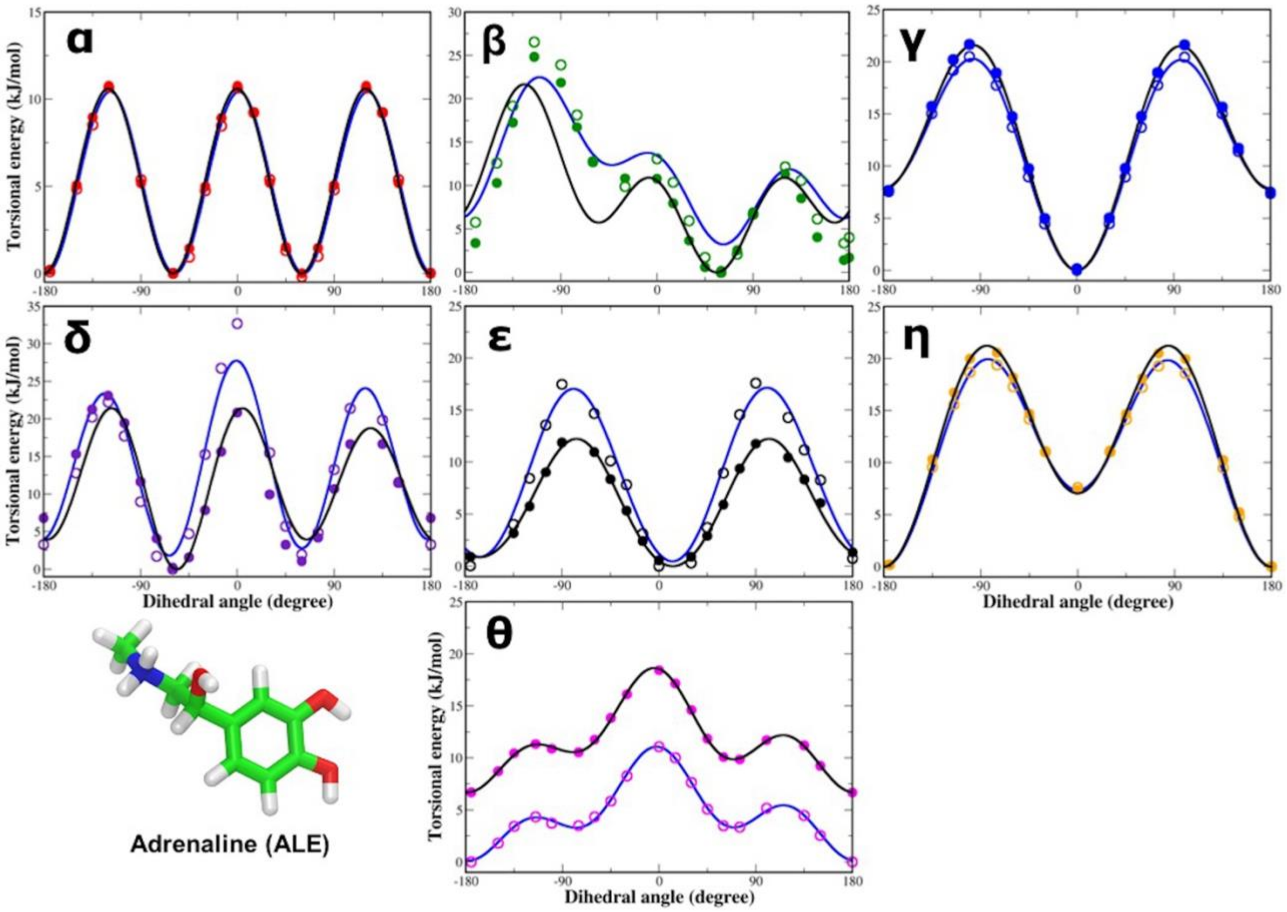


**Figure S13** Comparison of protonated adrenaline dihedral angles torsional energy profiles from QM (closed circles), JOYCE (black lines), QM after the subtraction of electrostatic and Lennard-Jones energy contributions (open circles) and fittings (blue lines) calculations. Energies are reported in kilojoules per mole (1 kJ/mol = 0.24 kcal/mol).

**Figure S14**

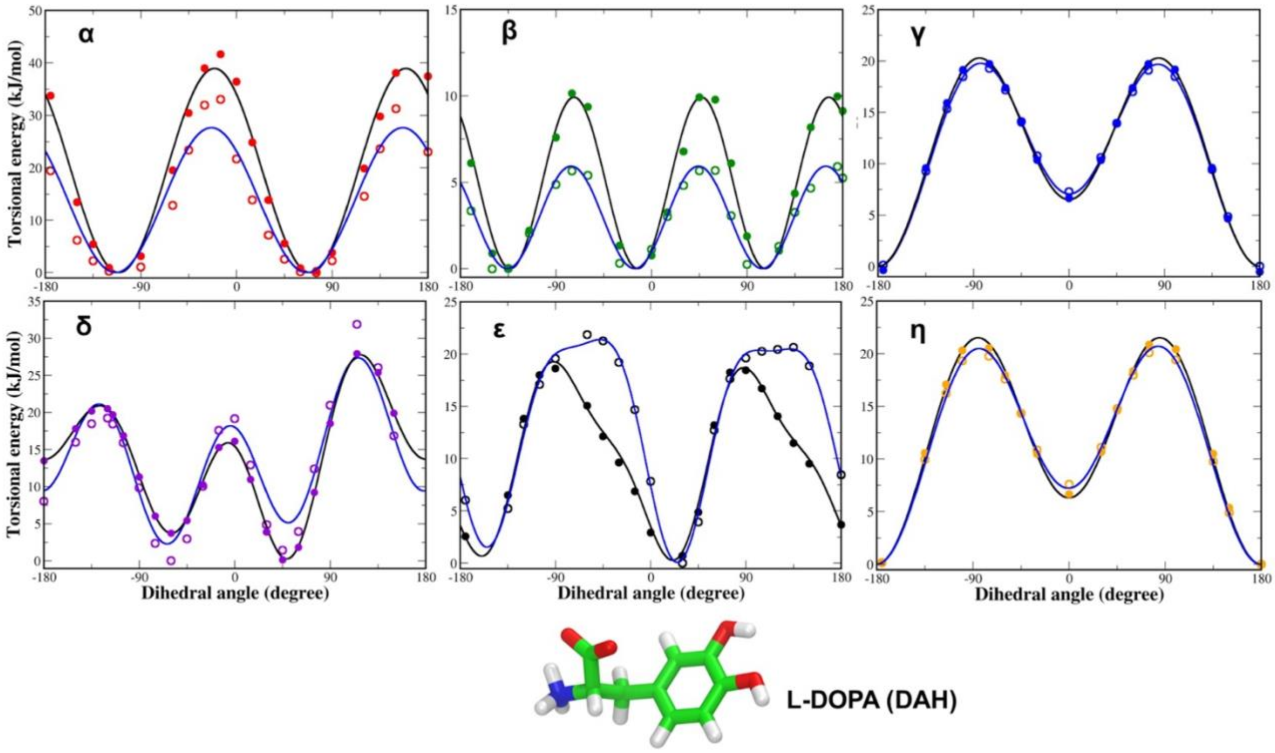


**Figure S14** Comparison of zwitterionic L-DOPA dihedral angles torsional energy profiles from QM (closed circles), JOYCE (black lines), QM after the subtraction of electrostatic and Lennard-Jones energy contributions (open circles) and fittings (blue lines) calculations. Energies are reported in kilojoules per mole (1 kJ/mol = 0.24 kcal/mol).

**Figure S15**

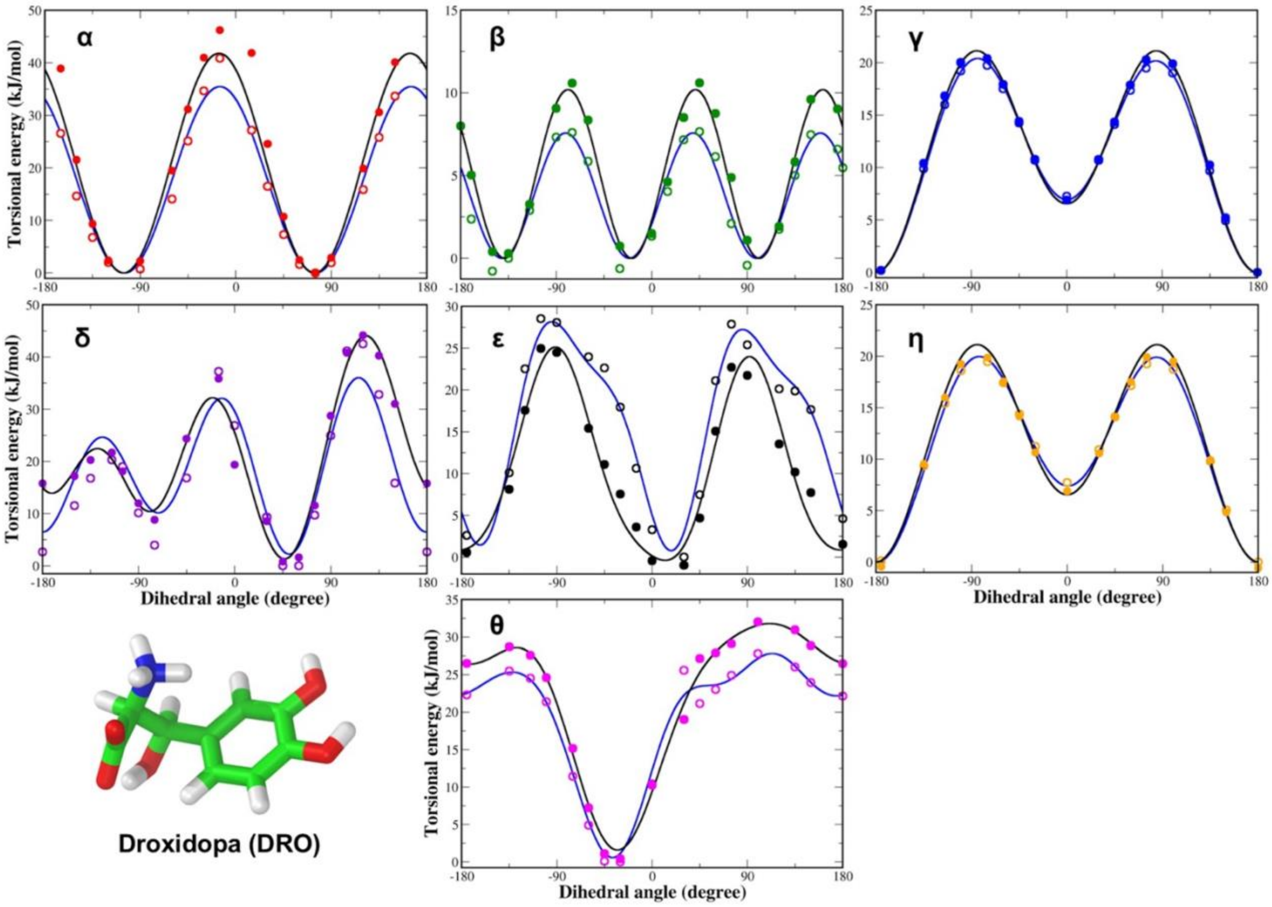


**Figure S15** Comparison of zwitterionic Droxidopa dihedral angles torsional energy profiles from QM (closed circles), JOYCE (black lines), QM after the subtraction of electrostatic and Lennard-Jones energy contributions (open circles) and fittings (blue lines) calculations. Energies are reported in kilojoules per mole (1 kJ/mol = 0.24 kcal/mol).

**Figure S16**

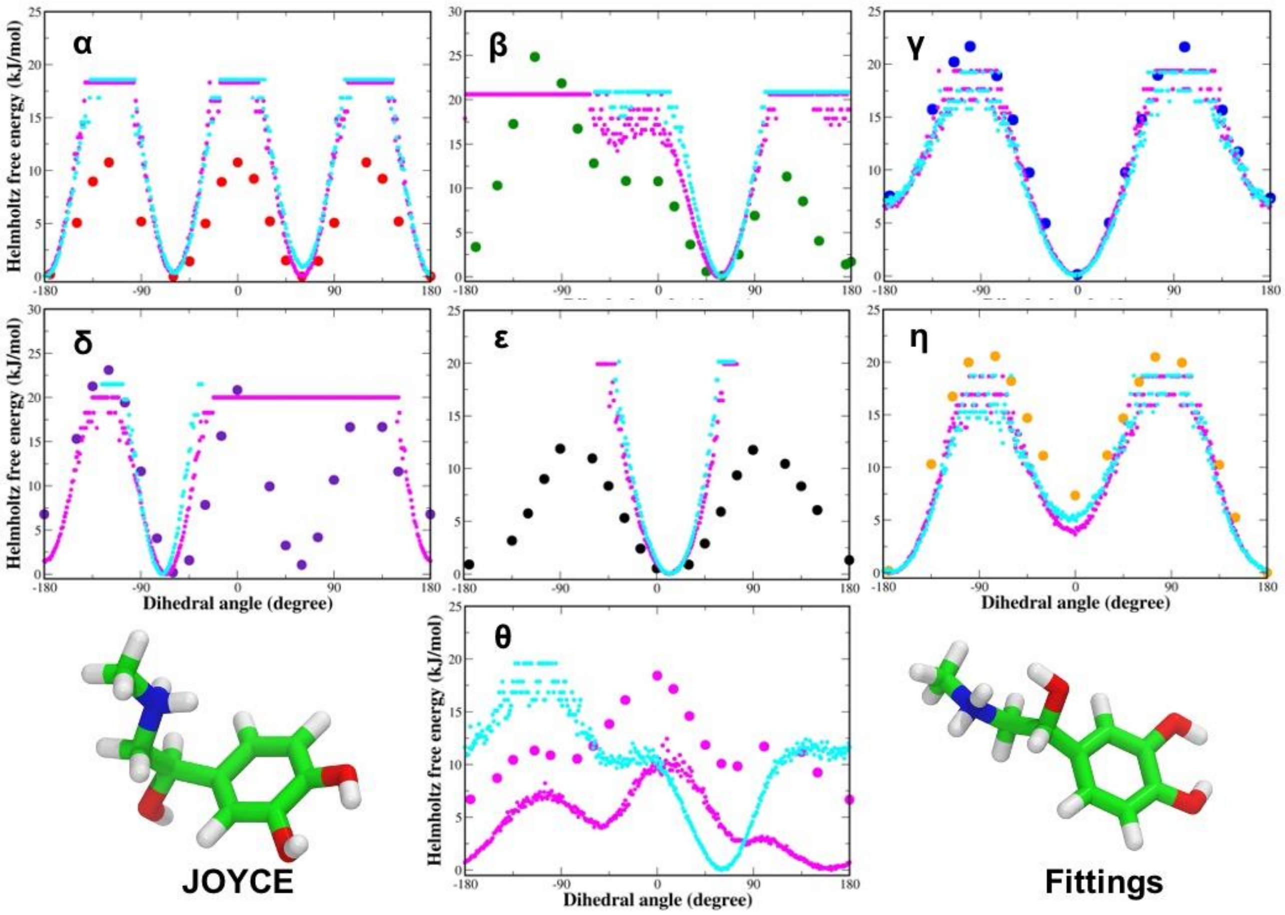


**Figure S16** (Top) Protonated adrenaline QM torsional energy profiles (circles) compared with Helmholtz free energy variations as a function of the dihedral angle, as obtained from MD simulations performed using JOYCE (magenta dots) and OPLS (cyan dots) FFs. (Bottom) 100 ns structures of adrenaline from MD simulations using JOYCE and OPLS (Fittings) FF parameters, respectively. Energies are reported in kilojoules per mole (1 kJ/mol = 0.24 kcal/mol).

**Figure S17**

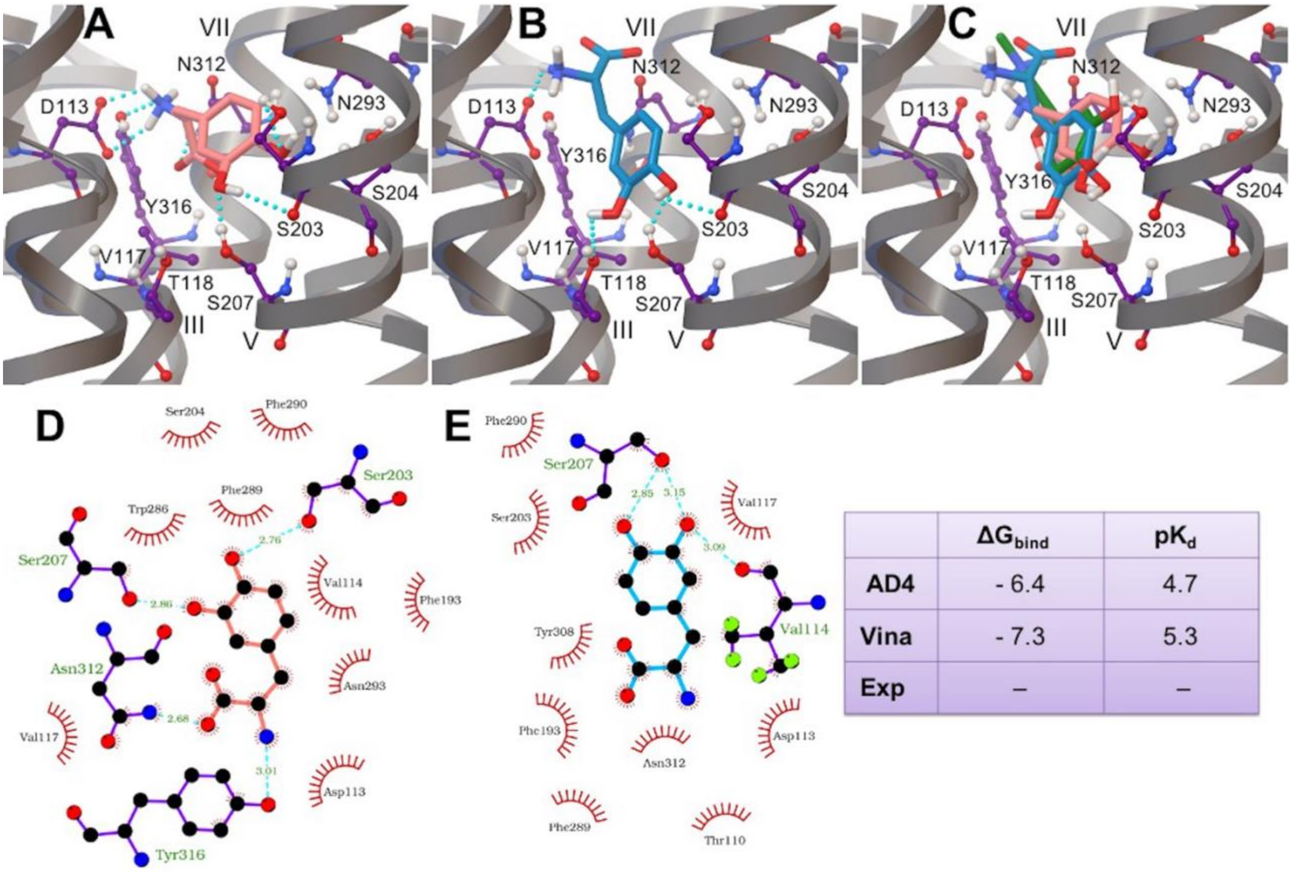


| | ΔG$_{bind}$ | pK$_d$ |
|---|---|---|
| AD4 | - 6.4 | 4.7 |
| Vina | - 7.3 | 5.3 |
| Exp | – | – |

**Figure S17** Lowest energy conformations of L-DOPA obtained from the docking of the ligand into rigid $\beta_2$AR models performed with molecular docking software packages AutoDock4.2 and AutoDock Vina are shown in magenta (A, AD4) and skyblue (B, Vina), respectively. (C) Lowest energy poses of L-DOPA are compared to adrenaline's conformation in the X-ray crystal structure (PDB ID: 4LDO, ligand in dark green). Color code and view point as in Figure 9. Hydrogen bond network of L-DOPA lowest energy conformations generated by (D) AD4 and (E) Vina calculations. Free energies of binding (in kcal/mol) and binding affinities estimated by AD4 and Vina calculations are compared to experimental results.

**Figure S18**

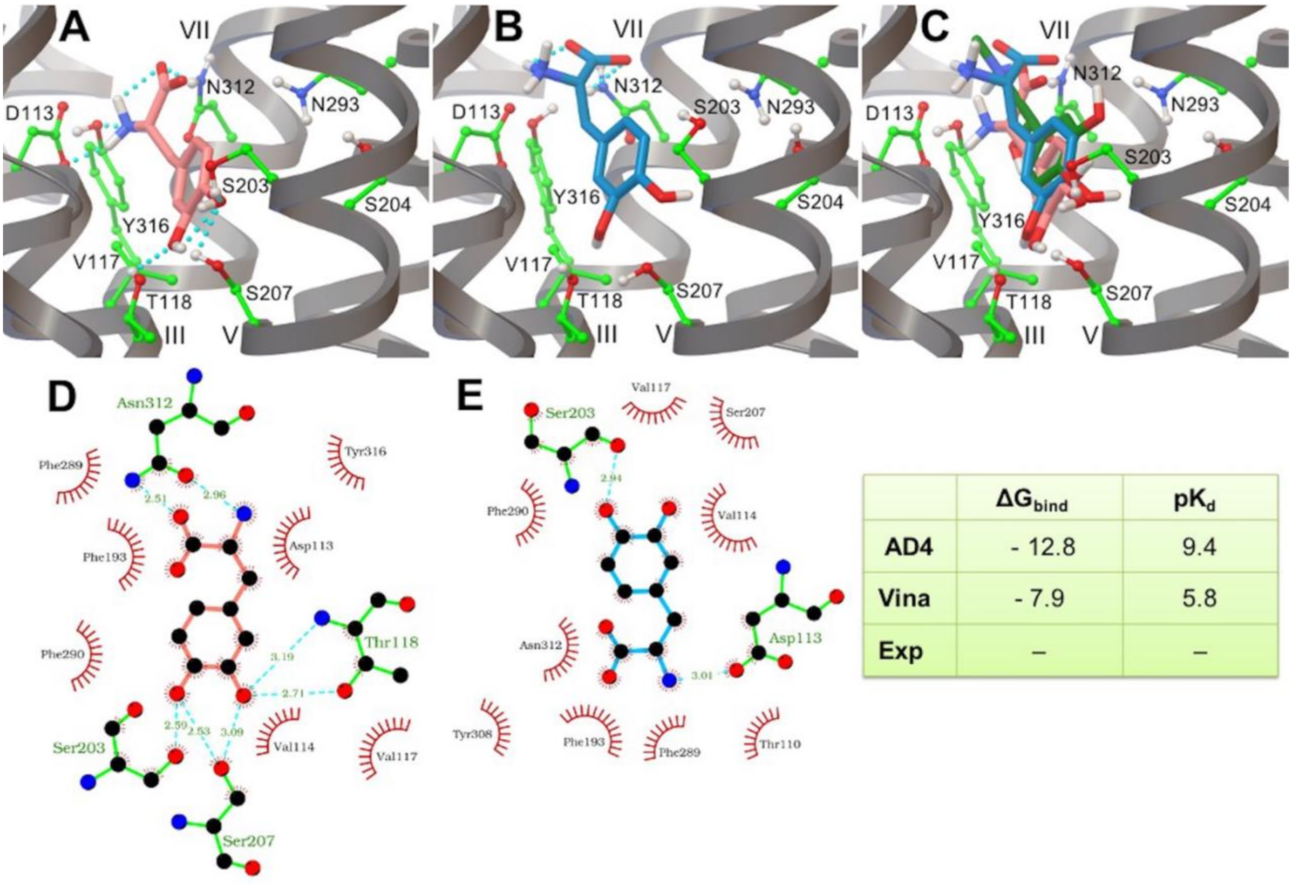


| | $\Delta G_{bind}$ | $pK_d$ |
|---|---|---|
| **AD4** | - 12.8 | 9.4 |
| **Vina** | - 7.9 | 5.8 |
| **Exp** | – | – |

**Figure S18** Lowest energy conformations of L-DOPA obtained from the docking of the ligand into flexible β2AR models performed with molecular docking software packages AutoDock4.2 and AutoDock Vina are shown in magenta (A, AD4) and skyblue (B, Vina), respectively. (C) Lowest energy poses of L-DOPA are compared to adrenaline’s conformation in the X-ray crystal structure (PDB ID: 4LDO, ligand in dark green). Color code and view point as in Figure 9. Hydrogen bond network of L-DOPA lowest energy conformations generated by (D) AD4 and (E) Vina calculations. Free energies of binding (in kcal/mol) and binding affinities estimated by AD4 and Vina calculations are compared to experimental results.

**Figure S19**

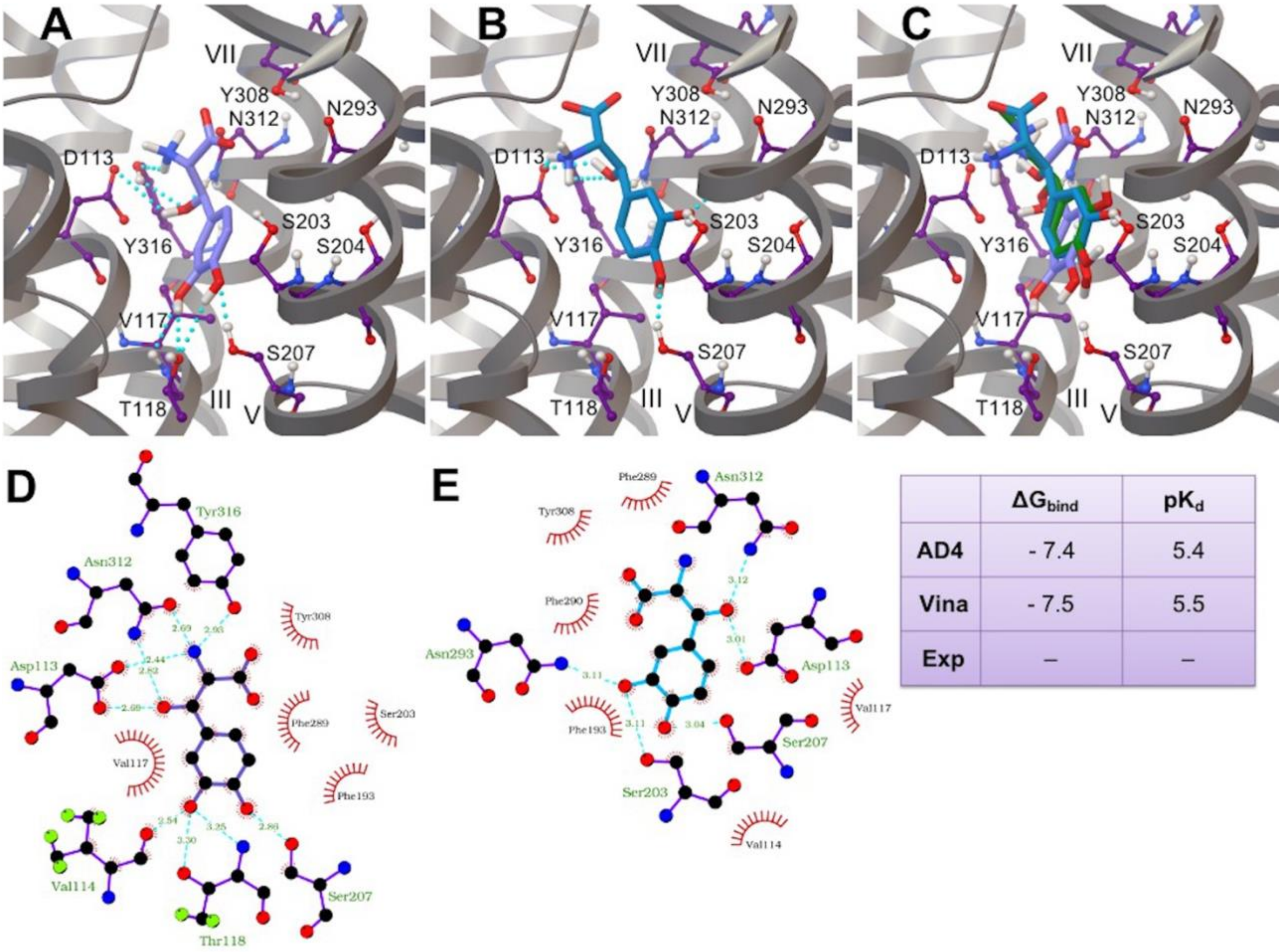


| | $\Delta G_{bind}$ | $pK_d$ |
|---|---|---|
| **AD4** | - 7.4 | 5.4 |
| **Vina** | - 7.5 | 5.5 |
| **Exp** | – | – |

**Figure S19** Lowest energy conformations of Droxidopa obtained from the docking of the ligand into rigid $\beta_2$AR models performed with molecular docking software packages AutoDock4.2 and AutoDock Vina are shown in violet (A, AD4) and skyblue (B, Vina), respectively. (C) Lowest energy poses of Droxidopa are compared to adrenaline's conformation in the X-ray crystal structure (PDB ID: 4LDO, ligand in dark green). Color code and view point as in Figure 9. Hydrogen bond network of Droxidopa lowest energy conformations generated by (D) AD4 and (E) Vina calculations. Free energies of binding (in kcal/mol) and binding affinities estimated by AD4 and Vina calculations are compared to experimental results.

**Figure S20**

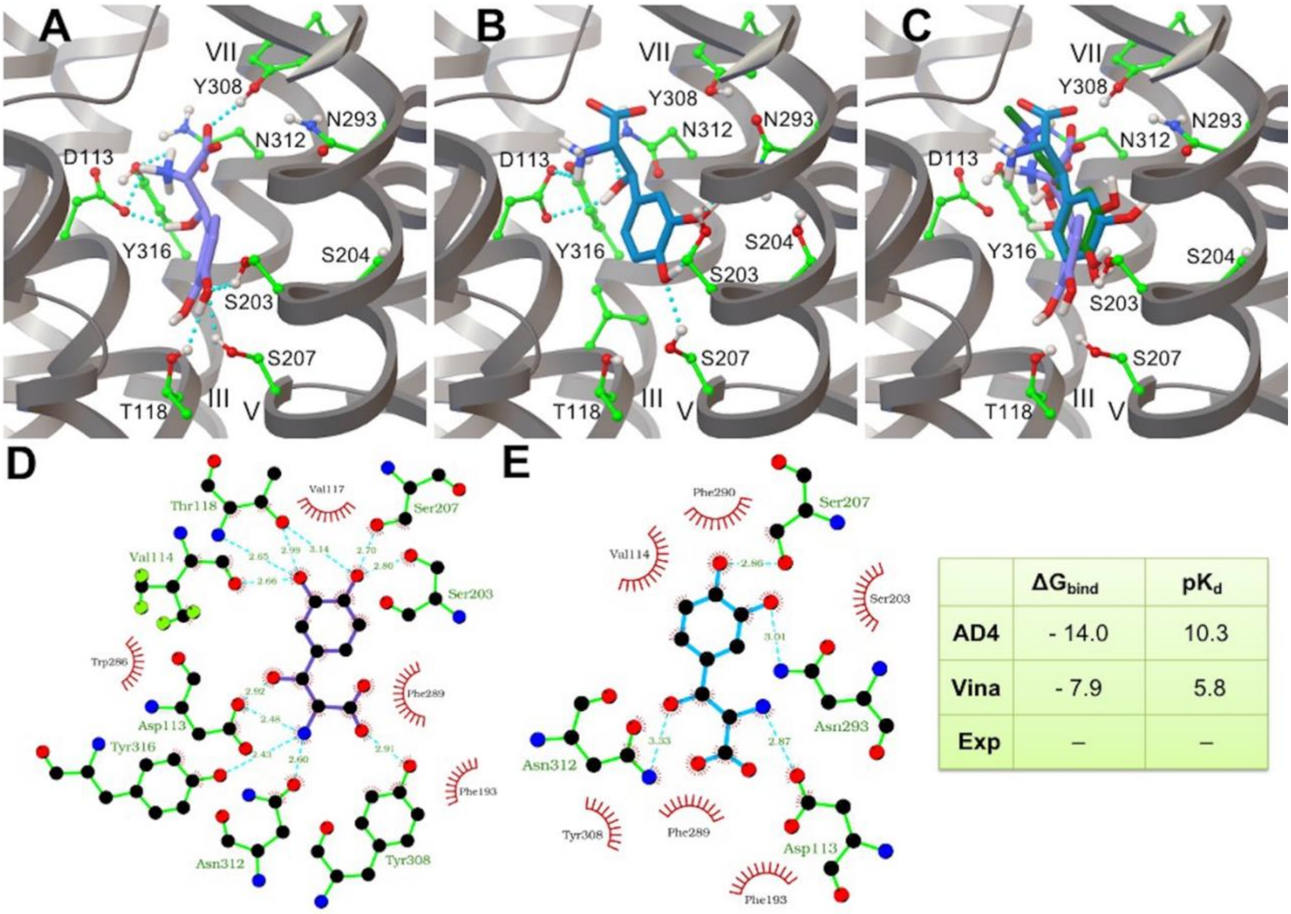


| | ΔG$_{bind}$ | pK$_d$ |
|---|---|---|
| AD4 | - 14.0 | 10.3 |
| Vina | - 7.9 | 5.8 |
| Exp | – | – |

**Figure S20** Lowest energy conformations of Droxidopa obtained from the docking of the ligand into flexible β2AR models performed with molecular docking software packages AutoDock4.2 and AutoDock Vina are shown in violet (A, AD4) and skyblue (B, Vina), respectively. (C) Lowest energy poses of Droxidopa are compared to adrenaline's conformation in the X-ray crystal structure (PDB ID: 4LDO, ligand in dark green). Color code and view point as in Figure 9. Hydrogen bond network of Droxidopa lowest energy conformations generated by (D) AD4 and (E) Vina calculations. Free energies of binding (in kcal/mol) and binding affinities estimated by AD4 and Vina calculations are compared to experimental results.

**Table S5**

Percentage of hydrogen bond formation for different β2AR-Ligand complexes from 1 μs all atom MD simulations performed with CHARMM and OPLS AA FF parameters. The analysis was performed over the whole trajectory for adrenaline (ALE), L-DOPA (DAH) and Droxidopa (DRO) ligands.

| Hydrogen bonds (β2AR-Ligand) | Percentage of H-bond formation (%) | | | | | |
|---|---|---|---|---|---|---|
| | CHARMM | | | OPLS | | |
| | **ALE** | **DAH** | **DRO** | **ALE** | **DAH** | **DRO** |
| OG (S203) - O (para) | 23.6 | 28.4 | 11.9 | 12.6 | 3.5 | 7.4 |
| OG (S203) - O (meta) | **54.3** | 15.3 | 12.4 | **4.4** | 0.3 | 20.7 |
| OG (S204) - O (para) | 0.1 | 11.4 | 2.4 | 0.6 | 0.3 | 0.8 |
| OG (S204) - O (meta) | 1.3 | 31.9 | 20.9 | 0 | 0 | 39.9 |
| OG (S207) - O (para) | **47.9** | 32.0 | 44.3 | **19.3** | 21.4 | 7.6 |
| OG (S207) - O (meta) | 0 | 11.2 | 7.6 | 9.1 | 7.2 | 0.9 |
| ND2 (N293) - O (para) | 0 | 0 | 0.4 | 3.0 | 0.1 | 0 |
| ND2 (N293) - O (meta) | **4.1** | 4.9 | 16.7 | **0** | 0 | 13.5 |
| OG1 (T118) - O (para) | 1.1 | 0.8 | 18.9 | 1.1 | 1.1 | 28.6 |
| OG1 (T118) - O (meta) | 0 | 3.4 | 4.4 | 25.4 | 4.9 | 0 |
| OD1 (D113) - N (amino) | 72.6 | 47.3 | 38.6 | 75.7 | 50.9 | 55.0 |
| OD2 (D113) - N (amino) | **97.7** | 88.0 | 36.1 | **76.3** | 52.6 | 55.3 |
| OD1 (D113) - O (β-OH) | **46.6** | -[a] | 16.9 | **59.6** | - | 0 |
| OD2 (D113) - O (β-OH) | **37.0** | - | 18.0 | **60.9** | - | 0 |
| OH (Y308) - O1 ($-COO^-$) | - -[c] | 29.0 | 1.1 | - - | 0.2 | 1.5 |
| OH (Y308) - O2 ($-COO^-$) | - - | 76.6 | 8.1 | - - | 0.7 | 0.2 |
| ND2 (N312) - O (β-OH) | **47.9** | - | 21.2 | **71.7** | - | 18.5 |
| OD1 (N312) - N (amino) | **66.4** | 82.8 | 26.6 | **87.3** | 0.9 | 23.9 |
| ND2 (N312) - O1 ($-COO^-$) | - - | 0 | 0.2 | - - | 3.8 | 0 |
| ND2 (N312) - O2 ($-COO^-$) | - - | 12.9 | 3.7 | - - | 2.4 | 0 |
| OH (Y316) - O (β-OH) | 0.2 | - | 1.0 | 45.5 | - | 0 |
| OH (Y316) - N (amino) | 0 | 3.3 | 0 | 5.0 | 1.8 | 12.0 |

[a] Hydrogen bonds formed by adrenaline's conformation in the β2AR X-ray crystal structure (PDB ID: 4LDO) are highlighted in bold.
[b] β-OH group is absent in the L-DOPA ligand.
[c] $-COO^-$ group is absent in the endogenous ligand.

**Figure S21**

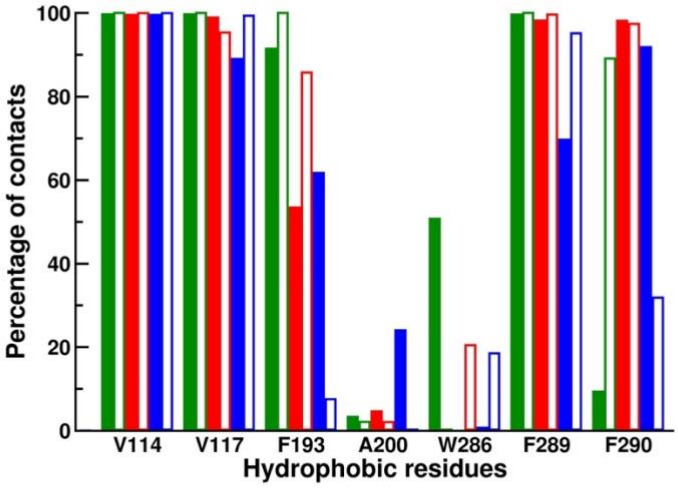


**Figure S21** Percentage of contacts of ligands with the receptor hydrophobic residues extracted from MD simulations of $\beta_2$AR-catecholamine complexes performed with CHARMM (closed bars) and OPLS (open bars) AA FFs. Adrenaline, L-DOPA and Droxidopa percentages of contacts with $\beta_2$AR hydrophobic residues are shown with green, red and blue bars, respectively. This analysis was performed over the whole 1 $\mu s$ trajectory.

**Figure S22**

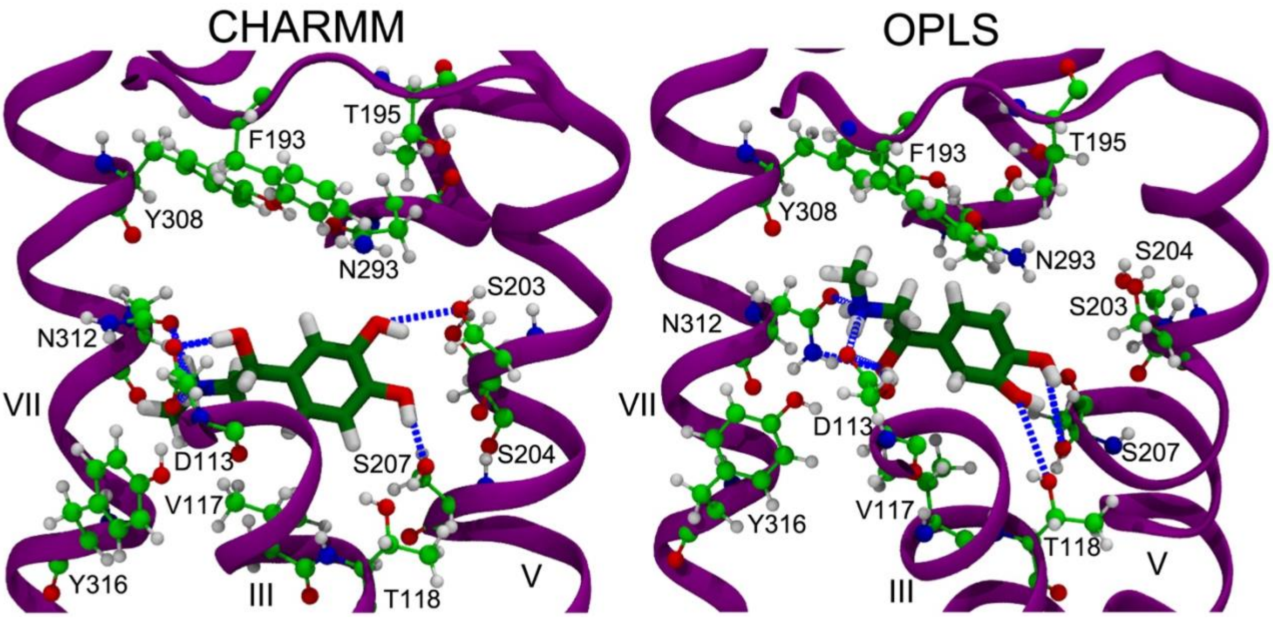


**Figure S22** Snapshots of MD simulations of $\beta_2$AR-adrenaline complexes show the hydrogen bond network of the endogenous ligand with key amino acids of the receptor binding pocket using CHARMM and OPLS AA FFs. Hydrogen bonds are highlighted with dashed blue lines. View point and color code as in Figure 5 of Biswas et al. 2021.

## Figure S23

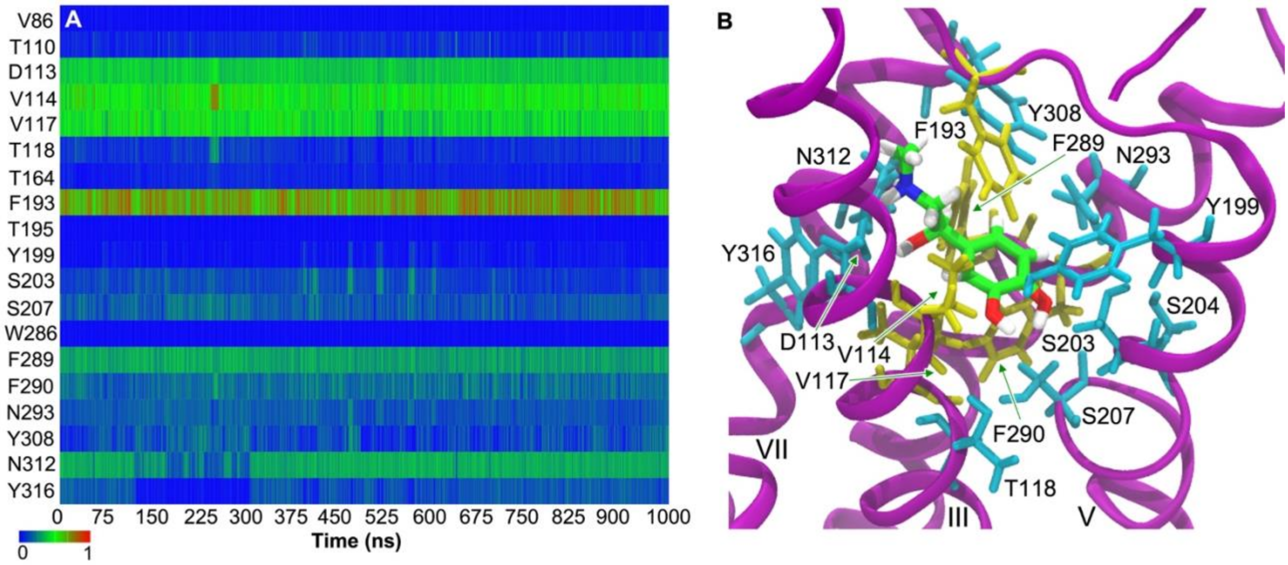


**Figure S23** (A) Normalized number of contacts of adrenaline with the receptor residues extracted from the 1 μs all atom MD simulation of the $\beta_2$AR-adrenaline complex performed with the OPLS AA FF. (B) A snapshot from the MD simulation shows $\beta_2$AR hydrophilic (cyan) and hydrophobic (yellow) residues interacting with the adrenaline ligand. For clarity, only $\beta_2$AR residues displaying the largest percentages of contacts with the ligand are shown. Sidechains of some residues are indicated by green arrows. This analysis was performed over the whole 1 $\mu s$ trajectory.

**Figure S24**

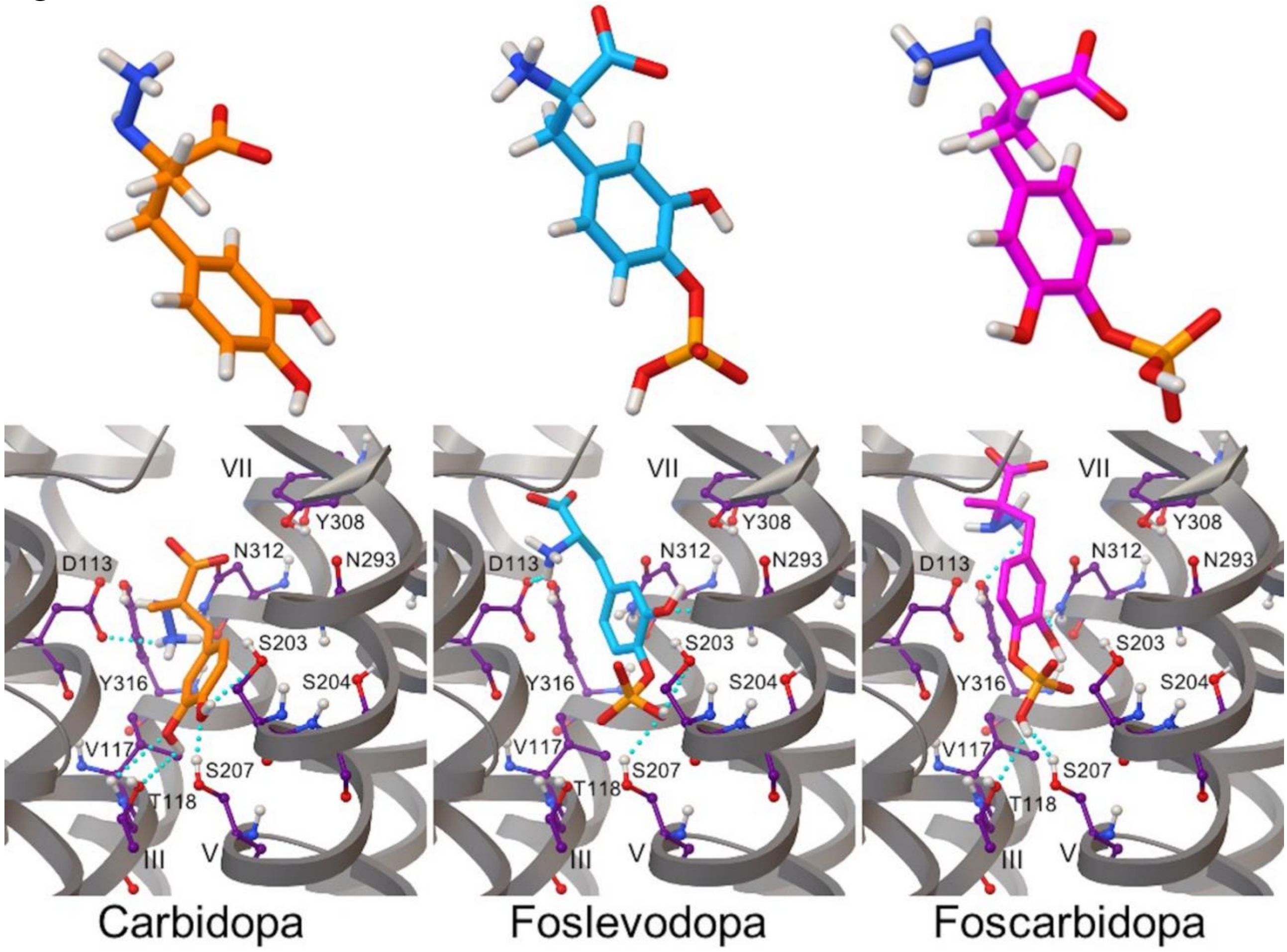


**Figure S24** (top) Chemical structures of protonated Carbidopa (orange), Foslevodopa (celestial blue) and Foscarbidopa (magenta). (bottom) Lowest energy conformations and hydrogen bond network obtained from the docking of each ligand into a rigid β$_2$AR model performed with the molecular docking software package AutoDock Vina v. 1.2.3. Color code and view point as in Figure 9.

## adrenaline.itp

```
;       OPLS AA force field parameters of Adrenaline generated with JOYCE

[ moleculetype ]
; Name            nrexcl
ALE     3

[ atoms ]
;   nr       type  resnr residue  atom   cgnr     charge       mass  typeB    chargeB
; residue   1 ALE rtp ALE  q +1.0
     1   oplsm_309     1    ALE      N      1   -0.446343    14.0027
     2   oplsm_310     1    ALE     H1      1    0.382023      1.008
     3   oplsm_310     1    ALE     H2      1    0.363936      1.008
     4   oplsm_149     1    ALE     C1      1   -0.076781     12.011
     5   oplsm_140    1    ALE     H3      1    0.137222      1.008
     6   oplsm_140    1    ALE     H4      1    0.135202      1.008
     7   oplsm_140    1    ALE     H5      1    0.135338      1.008
     8   oplsm_293     1    ALE     CA      1   -0.024055     12.011
     9   oplsm_140     1    ALE    HA1      1    0.138286      1.008
    10   oplsm_140     1    ALE    HA2      1    0.133064      1.008
    11   oplsm_149     1    ALE     CB      2    0.027731     12.011
    12   oplsm_140     1    ALE    HB1      2    0.117273      1.008
    13   oplsm_154     1    ALE     OG      3   -0.422795    15.9994
    14   oplsm_155     1    ALE     HG      3    0.347155      1.008
    15   oplsm_145     1    ALE     CG      3   -0.029545     12.011
    16   oplsm_145     1    ALE    CD1      4   -0.104846     12.011
    17   oplsm_146     1    ALE    HD1      4    0.107227      1.008
    18   oplsm_145     1    ALE    CD2      5   -0.101815     12.011
    19   oplsm_146     1    ALE    HD2      5    0.103567      1.008
    20   oplsm_166     1    ALE    CE1      6    0.073348     12.011
    21   oplsm_167     1    ALE    OE1      6   -0.394652    15.9994
    22   oplsm_168     1    ALE    HE1      6    0.340755      1.008
    23   oplsm_145     1    ALE    CE2      7   -0.107124     12.011
    24   oplsm_146     1    ALE    HE2      7    0.112182      1.008
    25   oplsm_166     1    ALE     CZ      8    0.071847     12.011
    26   oplsm_167     1    ALE     OH      8   -0.381798    15.9994
    27   oplsm_168     1    ALE     HH      8    0.363632      1.008   ; qtot 1.000033

; Stretchings
[ bonds ]
     1     2   1     0.1025     393683.477
     1     3   1     0.1028     393683.477
     1     4   1     0.1496     196521.091
     4     5   1     0.1094     328457.447
     4     6   1     0.1094     328457.447
     4     7   1     0.1093     328457.447
     1     8   1     0.1501     179667.704
     8     9   1     0.1094     323493.654
     8    10   1     0.1095     323493.654
     8    11   1     0.1524     182391.941
    11    12   1     0.1102     301357.367
    11    13   1     0.1436     199950.220
    13    14   1     0.0966     486572.973
    11    15   1     0.1512     205697.663
    15    16   1     0.1403     339484.191
    16    17   1     0.1090     332540.269
    15    18   1     0.1397     339484.191
    18    19   1     0.1091     332540.269
    16    20   1     0.1391     339484.191
```

| | | | | |
|---|---|---|---|---|
| 20 | 21 | 1 | 0.1368 | 307423.405 |
| 21 | 22 | 1 | 0.0969 | 480865.258 |
| 18 | 23 | 1 | 0.1400 | 339484.191 |
| 23 | 24 | 1 | 0.1091 | 332540.269 |
| 20 | 25 | 1 | 0.1408 | 311404.166 |
| 23 | 25 | 1 | 0.1393 | 311404.166 |
| 25 | 26 | 1 | 0.1372 | 306132.262 |
| 26 | 27 | 1 | 0.0966 | 480865.258 |

; Bendings
[ angles ]

| | | | | | |
|---|---|---|---|---|---|
| 2 | 1 | 3 | 1 | 105.80 | 366.8740 |
| 2 | 1 | 4 | 1 | 109.51 | 373.8285 |
| 2 | 1 | 8 | 1 | 109.42 | 381.2262 |
| 3 | 1 | 4 | 1 | 110.55 | 373.8285 |
| 3 | 1 | 8 | 1 | 106.93 | 381.2262 |
| 1 | 4 | 5 | 1 | 108.48 | 468.3697 |
| 1 | 4 | 6 | 1 | 108.40 | 468.3697 |
| 1 | 4 | 7 | 1 | 108.25 | 468.3697 |
| 4 | 1 | 8 | 1 | 114.27 | 472.0079 |
| 5 | 4 | 6 | 1 | 110.52 | 309.0556 |
| 5 | 4 | 7 | 1 | 110.50 | 309.0556 |
| 6 | 4 | 7 | 1 | 110.60 | 309.0556 |
| 1 | 8 | 9 | 1 | 108.31 | 451.2013 |
| 1 | 8 | 10 | 1 | 106.94 | 451.2013 |
| 1 | 8 | 11 | 1 | 109.59 | 643.5652 |
| 9 | 8 | 10 | 1 | 109.67 | 292.8912 |
| 9 | 8 | 11 | 1 | 111.50 | 292.9588 |
| 10 | 8 | 11 | 1 | 110.69 | 292.9588 |
| 8 | 11 | 12 | 1 | 109.16 | 383.0963 |
| 8 | 11 | 13 | 1 | 104.55 | 660.4204 |
| 8 | 11 | 15 | 1 | 110.97 | 474.4008 |
| 12 | 11 | 13 | 1 | 109.73 | 388.2702 |
| 12 | 11 | 15 | 1 | 108.71 | 325.9046 |
| 11 | 13 | 14 | 1 | 108.97 | 469.7023 |
| 13 | 11 | 15 | 1 | 113.61 | 557.7615 |
| 11 | 15 | 16 | 1 | 120.12 | 588.9987 |
| 11 | 15 | 18 | 1 | 120.26 | 588.9987 |
| 15 | 16 | 17 | 1 | 120.97 | 293.3534 |
| 16 | 15 | 18 | 1 | 119.62 | 495.6412 |
| 15 | 16 | 20 | 1 | 120.20 | 495.6412 |
| 17 | 16 | 20 | 1 | 118.83 | 293.3534 |
| 15 | 18 | 19 | 1 | 120.08 | 293.3534 |
| 15 | 18 | 23 | 1 | 120.43 | 495.6412 |
| 19 | 18 | 23 | 1 | 119.49 | 293.3534 |
| 16 | 20 | 21 | 1 | 119.58 | 534.1722 |
| 16 | 20 | 25 | 1 | 119.91 | 361.9651 |
| 20 | 21 | 22 | 1 | 108.56 | 522.9635 |
| 21 | 20 | 25 | 1 | 120.51 | 771.4094 |
| 18 | 23 | 24 | 1 | 120.43 | 293.3534 |
| 18 | 23 | 25 | 1 | 119.76 | 361.9651 |
| 24 | 23 | 25 | 1 | 119.80 | 320.3130 |
| 20 | 25 | 23 | 1 | 120.09 | 115.1701 |
| 20 | 25 | 26 | 1 | 115.22 | 699.2490 |
| 23 | 25 | 26 | 1 | 124.69 | 699.2490 |
| 25 | 26 | 27 | 1 | 110.64 | 448.1807 |

; Torsions - Proper dihedral angles
[ dihedrals ]
; ai aj ak al funct c0 c1 c2 c3 c4 c5

```
  3   1   4   6   1      1.11       5.300   3
  3   1   4   7   1      1.11       5.300   3
  2   1   8   9   1   270.19       5.179   1
  2   1   8   9   1  -171.66       2.193   2
  2   1   8   9   1       1.25       3.196   3
  2   1   8   10   1    270.19       5.179   1
  2   1   8   10   1   -171.66       2.193   2
  2   1   8   10   1        1.25       3.196   3
 20  25  26  27   1   -179.55       3.882   1
 20  25  26  27   1    179.73       8.726   2
  9   8  11  12   1   -162.12       2.055   1
  9   8  11  12   1      63.42       1.109   2
  9   8  11  12   1      -0.25       8.553   3
  9   8  11  12   1      -6.16       0.163   4
 12  11  15  18   1    180.84       0.004   1
 12  11  15  18   1   -152.83       5.737   2
 12  11  15  18   1    161.46       0.004   3
 12  11  15  18   1      17.84       0.217   4
 12  11  15  16   1    180.84       0.004   1
 12  11  15  16   1   -152.83       5.737   2
 12  11  15  16   1    161.46       0.004   3
 12  11  15  16   1      17.84       0.217   4
 16  20  21  22   1      -0.56       3.524   1
 16  20  21  22   1    180.05       8.762   2
  8   11  13  14   1     22.00       9.094   1
  8   11  13  14   1     70.38       1.057   2
  8   11  13  14   1     73.83       1.153   2
  8   11  13  14   1     -4.18       0.700   3

; Nonbonded terms
[ pairs ]
; 1-4 interactions
  1  12   2    0.500   -0.446    0.117    0.2875     0.14940     #  N    - HA
  1  13   2    0.500   -0.446   -0.423    0.3160     0.35564     #  N    - OH
  1  15   2    0.500   -0.446   -0.030    0.3400     0.22821     #  N    - CC
  2   5   2    0.500    0.382    0.137    0.1250     0.00000     #  H    - HA
  2   6   2    0.500    0.382    0.135    0.1250     0.00000     #  H    - HA
  2   7   2    0.500    0.382    0.135    0.1250     0.00000     #  H    - HA
  2   9   2    0.500    0.382    0.138    0.1250     0.00000     #  H    - HA
  2  10   2    0.500    0.382    0.133    0.1250     0.00000     #  H    - HA
  2  11   2    0.500    0.382    0.028    0.1750     0.00000     #  H    - CB
  3   5   2    0.500    0.364    0.137    0.1250     0.00000     #  H    - HA
  3   6   2    0.500    0.364    0.135    0.1250     0.00000     #  H    - HA
  3   7   2    0.500    0.364    0.135    0.1250     0.00000     #  H    - HA
  3   9   2    0.500    0.364    0.138    0.1250     0.00000     #  H    - HA
  3  10   2    0.500    0.364    0.133    0.1250     0.00000     #  H    - HA
  3  11   2    0.500    0.364    0.028    0.1750     0.00000     #  H    - CB
  4   9   2    0.500   -0.077    0.138    0.3125     0.11741     #  C    - HA
  4  10   2    0.500   -0.077    0.133    0.3125     0.11741     #  C    - HA
  4  11   2    0.500   -0.077    0.028    0.3625     0.17415     #  C    - CB
  5   8   2    0.500    0.137   -0.024    0.3000     0.09309     #  HA   - CA
  6   8   2    0.500    0.135   -0.024    0.3000     0.09309     #  HA   - CA
  7   8   2    0.500    0.135   -0.024    0.3000     0.09309     #  HA   - CA
  8  14   2    0.500   -0.024    0.347    0.1750     0.00000     #  CA   - HO
  8  16   2    0.500   -0.024   -0.105    0.3525     0.14219     #  CA   - CC
  8  18   2    0.500   -0.024   -0.102    0.3525     0.14219     #  CA   - CC
  9  12   2    0.500    0.138    0.117    0.2500     0.06276     #  HA   - HA
  9  13   2    0.500    0.138   -0.423    0.2785     0.14940     #  HA   - OH
  9  15   2    0.500    0.138   -0.030    0.3025     0.09587     #  HA   - CC
 10  12   2    0.500    0.133    0.117    0.2500     0.06276     #  HA   - HA
```

| | | | | | | | | |
|---|---|---|---|---|---|---|---|---|
| 10 | 13 | 2 | 0.500 | 0.133 | -0.423 | 0.2785 | 0.14940 | # HA - OH |
| 10 | 15 | 2 | 0.500 | 0.133 | -0.030 | 0.3025 | 0.09587 | # HA - CC |
| 11 | 17 | 2 | 0.500 | 0.028 | 0.107 | 0.2960 | 0.09309 | # CB - HC |
| 11 | 19 | 2 | 0.500 | 0.028 | 0.104 | 0.2960 | 0.09309 | # CB - HC |
| 11 | 20 | 2 | 0.500 | 0.028 | 0.073 | 0.3525 | 0.14219 | # CB - CC |
| 11 | 23 | 2 | 0.500 | 0.028 | -0.107 | 0.3525 | 0.14219 | # CB - CC |
| 12 | 14 | 2 | 0.500 | 0.117 | 0.347 | 0.1250 | 0.00000 | # HA - HO |
| 12 | 16 | 2 | 0.500 | 0.117 | -0.105 | 0.3025 | 0.09587 | # HA - CC |
| 12 | 18 | 2 | 0.500 | 0.117 | -0.102 | 0.3025 | 0.09587 | # HA - CC |
| 13 | 16 | 2 | 0.500 | -0.423 | -0.105 | 0.3310 | 0.22821 | # OH - CC |
| 13 | 18 | 2 | 0.500 | -0.423 | -0.102 | 0.3310 | 0.22821 | # OH - CC |
| 14 | 15 | 2 | 0.500 | 0.347 | -0.030 | 0.1775 | 0.00000 | # HO - CC |
| 15 | 21 | 2 | 0.500 | -0.030 | -0.395 | 0.3310 | 0.22821 | # CC - OH |
| 15 | 24 | 2 | 0.500 | -0.030 | 0.112 | 0.2985 | 0.09587 | # CC - HC |
| 15 | 25 | 2 | 0.500 | -0.030 | 0.072 | 0.3550 | 0.14644 | # CC - CZ |
| 16 | 19 | 2 | 0.500 | -0.105 | 0.104 | 0.2985 | 0.09587 | # CC - HC |
| 16 | 22 | 2 | 0.500 | -0.105 | 0.341 | 0.1775 | 0.00000 | # CC - HH |
| 16 | 23 | 2 | 0.500 | -0.105 | -0.107 | 0.3550 | 0.14644 | # CC - CC |
| 16 | 26 | 2 | 0.500 | -0.105 | -0.382 | 0.3310 | 0.22821 | # CC - OH |
| 17 | 18 | 2 | 0.500 | 0.107 | -0.102 | 0.2985 | 0.09587 | # HC - CC |
| 17 | 21 | 2 | 0.500 | 0.107 | -0.395 | 0.2745 | 0.14940 | # HC - OH |
| 17 | 25 | 2 | 0.500 | 0.107 | 0.072 | 0.2985 | 0.09587 | # HC - CZ |
| 18 | 20 | 2 | 0.500 | -0.102 | 0.073 | 0.3550 | 0.14644 | # CC - CC |
| 18 | 26 | 2 | 0.500 | -0.102 | -0.382 | 0.3310 | 0.22821 | # CC - OH |
| 19 | 24 | 2 | 0.500 | 0.104 | 0.112 | 0.2420 | 0.06276 | # HC - HC |
| 19 | 25 | 2 | 0.500 | 0.104 | 0.072 | 0.2985 | 0.09587 | # HC - CZ |
| 20 | 24 | 2 | 0.500 | 0.073 | 0.112 | 0.2985 | 0.09587 | # CC - HC |
| 20 | 27 | 2 | 0.500 | 0.073 | 0.364 | 0.1775 | 0.00000 | # CC - HH |
| 21 | 23 | 2 | 0.500 | -0.395 | -0.107 | 0.3310 | 0.22821 | # OH - CC |
| 21 | 26 | 2 | 0.500 | -0.395 | -0.382 | 0.3070 | 0.35564 | # OH - OH |
| 22 | 25 | 2 | 0.500 | 0.341 | 0.072 | 0.1775 | 0.00000 | # HH - CZ |
| 23 | 27 | 2 | 0.500 | -0.107 | 0.364 | 0.1775 | 0.00000 | # CC - HH |
| 24 | 26 | 2 | 0.500 | 0.112 | -0.382 | 0.2745 | 0.14940 | # HC - OH |
| 1 | 14 | 2 | 0.500 | -0.446 | 0.347 | 0.1625 | 0.00000 | # N - HO |
| 1 | 16 | 2 | 0.500 | -0.446 | -0.105 | 0.3400 | 0.45642 | # N - CC |
| 1 | 17 | 2 | 0.500 | -0.446 | 0.107 | 0.2835 | 0.29880 | # N - HC |
| 1 | 18 | 2 | 0.500 | -0.446 | -0.102 | 0.3400 | 0.45642 | # N - CC |
| 1 | 19 | 2 | 0.500 | -0.446 | 0.104 | 0.2835 | 0.29880 | # N - HC |
| 1 | 20 | 2 | 0.500 | -0.446 | 0.073 | 0.3400 | 0.45642 | # N - CC |
| 1 | 21 | 2 | 0.500 | -0.446 | -0.395 | 0.3160 | 0.71128 | # N - OH |
| 1 | 22 | 2 | 0.500 | -0.446 | 0.341 | 0.1625 | 0.00000 | # N - HH |
| 1 | 23 | 2 | 0.500 | -0.446 | -0.107 | 0.3400 | 0.45642 | # N - CC |
| 1 | 24 | 2 | 0.500 | -0.446 | 0.112 | 0.2835 | 0.29880 | # N - HC |
| 1 | 25 | 2 | 0.500 | -0.446 | 0.072 | 0.3400 | 0.45642 | # N - CZ |
| 1 | 26 | 2 | 0.500 | -0.446 | -0.382 | 0.3160 | 0.71128 | # N - OH |
| 1 | 27 | 2 | 0.500 | -0.446 | 0.364 | 0.1625 | 0.00000 | # N - HH |
| 2 | 12 | 2 | 0.500 | 0.382 | 0.117 | 0.1250 | 0.00000 | # H - HA |
| 2 | 13 | 2 | 0.500 | 0.382 | -0.423 | 0.1535 | 0.00000 | # H - OH |
| 2 | 14 | 2 | 0.500 | 0.382 | 0.347 | 0.0000 | 0.00000 | # H - HO |
| 2 | 15 | 2 | 0.500 | 0.382 | -0.030 | 0.1775 | 0.00000 | # H - CC |
| 2 | 16 | 2 | 0.500 | 0.382 | -0.105 | 0.1775 | 0.00000 | # H - CC |
| 2 | 17 | 2 | 0.500 | 0.382 | 0.107 | 0.1210 | 0.00000 | # H - HC |
| 2 | 18 | 2 | 0.500 | 0.382 | -0.102 | 0.1775 | 0.00000 | # H - CC |
| 2 | 19 | 2 | 0.500 | 0.382 | 0.104 | 0.1210 | 0.00000 | # H - HC |
| 2 | 20 | 2 | 0.500 | 0.382 | 0.073 | 0.1775 | 0.00000 | # H - CC |
| 2 | 21 | 2 | 0.500 | 0.382 | -0.395 | 0.1535 | 0.00000 | # H - OH |
| 2 | 22 | 2 | 0.500 | 0.382 | 0.341 | 0.0000 | 0.00000 | # H - HH |
| 2 | 23 | 2 | 0.500 | 0.382 | -0.107 | 0.1775 | 0.00000 | # H - CC |
| 2 | 24 | 2 | 0.500 | 0.382 | 0.112 | 0.1210 | 0.00000 | # H - HC |
| 2 | 25 | 2 | 0.500 | 0.382 | 0.072 | 0.1775 | 0.00000 | # H - CZ |

| | | | | | | | | | | |
|---|---|---|---|---|---|---|---|---|---|---|
| 2 | 26 | 2 | 0.500 | 0.382 | -0.382 | 0.1535 | 0.00000 | # | H | - OH |
| 2 | 27 | 2 | 0.500 | 0.382 | 0.364 | 0.0000 | 0.00000 | # | H | - HH |
| 3 | 12 | 2 | 0.500 | 0.364 | 0.117 | 0.1250 | 0.00000 | # | H | - HA |
| 3 | 13 | 2 | 0.500 | 0.364 | -0.423 | 0.1535 | 0.00000 | # | H | - OH |
| 3 | 14 | 2 | 0.500 | 0.364 | 0.347 | 0.0000 | 0.00000 | # | H | - HO |
| 3 | 15 | 2 | 0.500 | 0.364 | -0.030 | 0.1775 | 0.00000 | # | H | - CC |
| 3 | 16 | 2 | 0.500 | 0.364 | -0.105 | 0.1775 | 0.00000 | # | H | - CC |
| 3 | 17 | 2 | 0.500 | 0.364 | 0.107 | 0.1210 | 0.00000 | # | H | - HC |
| 3 | 18 | 2 | 0.500 | 0.364 | -0.102 | 0.1775 | 0.00000 | # | H | - CC |
| 3 | 19 | 2 | 0.500 | 0.364 | 0.104 | 0.1210 | 0.00000 | # | H | - HC |
| 3 | 20 | 2 | 0.500 | 0.364 | 0.073 | 0.1775 | 0.00000 | # | H | - CC |
| 3 | 21 | 2 | 0.500 | 0.364 | -0.395 | 0.1535 | 0.00000 | # | H | - OH |
| 3 | 22 | 2 | 0.500 | 0.364 | 0.341 | 0.0000 | 0.00000 | # | H | - HH |
| 3 | 23 | 2 | 0.500 | 0.364 | -0.107 | 0.1775 | 0.00000 | # | H | - CC |
| 3 | 24 | 2 | 0.500 | 0.364 | 0.112 | 0.1210 | 0.00000 | # | H | - HC |
| 3 | 25 | 2 | 0.500 | 0.364 | 0.072 | 0.1775 | 0.00000 | # | H | - CZ |
| 3 | 26 | 2 | 0.500 | 0.364 | -0.382 | 0.1535 | 0.00000 | # | H | - OH |
| 3 | 27 | 2 | 0.500 | 0.364 | 0.364 | 0.0000 | 0.00000 | # | H | - HH |
| 4 | 12 | 2 | 0.500 | -0.077 | 0.117 | 0.3125 | 0.23483 | # | C | - HA |
| 4 | 13 | 2 | 0.500 | -0.077 | -0.423 | 0.3410 | 0.55900 | # | C | - OH |
| 4 | 14 | 2 | 0.500 | -0.077 | 0.347 | 0.1875 | 0.00000 | # | C | - HO |
| 4 | 15 | 2 | 0.500 | -0.077 | -0.030 | 0.3650 | 0.35870 | # | C | - CC |
| 4 | 16 | 2 | 0.500 | -0.077 | -0.105 | 0.3650 | 0.35870 | # | C | - CC |
| 4 | 17 | 2 | 0.500 | -0.077 | 0.107 | 0.3085 | 0.23483 | # | C | - HC |
| 4 | 18 | 2 | 0.500 | -0.077 | -0.102 | 0.3650 | 0.35870 | # | C | - CC |
| 4 | 19 | 2 | 0.500 | -0.077 | 0.104 | 0.3085 | 0.23483 | # | C | - HC |
| 4 | 20 | 2 | 0.500 | -0.077 | 0.073 | 0.3650 | 0.35870 | # | C | - CC |
| 4 | 21 | 2 | 0.500 | -0.077 | -0.395 | 0.3410 | 0.55900 | # | C | - OH |
| 4 | 22 | 2 | 0.500 | -0.077 | 0.341 | 0.1875 | 0.00000 | # | C | - HH |
| 4 | 23 | 2 | 0.500 | -0.077 | -0.107 | 0.3650 | 0.35870 | # | C | - CC |
| 4 | 24 | 2 | 0.500 | -0.077 | 0.112 | 0.3085 | 0.23483 | # | C | - HC |
| 4 | 25 | 2 | 0.500 | -0.077 | 0.072 | 0.3650 | 0.35870 | # | C | - CZ |
| 4 | 26 | 2 | 0.500 | -0.077 | -0.382 | 0.3410 | 0.55900 | # | C | - OH |
| 4 | 27 | 2 | 0.500 | -0.077 | 0.364 | 0.1875 | 0.00000 | # | C | - HH |
| 5 | 9 | 2 | 0.500 | 0.137 | 0.138 | 0.2500 | 0.12552 | # | HA | - HA |
| 5 | 10 | 2 | 0.500 | 0.137 | 0.133 | 0.2500 | 0.12552 | # | HA | - HA |
| 5 | 11 | 2 | 0.500 | 0.137 | 0.028 | 0.3000 | 0.18618 | # | HA | - CB |
| 5 | 12 | 2 | 0.500 | 0.137 | 0.117 | 0.2500 | 0.12552 | # | HA | - HA |
| 5 | 13 | 2 | 0.500 | 0.137 | -0.423 | 0.2785 | 0.29880 | # | HA | - OH |
| 5 | 14 | 2 | 0.500 | 0.137 | 0.347 | 0.1250 | 0.00000 | # | HA | - HO |
| 5 | 15 | 2 | 0.500 | 0.137 | -0.030 | 0.3025 | 0.19173 | # | HA | - CC |
| 5 | 16 | 2 | 0.500 | 0.137 | -0.105 | 0.3025 | 0.19173 | # | HA | - CC |
| 5 | 17 | 2 | 0.500 | 0.137 | 0.107 | 0.2460 | 0.12552 | # | HA | - HC |
| 5 | 18 | 2 | 0.500 | 0.137 | -0.102 | 0.3025 | 0.19173 | # | HA | - CC |
| 5 | 19 | 2 | 0.500 | 0.137 | 0.104 | 0.2460 | 0.12552 | # | HA | - HC |
| 5 | 20 | 2 | 0.500 | 0.137 | 0.073 | 0.3025 | 0.19173 | # | HA | - CC |
| 5 | 21 | 2 | 0.500 | 0.137 | -0.395 | 0.2785 | 0.29880 | # | HA | - OH |
| 5 | 22 | 2 | 0.500 | 0.137 | 0.341 | 0.1250 | 0.00000 | # | HA | - HH |
| 5 | 23 | 2 | 0.500 | 0.137 | -0.107 | 0.3025 | 0.19173 | # | HA | - CC |
| 5 | 24 | 2 | 0.500 | 0.137 | 0.112 | 0.2460 | 0.12552 | # | HA | - HC |
| 5 | 25 | 2 | 0.500 | 0.137 | 0.072 | 0.3025 | 0.19173 | # | HA | - CZ |
| 5 | 26 | 2 | 0.500 | 0.137 | -0.382 | 0.2785 | 0.29880 | # | HA | - OH |
| 5 | 27 | 2 | 0.500 | 0.137 | 0.364 | 0.1250 | 0.00000 | # | HA | - HH |
| 6 | 9 | 2 | 0.500 | 0.135 | 0.138 | 0.2500 | 0.12552 | # | HA | - HA |
| 6 | 10 | 2 | 0.500 | 0.135 | 0.133 | 0.2500 | 0.12552 | # | HA | - HA |
| 6 | 11 | 2 | 0.500 | 0.135 | 0.028 | 0.3000 | 0.18618 | # | HA | - CB |
| 6 | 12 | 2 | 0.500 | 0.135 | 0.117 | 0.2500 | 0.12552 | # | HA | - HA |
| 6 | 13 | 2 | 0.500 | 0.135 | -0.423 | 0.2785 | 0.29880 | # | HA | - OH |
| 6 | 14 | 2 | 0.500 | 0.135 | 0.347 | 0.1250 | 0.00000 | # | HA | - HO |
| 6 | 15 | 2 | 0.500 | 0.135 | -0.030 | 0.3025 | 0.19173 | # | HA | - CC |

| | | | | | | | | |
|---|---|---|---|---|---|---|---|---|
| 6 | 16 | 2 | 0.500 | 0.135 | -0.105 | 0.3025 | 0.19173 | # HA - CC |
| 6 | 17 | 2 | 0.500 | 0.135 | 0.107 | 0.2460 | 0.12552 | # HA - HC |
| 6 | 18 | 2 | 0.500 | 0.135 | -0.102 | 0.3025 | 0.19173 | # HA - CC |
| 6 | 19 | 2 | 0.500 | 0.135 | 0.104 | 0.2460 | 0.12552 | # HA - HC |
| 6 | 20 | 2 | 0.500 | 0.135 | 0.073 | 0.3025 | 0.19173 | # HA - CC |
| 6 | 21 | 2 | 0.500 | 0.135 | -0.395 | 0.2785 | 0.29880 | # HA - OH |
| 6 | 22 | 2 | 0.500 | 0.135 | 0.341 | 0.1250 | 0.00000 | # HA - HH |
| 6 | 23 | 2 | 0.500 | 0.135 | -0.107 | 0.3025 | 0.19173 | # HA - CC |
| 6 | 24 | 2 | 0.500 | 0.135 | 0.112 | 0.2460 | 0.12552 | # HA - HC |
| 6 | 25 | 2 | 0.500 | 0.135 | 0.072 | 0.3025 | 0.19173 | # HA - CZ |
| 6 | 26 | 2 | 0.500 | 0.135 | -0.382 | 0.2785 | 0.29880 | # HA - OH |
| 6 | 27 | 2 | 0.500 | 0.135 | 0.364 | 0.1250 | 0.00000 | # HA - HH |
| 7 | 9 | 2 | 0.500 | 0.135 | 0.138 | 0.2500 | 0.12552 | # HA - HA |
| 7 | 10 | 2 | 0.500 | 0.135 | 0.133 | 0.2500 | 0.12552 | # HA - HA |
| 7 | 11 | 2 | 0.500 | 0.135 | 0.028 | 0.3000 | 0.18618 | # HA - CB |
| 7 | 12 | 2 | 0.500 | 0.135 | 0.117 | 0.2500 | 0.12552 | # HA - HA |
| 7 | 13 | 2 | 0.500 | 0.135 | -0.423 | 0.2785 | 0.29880 | # HA - OH |
| 7 | 14 | 2 | 0.500 | 0.135 | 0.347 | 0.1250 | 0.00000 | # HA - HO |
| 7 | 15 | 2 | 0.500 | 0.135 | -0.030 | 0.3025 | 0.19173 | # HA - CC |
| 7 | 16 | 2 | 0.500 | 0.135 | -0.105 | 0.3025 | 0.19173 | # HA - CC |
| 7 | 17 | 2 | 0.500 | 0.135 | 0.107 | 0.2460 | 0.12552 | # HA - HC |
| 7 | 18 | 2 | 0.500 | 0.135 | -0.102 | 0.3025 | 0.19173 | # HA - CC |
| 7 | 19 | 2 | 0.500 | 0.135 | 0.104 | 0.2460 | 0.12552 | # HA - HC |
| 7 | 20 | 2 | 0.500 | 0.135 | 0.073 | 0.3025 | 0.19173 | # HA - CC |
| 7 | 21 | 2 | 0.500 | 0.135 | -0.395 | 0.2785 | 0.29880 | # HA - OH |
| 7 | 22 | 2 | 0.500 | 0.135 | 0.341 | 0.1250 | 0.00000 | # HA - HH |
| 7 | 23 | 2 | 0.500 | 0.135 | -0.107 | 0.3025 | 0.19173 | # HA - CC |
| 7 | 24 | 2 | 0.500 | 0.135 | 0.112 | 0.2460 | 0.12552 | # HA - HC |
| 7 | 25 | 2 | 0.500 | 0.135 | 0.072 | 0.3025 | 0.19173 | # HA - CZ |
| 7 | 26 | 2 | 0.500 | 0.135 | -0.382 | 0.2785 | 0.29880 | # HA - OH |
| 7 | 27 | 2 | 0.500 | 0.135 | 0.364 | 0.1250 | 0.00000 | # HA - HH |
| 8 | 17 | 2 | 0.500 | -0.024 | 0.107 | 0.2960 | 0.18618 | # CA - HC |
| 8 | 19 | 2 | 0.500 | -0.024 | 0.104 | 0.2960 | 0.18618 | # CA - HC |
| 8 | 20 | 2 | 0.500 | -0.024 | 0.073 | 0.3525 | 0.28439 | # CA - CC |
| 8 | 21 | 2 | 0.500 | -0.024 | -0.395 | 0.3285 | 0.44319 | # CA - OH |
| 8 | 22 | 2 | 0.500 | -0.024 | 0.341 | 0.1750 | 0.00000 | # CA - HH |
| 8 | 23 | 2 | 0.500 | -0.024 | -0.107 | 0.3525 | 0.28439 | # CA - CC |
| 8 | 24 | 2 | 0.500 | -0.024 | 0.112 | 0.2960 | 0.18618 | # CA - HC |
| 8 | 25 | 2 | 0.500 | -0.024 | 0.072 | 0.3525 | 0.28439 | # CA - CZ |
| 8 | 26 | 2 | 0.500 | -0.024 | -0.382 | 0.3285 | 0.44319 | # CA - OH |
| 8 | 27 | 2 | 0.500 | -0.024 | 0.364 | 0.1750 | 0.00000 | # CA - HH |
| 9 | 14 | 2 | 0.500 | 0.138 | 0.347 | 0.1250 | 0.00000 | # HA - HO |
| 9 | 16 | 2 | 0.500 | 0.138 | -0.105 | 0.3025 | 0.19173 | # HA - CC |
| 9 | 17 | 2 | 0.500 | 0.138 | 0.107 | 0.2460 | 0.12552 | # HA - HC |
| 9 | 18 | 2 | 0.500 | 0.138 | -0.102 | 0.3025 | 0.19173 | # HA - CC |
| 9 | 19 | 2 | 0.500 | 0.138 | 0.104 | 0.2460 | 0.12552 | # HA - HC |
| 9 | 20 | 2 | 0.500 | 0.138 | 0.073 | 0.3025 | 0.19173 | # HA - CC |
| 9 | 21 | 2 | 0.500 | 0.138 | -0.395 | 0.2785 | 0.29880 | # HA - OH |
| 9 | 22 | 2 | 0.500 | 0.138 | 0.341 | 0.1250 | 0.00000 | # HA - HH |
| 9 | 23 | 2 | 0.500 | 0.138 | -0.107 | 0.3025 | 0.19173 | # HA - CC |
| 9 | 24 | 2 | 0.500 | 0.138 | 0.112 | 0.2460 | 0.12552 | # HA - HC |
| 9 | 25 | 2 | 0.500 | 0.138 | 0.072 | 0.3025 | 0.19173 | # HA - CZ |
| 9 | 26 | 2 | 0.500 | 0.138 | -0.382 | 0.2785 | 0.29880 | # HA - OH |
| 9 | 27 | 2 | 0.500 | 0.138 | 0.364 | 0.1250 | 0.00000 | # HA - HH |
| 10 | 14 | 2 | 0.500 | 0.133 | 0.347 | 0.1250 | 0.00000 | # HA - HO |
| 10 | 16 | 2 | 0.500 | 0.133 | -0.105 | 0.3025 | 0.19173 | # HA - CC |
| 10 | 17 | 2 | 0.500 | 0.133 | 0.107 | 0.2460 | 0.12552 | # HA - HC |
| 10 | 18 | 2 | 0.500 | 0.133 | -0.102 | 0.3025 | 0.19173 | # HA - CC |
| 10 | 19 | 2 | 0.500 | 0.133 | 0.104 | 0.2460 | 0.12552 | # HA - HC |
| 10 | 20 | 2 | 0.500 | 0.133 | 0.073 | 0.3025 | 0.19173 | # HA - CC |

| | | | | | | | | | | |
|---|---|---|---|---|---|---|---|---|---|---|
| 10 | 21 | 2 | 0.500 | 0.133 | -0.395 | 0.2785 | 0.29880 | # | HA | - OH |
| 10 | 22 | 2 | 0.500 | 0.133 | 0.341 | 0.1250 | 0.00000 | # | HA | - HH |
| 10 | 23 | 2 | 0.500 | 0.133 | -0.107 | 0.3025 | 0.19173 | # | HA | - CC |
| 10 | 24 | 2 | 0.500 | 0.133 | 0.112 | 0.2460 | 0.12552 | # | HA | - HC |
| 10 | 25 | 2 | 0.500 | 0.133 | 0.072 | 0.3025 | 0.19173 | # | HA | - CZ |
| 10 | 26 | 2 | 0.500 | 0.133 | -0.382 | 0.2785 | 0.29880 | # | HA | - OH |
| 10 | 27 | 2 | 0.500 | 0.133 | 0.364 | 0.1250 | 0.00000 | # | HA | - HH |
| 11 | 21 | 2 | 0.500 | 0.028 | -0.395 | 0.3285 | 0.44319 | # | CB | - OH |
| 11 | 22 | 2 | 0.500 | 0.028 | 0.341 | 0.1750 | 0.00000 | # | CB | - HH |
| 11 | 24 | 2 | 0.500 | 0.028 | 0.112 | 0.2960 | 0.18618 | # | CB | - HC |
| 11 | 25 | 2 | 0.500 | 0.028 | 0.072 | 0.3525 | 0.28439 | # | CB | - CZ |
| 11 | 26 | 2 | 0.500 | 0.028 | -0.382 | 0.3285 | 0.44319 | # | CB | - OH |
| 11 | 27 | 2 | 0.500 | 0.028 | 0.364 | 0.1750 | 0.00000 | # | CB | - HH |
| 12 | 17 | 2 | 0.500 | 0.117 | 0.107 | 0.2460 | 0.12552 | # | HA | - HC |
| 12 | 19 | 2 | 0.500 | 0.117 | 0.104 | 0.2460 | 0.12552 | # | HA | - HC |
| 12 | 20 | 2 | 0.500 | 0.117 | 0.073 | 0.3025 | 0.19173 | # | HA | - CC |
| 12 | 21 | 2 | 0.500 | 0.117 | -0.395 | 0.2785 | 0.29880 | # | HA | - OH |
| 12 | 22 | 2 | 0.500 | 0.117 | 0.341 | 0.1250 | 0.00000 | # | HA | - HH |
| 12 | 23 | 2 | 0.500 | 0.117 | -0.107 | 0.3025 | 0.19173 | # | HA | - CC |
| 12 | 24 | 2 | 0.500 | 0.117 | 0.112 | 0.2460 | 0.12552 | # | HA | - HC |
| 12 | 25 | 2 | 0.500 | 0.117 | 0.072 | 0.3025 | 0.19173 | # | HA | - CZ |
| 12 | 26 | 2 | 0.500 | 0.117 | -0.382 | 0.2785 | 0.29880 | # | HA | - OH |
| 12 | 27 | 2 | 0.500 | 0.117 | 0.364 | 0.1250 | 0.00000 | # | HA | - HH |
| 13 | 17 | 2 | 0.500 | -0.423 | 0.107 | 0.2745 | 0.29880 | # | OH | - HC |
| 13 | 19 | 2 | 0.500 | -0.423 | 0.104 | 0.2745 | 0.29880 | # | OH | - HC |
| 13 | 20 | 2 | 0.500 | -0.423 | 0.073 | 0.3310 | 0.45642 | # | OH | - CC |
| 13 | 21 | 2 | 0.500 | -0.423 | -0.395 | 0.3070 | 0.71128 | # | OH | - OH |
| 13 | 22 | 2 | 0.500 | -0.423 | 0.341 | 0.1535 | 0.00000 | # | OH | - HH |
| 13 | 23 | 2 | 0.500 | -0.423 | -0.107 | 0.3310 | 0.45642 | # | OH | - CC |
| 13 | 24 | 2 | 0.500 | -0.423 | 0.112 | 0.2745 | 0.29880 | # | OH | - HC |
| 13 | 25 | 2 | 0.500 | -0.423 | 0.072 | 0.3310 | 0.45642 | # | OH | - CZ |
| 13 | 26 | 2 | 0.500 | -0.423 | -0.382 | 0.3070 | 0.71128 | # | OH | - OH |
| 13 | 27 | 2 | 0.500 | -0.423 | 0.364 | 0.1535 | 0.00000 | # | OH | - HH |
| 14 | 16 | 2 | 0.500 | 0.347 | -0.105 | 0.1775 | 0.00000 | # | HO | - CC |
| 14 | 17 | 2 | 0.500 | 0.347 | 0.107 | 0.1210 | 0.00000 | # | HO | - HC |
| 14 | 18 | 2 | 0.500 | 0.347 | -0.102 | 0.1775 | 0.00000 | # | HO | - CC |
| 14 | 19 | 2 | 0.500 | 0.347 | 0.104 | 0.1210 | 0.00000 | # | HO | - HC |
| 14 | 20 | 2 | 0.500 | 0.347 | 0.073 | 0.1775 | 0.00000 | # | HO | - CC |
| 14 | 21 | 2 | 0.500 | 0.347 | -0.395 | 0.1535 | 0.00000 | # | HO | - OH |
| 14 | 22 | 2 | 0.500 | 0.347 | 0.341 | 0.0000 | 0.00000 | # | HO | - HH |
| 14 | 23 | 2 | 0.500 | 0.347 | -0.107 | 0.1775 | 0.00000 | # | HO | - CC |
| 14 | 24 | 2 | 0.500 | 0.347 | 0.112 | 0.1210 | 0.00000 | # | HO | - HC |
| 14 | 25 | 2 | 0.500 | 0.347 | 0.072 | 0.1775 | 0.00000 | # | HO | - CZ |
| 14 | 26 | 2 | 0.500 | 0.347 | -0.382 | 0.1535 | 0.00000 | # | HO | - OH |
| 14 | 27 | 2 | 0.500 | 0.347 | 0.364 | 0.0000 | 0.00000 | # | HO | - HH |
| 15 | 22 | 2 | 0.500 | -0.030 | 0.341 | 0.1775 | 0.00000 | # | CC | - HH |
| 15 | 26 | 2 | 0.500 | -0.030 | -0.382 | 0.3310 | 0.45642 | # | CC | - OH |
| 15 | 27 | 2 | 0.500 | -0.030 | 0.364 | 0.1775 | 0.00000 | # | CC | - HH |
| 16 | 24 | 2 | 0.500 | -0.105 | 0.112 | 0.2985 | 0.19173 | # | CC | - HC |
| 16 | 27 | 2 | 0.500 | -0.105 | 0.364 | 0.1775 | 0.00000 | # | CC | - HH |
| 17 | 19 | 2 | 0.500 | 0.107 | 0.104 | 0.2420 | 0.12552 | # | HC | - HC |
| 17 | 22 | 2 | 0.500 | 0.107 | 0.341 | 0.1210 | 0.00000 | # | HC | - HH |
| 17 | 23 | 2 | 0.500 | 0.107 | -0.107 | 0.2985 | 0.19173 | # | HC | - CC |
| 17 | 24 | 2 | 0.500 | 0.107 | 0.112 | 0.2420 | 0.12552 | # | HC | - HC |
| 17 | 26 | 2 | 0.500 | 0.107 | -0.382 | 0.2745 | 0.29880 | # | HC | - OH |
| 17 | 27 | 2 | 0.500 | 0.107 | 0.364 | 0.1210 | 0.00000 | # | HC | - HH |
| 18 | 21 | 2 | 0.500 | -0.102 | -0.395 | 0.3310 | 0.45642 | # | CC | - OH |
| 18 | 22 | 2 | 0.500 | -0.102 | 0.341 | 0.1775 | 0.00000 | # | CC | - HH |
| 18 | 27 | 2 | 0.500 | -0.102 | 0.364 | 0.1775 | 0.00000 | # | CC | - HH |
| 19 | 20 | 2 | 0.500 | 0.104 | 0.073 | 0.2985 | 0.19173 | # | HC | - CC |

```
  19  21   2    0.500    0.104   -0.395    0.2745     0.29880     #  HC   - OH
  19  22   2    0.500    0.104    0.341    0.1210     0.00000     #  HC   - HH
  19  26   2    0.500    0.104   -0.382    0.2745     0.29880     #  HC   - OH
  19  27   2    0.500    0.104    0.364    0.1210     0.00000     #  HC   - HH
  21  24   2    0.500   -0.395    0.112    0.2745     0.29880     #  OH   - HC
  21  27   2    0.500   -0.395    0.364    0.1535     0.00000     #  OH   - HH
  22  23   2    0.500    0.341   -0.107    0.1775     0.00000     #  HH   - CC
  22  24   2    0.500    0.341    0.112    0.1210     0.00000     #  HH   - HC
  22  26   2    0.500    0.341   -0.382    0.1535     0.00000     #  HH   - OH
  22  27   2    0.500    0.341    0.364    0.0000     0.00000     #  HH   - HH
  24  27   2    0.500    0.112    0.364    0.1210     0.00000     #  HC   - HH

; Exclusions from default nonbonded
[ exclusions ]
; ai     aj
    1     2
    1     3
    1     4
    1     5
    1     6
    1     7
    1     8
    1     9
    1    10
    1    11
    1    12
    1    13
    1    14
    1    15
    1    16
    1    17
    1    18
    1    19
    1    20
    1    21
    1    22
    1    23
    1    24
    1    25
    1    26
    1    27
    2     3
    2     4
    2     5
    2     6
    2     7
    2     8
    2     9
    2    10
    2    11
    2    12
    2    13
    2    14
    2    15
    2    16
    2    17
    2    18
    2    19
    2    20
    2    21
```

| | |
|---|---|
| 2 | 22 |
| 2 | 23 |
| 2 | 24 |
| 2 | 25 |
| 2 | 26 |
| 2 | 27 |
| 3 | 4 |
| 3 | 5 |
| 3 | 6 |
| 3 | 7 |
| 3 | 8 |
| 3 | 9 |
| 3 | 10 |
| 3 | 11 |
| 3 | 12 |
| 3 | 13 |
| 3 | 14 |
| 3 | 15 |
| 3 | 16 |
| 3 | 17 |
| 3 | 18 |
| 3 | 19 |
| 3 | 20 |
| 3 | 21 |
| 3 | 22 |
| 3 | 23 |
| 3 | 24 |
| 3 | 25 |
| 3 | 26 |
| 3 | 27 |
| 4 | 5 |
| 4 | 6 |
| 4 | 7 |
| 4 | 8 |
| 4 | 9 |
| 4 | 10 |
| 4 | 11 |
| 4 | 12 |
| 4 | 13 |
| 4 | 14 |
| 4 | 15 |
| 4 | 16 |
| 4 | 17 |
| 4 | 18 |
| 4 | 19 |
| 4 | 20 |
| 4 | 21 |
| 4 | 22 |
| 4 | 23 |
| 4 | 24 |
| 4 | 25 |
| 4 | 26 |
| 4 | 27 |
| 5 | 6 |
| 5 | 7 |
| 5 | 8 |
| 5 | 9 |
| 5 | 10 |
| 5 | 11 |
| 5 | 12 |

| | |
|---|---|
| 5 | 13 |
| 5 | 14 |
| 5 | 15 |
| 5 | 16 |
| 5 | 17 |
| 5 | 18 |
| 5 | 19 |
| 5 | 20 |
| 5 | 21 |
| 5 | 22 |
| 5 | 23 |
| 5 | 24 |
| 5 | 25 |
| 5 | 26 |
| 5 | 27 |
| 6 | 7 |
| 6 | 8 |
| 6 | 9 |
| 6 | 10 |
| 6 | 11 |
| 6 | 12 |
| 6 | 13 |
| 6 | 14 |
| 6 | 15 |
| 6 | 16 |
| 6 | 17 |
| 6 | 18 |
| 6 | 19 |
| 6 | 20 |
| 6 | 21 |
| 6 | 22 |
| 6 | 23 |
| 6 | 24 |
| 6 | 25 |
| 6 | 26 |
| 6 | 27 |
| 7 | 8 |
| 7 | 9 |
| 7 | 10 |
| 7 | 11 |
| 7 | 12 |
| 7 | 13 |
| 7 | 14 |
| 7 | 15 |
| 7 | 16 |
| 7 | 17 |
| 7 | 18 |
| 7 | 19 |
| 7 | 20 |
| 7 | 21 |
| 7 | 22 |
| 7 | 23 |
| 7 | 24 |
| 7 | 25 |
| 7 | 26 |
| 7 | 27 |
| 8 | 9 |
| 8 | 10 |
| 8 | 11 |
| 8 | 12 |

| | |
|---|---|
| 8 | 13 |
| 8 | 14 |
| 8 | 15 |
| 8 | 16 |
| 8 | 17 |
| 8 | 18 |
| 8 | 19 |
| 8 | 20 |
| 8 | 21 |
| 8 | 22 |
| 8 | 23 |
| 8 | 24 |
| 8 | 25 |
| 8 | 26 |
| 8 | 27 |
| 9 | 10 |
| 9 | 11 |
| 9 | 12 |
| 9 | 13 |
| 9 | 14 |
| 9 | 15 |
| 9 | 16 |
| 9 | 17 |
| 9 | 18 |
| 9 | 19 |
| 9 | 20 |
| 9 | 21 |
| 9 | 22 |
| 9 | 23 |
| 9 | 24 |
| 9 | 25 |
| 9 | 26 |
| 9 | 27 |
| 10 | 11 |
| 10 | 12 |
| 10 | 13 |
| 10 | 14 |
| 10 | 15 |
| 10 | 16 |
| 10 | 17 |
| 10 | 18 |
| 10 | 19 |
| 10 | 20 |
| 10 | 21 |
| 10 | 22 |
| 10 | 23 |
| 10 | 24 |
| 10 | 25 |
| 10 | 26 |
| 10 | 27 |
| 11 | 12 |
| 11 | 13 |
| 11 | 14 |
| 11 | 15 |
| 11 | 16 |
| 11 | 17 |
| 11 | 18 |
| 11 | 19 |
| 11 | 20 |
| 11 | 21 |

| | |
|---|---|
| 11 | 22 |
| 11 | 23 |
| 11 | 24 |
| 11 | 25 |
| 11 | 26 |
| 11 | 27 |
| 12 | 13 |
| 12 | 14 |
| 12 | 15 |
| 12 | 16 |
| 12 | 17 |
| 12 | 18 |
| 12 | 19 |
| 12 | 20 |
| 12 | 21 |
| 12 | 22 |
| 12 | 23 |
| 12 | 24 |
| 12 | 25 |
| 12 | 26 |
| 12 | 27 |
| 13 | 14 |
| 13 | 15 |
| 13 | 16 |
| 13 | 17 |
| 13 | 18 |
| 13 | 19 |
| 13 | 20 |
| 13 | 21 |
| 13 | 22 |
| 13 | 23 |
| 13 | 24 |
| 13 | 25 |
| 13 | 26 |
| 13 | 27 |
| 14 | 15 |
| 14 | 16 |
| 14 | 17 |
| 14 | 18 |
| 14 | 19 |
| 14 | 20 |
| 14 | 21 |
| 14 | 22 |
| 14 | 23 |
| 14 | 24 |
| 14 | 25 |
| 14 | 26 |
| 14 | 27 |
| 15 | 16 |
| 15 | 17 |
| 15 | 18 |
| 15 | 19 |
| 15 | 20 |
| 15 | 21 |
| 15 | 22 |
| 15 | 23 |
| 15 | 24 |
| 15 | 25 |
| 15 | 26 |
| 15 | 27 |

| | |
|---|---|
| 16 | 17 |
| 16 | 18 |
| 16 | 19 |
| 16 | 20 |
| 16 | 21 |
| 16 | 22 |
| 16 | 23 |
| 16 | 24 |
| 16 | 25 |
| 16 | 26 |
| 16 | 27 |
| 17 | 18 |
| 17 | 19 |
| 17 | 20 |
| 17 | 21 |
| 17 | 22 |
| 17 | 23 |
| 17 | 24 |
| 17 | 25 |
| 17 | 26 |
| 17 | 27 |
| 18 | 19 |
| 18 | 20 |
| 18 | 21 |
| 18 | 22 |
| 18 | 23 |
| 18 | 24 |
| 18 | 25 |
| 18 | 26 |
| 18 | 27 |
| 19 | 20 |
| 19 | 21 |
| 19 | 22 |
| 19 | 23 |
| 19 | 24 |
| 19 | 25 |
| 19 | 26 |
| 19 | 27 |
| 20 | 21 |
| 20 | 22 |
| 20 | 23 |
| 20 | 24 |
| 20 | 25 |
| 20 | 26 |
| 20 | 27 |
| 21 | 22 |
| 21 | 23 |
| 21 | 24 |
| 21 | 25 |
| 21 | 26 |
| 21 | 27 |
| 22 | 23 |
| 22 | 24 |
| 22 | 25 |
| 22 | 26 |
| 22 | 27 |
| 23 | 24 |
| 23 | 25 |
| 23 | 26 |
| 23 | 27 |

| | |
|---|---|
| 24 | 25 |
| 24 | 26 |
| 24 | 27 |
| 25 | 26 |
| 25 | 27 |
| 26 | 27 |

**dopa.itp**

```
; OPLS AA force field parameters of L-DOPA generated with JOYCE

[ moleculetype ]
; Name            nrexcl
DAH     3

[ atoms ]
;   nr       type  resnr residue  atom   cgnr     charge       mass  typeB    chargeB
; residue   1 DAH rtp DAH  q +0.0
     1   oplsm_287      1    DAH      N      1    -0.570913    14.0027
     2   oplsm_290      1    DAH     H1      1     0.344381      1.008
     3   oplsm_290      1    DAH     H2      1     0.325205      1.008
     4   oplsm_290      1    DAH     H3      1     0.377593      1.008
     5   oplsm_293      1    DAH     CA      1     0.028407     12.011
     6   oplsm_140      1    DAH     HA      1     0.132597      1.008
     7   oplsm_149      1    DAH     CB      2    -0.140334     12.011
     8   oplsm_140      1    DAH    HB1      2     0.102677      1.008
     9   oplsm_140      1    DAH    HB2      2     0.101894      1.008
    10   oplsm_145      1    DAH     CG      2    -0.026831     12.011
    11   oplsm_145      1    DAH    CD1      3    -0.110132     12.011
    12   oplsm_146      1    DAH    HD1      3     0.100316      1.008
    13   oplsm_145      1    DAH    CD2      4    -0.108542     12.011
    14   oplsm_146      1    DAH    HD2      4     0.102219      1.008
    15   oplsm_166      1    DAH    CE1      5     0.072835     12.011
    16   oplsm_167      1    DAH    OE1      5    -0.394908    15.9994
    17   oplsm_168      1    DAH    HE1      5     0.339949      1.008
    18   oplsm_145      1    DAH    CE2      6    -0.108072     12.011
    19   oplsm_146      1    DAH    HE2      6     0.111219      1.008
    20   oplsm_166      1    DAH     CZ      7     0.068134     12.011
    21   oplsm_167      1    DAH     OH      7    -0.384411    15.9994
    22   oplsm_168      1    DAH     HH      7     0.362376      1.008
    23   oplsm_271      1    DAH      C      8     0.188364     12.011
    24   oplsm_272      1    DAH     O1      8    -0.450134    15.9994
    25   oplsm_272      1    DAH     O2      8    -0.463910    15.9994   ; qtot 0.000021

; Stretchings
[ bonds ]
     1     2   1     0.1026     368489.034
     1     3   1     0.1054     368489.034
     1     4   1     0.1020     368489.034
     1     5   1     0.1506     180341.536
     5     6   1     0.1095     318058.259
     5     7   1     0.1536     180195.043
     7     8   1     0.1098     314597.457
     7     9   1     0.1096     314597.457
     7    10   1     0.1512     214106.063
    10    11   1     0.1405     332239.630
    11    12   1     0.1090     331723.534
    10    13   1     0.1401     332239.630
    13    14   1     0.1091     331723.534
    11    15   1     0.1391     332239.630
    15    16   1     0.1368     305030.302
    16    17   1     0.0969     480979.109
    13    18   1     0.1402     332239.630
    18    19   1     0.1091     331723.534
    15    20   1     0.1408     309763.805
    18    20   1     0.1391     309763.805
    20    21   1     0.1374     305476.385
```

| | | | | |
|---|---|---|---|---|
| 21 | 22 | 1 | 0.0966 | 480979.109 |
| 5 | 23 | 1 | 0.1560 | 136630.345 |
| 23 | 24 | 1 | 0.1269 | 478155.015 |
| 23 | 25 | 1 | 0.1247 | 478155.015 |

; Bendings
[ angles ]

| | | | | | |
|---|---|---|---|---|---|
| 2 | 1 | 3 | 1 | 108.76 | 338.0992 |
| 2 | 1 | 4 | 1 | 108.90 | 338.0992 |
| 2 | 1 | 5 | 1 | 111.79 | 354.8638 |
| 3 | 1 | 4 | 1 | 111.69 | 338.0992 |
| 3 | 1 | 5 | 1 | 101.35 | 354.8638 |
| 4 | 1 | 5 | 1 | 114.10 | 354.8638 |
| 1 | 5 | 6 | 1 | 107.34 | 467.6459 |
| 1 | 5 | 7 | 1 | 111.77 | 482.7422 |
| 1 | 5 | 23 | 1 | 105.54 | 528.7628 |
| 6 | 5 | 7 | 1 | 109.70 | 396.4863 |
| 6 | 5 | 23 | 1 | 107.64 | 316.9483 |
| 5 | 7 | 8 | 1 | 109.02 | 342.6356 |
| 5 | 7 | 9 | 1 | 106.28 | 342.6356 |
| 5 | 7 | 10 | 1 | 112.82 | 622.4218 |
| 7 | 5 | 23 | 1 | 114.51 | 523.3779 |
| 8 | 7 | 9 | 1 | 107.62 | 295.6076 |
| 8 | 7 | 10 | 1 | 109.80 | 348.8275 |
| 9 | 7 | 10 | 1 | 111.11 | 348.8275 |
| 7 | 10 | 11 | 1 | 120.42 | 495.9408 |
| 7 | 10 | 13 | 1 | 120.67 | 495.9408 |
| 10 | 11 | 12 | 1 | 120.47 | 295.1435 |
| 11 | 10 | 13 | 1 | 118.84 | 539.0730 |
| 10 | 11 | 15 | 1 | 120.58 | 539.0730 |
| 12 | 11 | 15 | 1 | 118.94 | 295.1435 |
| 10 | 13 | 14 | 1 | 119.99 | 295.1435 |
| 10 | 13 | 18 | 1 | 120.86 | 539.0730 |
| 14 | 13 | 18 | 1 | 119.14 | 295.1435 |
| 11 | 15 | 16 | 1 | 119.53 | 574.6185 |
| 11 | 15 | 20 | 1 | 120.05 | 379.8506 |
| 15 | 16 | 17 | 1 | 108.53 | 518.3672 |
| 16 | 15 | 20 | 1 | 120.42 | 796.0599 |
| 13 | 18 | 19 | 1 | 120.37 | 295.1435 |
| 13 | 18 | 20 | 1 | 119.76 | 379.8506 |
| 19 | 18 | 20 | 1 | 119.86 | 327.6687 |
| 15 | 20 | 18 | 1 | 119.90 | 99.2215 |
| 15 | 20 | 21 | 1 | 115.28 | 699.1805 |
| 18 | 20 | 21 | 1 | 124.82 | 699.1805 |
| 20 | 21 | 22 | 1 | 110.60 | 449.2292 |
| 5 | 23 | 24 | 1 | 114.70 | 372.9536 |
| 5 | 23 | 25 | 1 | 116.62 | 372.9536 |
| 24 | 23 | 25 | 1 | 128.67 | 683.7864 |

; Torsions
[ dihedrals ]

| | | | | | | | |
|---|---|---|---|---|---|---|---|
| 6 | 5 | 23 | 24 | 1 | 320.79 | 9.731 | 2 |
| 6 | 5 | 23 | 25 | 1 | 318.43 | 9.731 | 2 |
| 2 | 1 | 5 | 6 | 1 | 127.76 | 2.482 | 3 |
| 3 | 1 | 5 | 6 | 1 | 140.68 | 2.482 | 3 |
| 18 | 20 | 21 | 22 | 1 | 0.00 | 3.251 | 1 |
| 18 | 20 | 21 | 22 | 1 | 179.99 | 8.441 | 2 |
| 6 | 5 | 7 | 8 | 1 | -193.73 | 5.697 | 1 |
| 6 | 5 | 7 | 8 | 1 | -94.39 | 1.592 | 2 |
| 6 | 5 | 7 | 8 | 1 | -14.05 | 6.757 | 3 |

```
    6   5   7   8   1      8.32        0.158   4
    8   7  10  13   1    242.40        0.135   1
    8   7  10  13   1    199.06        8.060   2
    8   7  10  13   1    252.02        0.038   3
    8   7  10  13   1    -62.10        2.030   4
    8   7  10  11   1    242.40        0.135   1
    8   7  10  11   1    199.06        8.060   2
    8   7  10  11   1    252.02        0.038   3
    8   7  10  11   1    -62.10        2.030   4
   11  15  16  17   1    359.82        3.153   1
   11  15  16  17   1    179.64        9.114   2

; Nonbonded terms
[ pairs ]
; 1-4 interactions
    1   8   2   0.500  -0.571   0.103   0.2875   0.14940   #  N    - HA
    1   9   2   0.500  -0.571   0.102   0.2875   0.14940   #  N    - HA
    1  10   2   0.500  -0.571  -0.027   0.3400   0.22821   #  N    - CC
    1  24   2   0.500  -0.571  -0.450   0.3105   0.39527   #  N    - O
    1  25   2   0.500  -0.571  -0.464   0.3105   0.39527   #  N    - O
    2   6   2   0.500   0.344   0.133   0.1250   0.00000   #  H    - HA
    2   7   2   0.500   0.344  -0.140   0.1750   0.00000   #  H    - CB
    2  23   2   0.500   0.344   0.188   0.1875   0.00000   #  H    - C
    3   6   2   0.500   0.325   0.133   0.1250   0.00000   #  H    - HA
    3   7   2   0.500   0.325  -0.140   0.1750   0.00000   #  H    - CB
    3  23   2   0.500   0.325   0.188   0.1875   0.00000   #  H    - C
    4   6   2   0.500   0.378   0.133   0.1250   0.00000   #  H    - HA
    4   7   2   0.500   0.378  -0.140   0.1750   0.00000   #  H    - CB
    4  23   2   0.500   0.378   0.188   0.1875   0.00000   #  H    - C
    5  11   2   0.500   0.028  -0.110   0.3525   0.14219   #  CA   - CC
    5  13   2   0.500   0.028  -0.109   0.3525   0.14219   #  CA   - CC
    6   8   2   0.500   0.133   0.103   0.2500   0.06276   #  HA   - HA
    6   9   2   0.500   0.133   0.102   0.2500   0.06276   #  HA   - HA
    6  10   2   0.500   0.133  -0.027   0.3025   0.09587   #  HA   - CC
    6  24   2   0.500   0.133  -0.450   0.2730   0.16605   #  HA   - O
    6  25   2   0.500   0.133  -0.464   0.2730   0.16605   #  HA   - O
    7  12   2   0.500  -0.140   0.100   0.2960   0.09309   #  CB   - HC
    7  14   2   0.500  -0.140   0.102   0.2960   0.09309   #  CB   - HC
    7  15   2   0.500  -0.140   0.073   0.3525   0.14219   #  CB   - CC
    7  18   2   0.500  -0.140  -0.108   0.3525   0.14219   #  CB   - CC
    7  24   2   0.500  -0.140  -0.450   0.3230   0.24629   #  CB   - O
    7  25   2   0.500  -0.140  -0.464   0.3230   0.24629   #  CB   - O
    8  11   2   0.500   0.103  -0.110   0.3025   0.09587   #  HA   - CC
    8  13   2   0.500   0.103  -0.109   0.3025   0.09587   #  HA   - CC
    8  23   2   0.500   0.103   0.188   0.3125   0.11741   #  HA   - C
    9  11   2   0.500   0.102  -0.110   0.3025   0.09587   #  HA   - CC
    9  13   2   0.500   0.102  -0.109   0.3025   0.09587   #  HA   - CC
    9  23   2   0.500   0.102   0.188   0.3125   0.11741   #  HA   - C
   10  16   2   0.500  -0.027  -0.395   0.3310   0.22821   #  CC   - OH
   10  19   2   0.500  -0.027   0.111   0.2985   0.09587   #  CC   - HC
   10  20   2   0.500  -0.027   0.068   0.3550   0.14644   #  CC   - CZ
   10  23   2   0.500  -0.027   0.188   0.3650   0.17935   #  CC   - C
   11  14   2   0.500  -0.110   0.102   0.2985   0.09587   #  CC   - HC
   11  17   2   0.500  -0.110   0.340   0.1775   0.00000   #  CC   - HH
   11  18   2   0.500  -0.110  -0.108   0.3550   0.14644   #  CC   - CC
   11  21   2   0.500  -0.110  -0.384   0.3310   0.22821   #  CC   - OH
   12  13   2   0.500   0.100  -0.109   0.2985   0.09587   #  HC   - CC
   12  16   2   0.500   0.100  -0.395   0.2745   0.14940   #  HC   - OH
   12  20   2   0.500   0.100   0.068   0.2985   0.09587   #  HC   - CZ
   13  15   2   0.500  -0.109   0.073   0.3550   0.14644   #  CC   - CC
```

| | | | | | | | | |
|---|---|---|---|---|---|---|---|---|
| 13 | 21 | 2 | 0.500 | -0.109 | -0.384 | 0.3310 | 0.22821 | # CC - OH |
| 14 | 19 | 2 | 0.500 | 0.102 | 0.111 | 0.2420 | 0.06276 | # HC - HC |
| 14 | 20 | 2 | 0.500 | 0.102 | 0.068 | 0.2985 | 0.09587 | # HC - CZ |
| 15 | 19 | 2 | 0.500 | 0.073 | 0.111 | 0.2985 | 0.09587 | # CC - HC |
| 15 | 22 | 2 | 0.500 | 0.073 | 0.362 | 0.1775 | 0.00000 | # CC - HH |
| 16 | 18 | 2 | 0.500 | -0.395 | -0.108 | 0.3310 | 0.22821 | # OH - CC |
| 16 | 21 | 2 | 0.500 | -0.395 | -0.384 | 0.3070 | 0.35564 | # OH - OH |
| 17 | 20 | 2 | 0.500 | 0.340 | 0.068 | 0.1775 | 0.00000 | # HH - CZ |
| 18 | 22 | 2 | 0.500 | -0.108 | 0.362 | 0.1775 | 0.00000 | # CC - HH |
| 19 | 21 | 2 | 0.500 | 0.111 | -0.384 | 0.2745 | 0.14940 | # HC - OH |
| 1 | 11 | 2 | 0.500 | -0.571 | -0.110 | 0.3400 | 0.45642 | # N - CC |
| 1 | 12 | 2 | 0.500 | -0.571 | 0.100 | 0.2835 | 0.29880 | # N - HC |
| 1 | 13 | 2 | 0.500 | -0.571 | -0.109 | 0.3400 | 0.45642 | # N - CC |
| 1 | 14 | 2 | 0.500 | -0.571 | 0.102 | 0.2835 | 0.29880 | # N - HC |
| 1 | 15 | 2 | 0.500 | -0.571 | 0.073 | 0.3400 | 0.45642 | # N - CC |
| 1 | 16 | 2 | 0.500 | -0.571 | -0.395 | 0.3160 | 0.71128 | # N - OH |
| 1 | 17 | 2 | 0.500 | -0.571 | 0.340 | 0.1625 | 0.00000 | # N - HH |
| 1 | 18 | 2 | 0.500 | -0.571 | -0.108 | 0.3400 | 0.45642 | # N - CC |
| 1 | 19 | 2 | 0.500 | -0.571 | 0.111 | 0.2835 | 0.29880 | # N - HC |
| 1 | 20 | 2 | 0.500 | -0.571 | 0.068 | 0.3400 | 0.45642 | # N - CZ |
| 1 | 21 | 2 | 0.500 | -0.571 | -0.384 | 0.3160 | 0.71128 | # N - OH |
| 1 | 22 | 2 | 0.500 | -0.571 | 0.362 | 0.1625 | 0.00000 | # N - HH |
| 2 | 8 | 2 | 0.500 | 0.344 | 0.103 | 0.1250 | 0.00000 | # H - HA |
| 2 | 9 | 2 | 0.500 | 0.344 | 0.102 | 0.1250 | 0.00000 | # H - HA |
| 2 | 10 | 2 | 0.500 | 0.344 | -0.027 | 0.1775 | 0.00000 | # H - CC |
| 2 | 11 | 2 | 0.500 | 0.344 | -0.110 | 0.1775 | 0.00000 | # H - CC |
| 2 | 12 | 2 | 0.500 | 0.344 | 0.100 | 0.1210 | 0.00000 | # H - HC |
| 2 | 13 | 2 | 0.500 | 0.344 | -0.109 | 0.1775 | 0.00000 | # H - CC |
| 2 | 14 | 2 | 0.500 | 0.344 | 0.102 | 0.1210 | 0.00000 | # H - HC |
| 2 | 15 | 2 | 0.500 | 0.344 | 0.073 | 0.1775 | 0.00000 | # H - CC |
| 2 | 16 | 2 | 0.500 | 0.344 | -0.395 | 0.1535 | 0.00000 | # H - OH |
| 2 | 17 | 2 | 0.500 | 0.344 | 0.340 | 0.0000 | 0.00000 | # H - HH |
| 2 | 18 | 2 | 0.500 | 0.344 | -0.108 | 0.1775 | 0.00000 | # H - CC |
| 2 | 19 | 2 | 0.500 | 0.344 | 0.111 | 0.1210 | 0.00000 | # H - HC |
| 2 | 20 | 2 | 0.500 | 0.344 | 0.068 | 0.1775 | 0.00000 | # H - CZ |
| 2 | 21 | 2 | 0.500 | 0.344 | -0.384 | 0.1535 | 0.00000 | # H - OH |
| 2 | 22 | 2 | 0.500 | 0.344 | 0.362 | 0.0000 | 0.00000 | # H - HH |
| 2 | 24 | 2 | 0.500 | 0.344 | -0.450 | 0.1480 | 0.00000 | # H - O |
| 2 | 25 | 2 | 0.500 | 0.344 | -0.464 | 0.1480 | 0.00000 | # H - O |
| 3 | 8 | 2 | 0.500 | 0.325 | 0.103 | 0.1250 | 0.00000 | # H - HA |
| 3 | 9 | 2 | 0.500 | 0.325 | 0.102 | 0.1250 | 0.00000 | # H - HA |
| 3 | 10 | 2 | 0.500 | 0.325 | -0.027 | 0.1775 | 0.00000 | # H - CC |
| 3 | 11 | 2 | 0.500 | 0.325 | -0.110 | 0.1775 | 0.00000 | # H - CC |
| 3 | 12 | 2 | 0.500 | 0.325 | 0.100 | 0.1210 | 0.00000 | # H - HC |
| 3 | 13 | 2 | 0.500 | 0.325 | -0.109 | 0.1775 | 0.00000 | # H - CC |
| 3 | 14 | 2 | 0.500 | 0.325 | 0.102 | 0.1210 | 0.00000 | # H - HC |
| 3 | 15 | 2 | 0.500 | 0.325 | 0.073 | 0.1775 | 0.00000 | # H - CC |
| 3 | 16 | 2 | 0.500 | 0.325 | -0.395 | 0.1535 | 0.00000 | # H - OH |
| 3 | 17 | 2 | 0.500 | 0.325 | 0.340 | 0.0000 | 0.00000 | # H - HH |
| 3 | 18 | 2 | 0.500 | 0.325 | -0.108 | 0.1775 | 0.00000 | # H - CC |
| 3 | 19 | 2 | 0.500 | 0.325 | 0.111 | 0.1210 | 0.00000 | # H - HC |
| 3 | 20 | 2 | 0.500 | 0.325 | 0.068 | 0.1775 | 0.00000 | # H - CZ |
| 3 | 21 | 2 | 0.500 | 0.325 | -0.384 | 0.1535 | 0.00000 | # H - OH |
| 3 | 22 | 2 | 0.500 | 0.325 | 0.362 | 0.0000 | 0.00000 | # H - HH |
| 3 | 24 | 2 | 0.500 | 0.325 | -0.450 | 0.1480 | 0.00000 | # H - O |
| 3 | 25 | 2 | 0.500 | 0.325 | -0.464 | 0.1480 | 0.00000 | # H - O |
| 4 | 8 | 2 | 0.500 | 0.378 | 0.103 | 0.1250 | 0.00000 | # H - HA |
| 4 | 9 | 2 | 0.500 | 0.378 | 0.102 | 0.1250 | 0.00000 | # H - HA |
| 4 | 10 | 2 | 0.500 | 0.378 | -0.027 | 0.1775 | 0.00000 | # H - CC |
| 4 | 11 | 2 | 0.500 | 0.378 | -0.110 | 0.1775 | 0.00000 | # H - CC |

| | | | | | | | | |
|---|---|---|---|---|---|---|---|---|
| 4 | 12 | 2 | 0.500 | 0.378 | 0.100 | 0.1210 | 0.00000 | # H - HC |
| 4 | 13 | 2 | 0.500 | 0.378 | -0.109 | 0.1775 | 0.00000 | # H - CC |
| 4 | 14 | 2 | 0.500 | 0.378 | 0.102 | 0.1210 | 0.00000 | # H - HC |
| 4 | 15 | 2 | 0.500 | 0.378 | 0.073 | 0.1775 | 0.00000 | # H - CC |
| 4 | 16 | 2 | 0.500 | 0.378 | -0.395 | 0.1535 | 0.00000 | # H - OH |
| 4 | 17 | 2 | 0.500 | 0.378 | 0.340 | 0.0000 | 0.00000 | # H - HH |
| 4 | 18 | 2 | 0.500 | 0.378 | -0.108 | 0.1775 | 0.00000 | # H - CC |
| 4 | 19 | 2 | 0.500 | 0.378 | 0.111 | 0.1210 | 0.00000 | # H - HC |
| 4 | 20 | 2 | 0.500 | 0.378 | 0.068 | 0.1775 | 0.00000 | # H - CZ |
| 4 | 21 | 2 | 0.500 | 0.378 | -0.384 | 0.1535 | 0.00000 | # H - OH |
| 4 | 22 | 2 | 0.500 | 0.378 | 0.362 | 0.0000 | 0.00000 | # H - HH |
| 4 | 24 | 2 | 0.500 | 0.378 | -0.450 | 0.1480 | 0.00000 | # H - O |
| 4 | 25 | 2 | 0.500 | 0.378 | -0.464 | 0.1480 | 0.00000 | # H - O |
| 5 | 12 | 2 | 0.500 | 0.028 | 0.100 | 0.2960 | 0.18618 | # CA - HC |
| 5 | 14 | 2 | 0.500 | 0.028 | 0.102 | 0.2960 | 0.18618 | # CA - HC |
| 5 | 15 | 2 | 0.500 | 0.028 | 0.073 | 0.3525 | 0.28439 | # CA - CC |
| 5 | 16 | 2 | 0.500 | 0.028 | -0.395 | 0.3285 | 0.44319 | # CA - OH |
| 5 | 17 | 2 | 0.500 | 0.028 | 0.340 | 0.1750 | 0.00000 | # CA - HH |
| 5 | 18 | 2 | 0.500 | 0.028 | -0.108 | 0.3525 | 0.28439 | # CA - CC |
| 5 | 19 | 2 | 0.500 | 0.028 | 0.111 | 0.2960 | 0.18618 | # CA - HC |
| 5 | 20 | 2 | 0.500 | 0.028 | 0.068 | 0.3525 | 0.28439 | # CA - CZ |
| 5 | 21 | 2 | 0.500 | 0.028 | -0.384 | 0.3285 | 0.44319 | # CA - OH |
| 5 | 22 | 2 | 0.500 | 0.028 | 0.362 | 0.1750 | 0.00000 | # CA - HH |
| 6 | 11 | 2 | 0.500 | 0.133 | -0.110 | 0.3025 | 0.19173 | # HA - CC |
| 6 | 12 | 2 | 0.500 | 0.133 | 0.100 | 0.2460 | 0.12552 | # HA - HC |
| 6 | 13 | 2 | 0.500 | 0.133 | -0.109 | 0.3025 | 0.19173 | # HA - CC |
| 6 | 14 | 2 | 0.500 | 0.133 | 0.102 | 0.2460 | 0.12552 | # HA - HC |
| 6 | 15 | 2 | 0.500 | 0.133 | 0.073 | 0.3025 | 0.19173 | # HA - CC |
| 6 | 16 | 2 | 0.500 | 0.133 | -0.395 | 0.2785 | 0.29880 | # HA - OH |
| 6 | 17 | 2 | 0.500 | 0.133 | 0.340 | 0.1250 | 0.00000 | # HA - HH |
| 6 | 18 | 2 | 0.500 | 0.133 | -0.108 | 0.3025 | 0.19173 | # HA - CC |
| 6 | 19 | 2 | 0.500 | 0.133 | 0.111 | 0.2460 | 0.12552 | # HA - HC |
| 6 | 20 | 2 | 0.500 | 0.133 | 0.068 | 0.3025 | 0.19173 | # HA - CZ |
| 6 | 21 | 2 | 0.500 | 0.133 | -0.384 | 0.2785 | 0.29880 | # HA - OH |
| 6 | 22 | 2 | 0.500 | 0.133 | 0.362 | 0.1250 | 0.00000 | # HA - HH |
| 7 | 16 | 2 | 0.500 | -0.140 | -0.395 | 0.3285 | 0.44319 | # CB - OH |
| 7 | 17 | 2 | 0.500 | -0.140 | 0.340 | 0.1750 | 0.00000 | # CB - HH |
| 7 | 19 | 2 | 0.500 | -0.140 | 0.111 | 0.2960 | 0.18618 | # CB - HC |
| 7 | 20 | 2 | 0.500 | -0.140 | 0.068 | 0.3525 | 0.28439 | # CB - CZ |
| 7 | 21 | 2 | 0.500 | -0.140 | -0.384 | 0.3285 | 0.44319 | # CB - OH |
| 7 | 22 | 2 | 0.500 | -0.140 | 0.362 | 0.1750 | 0.00000 | # CB - HH |
| 8 | 12 | 2 | 0.500 | 0.103 | 0.100 | 0.2460 | 0.12552 | # HA - HC |
| 8 | 14 | 2 | 0.500 | 0.103 | 0.102 | 0.2460 | 0.12552 | # HA - HC |
| 8 | 15 | 2 | 0.500 | 0.103 | 0.073 | 0.3025 | 0.19173 | # HA - CC |
| 8 | 16 | 2 | 0.500 | 0.103 | -0.395 | 0.2785 | 0.29880 | # HA - OH |
| 8 | 17 | 2 | 0.500 | 0.103 | 0.340 | 0.1250 | 0.00000 | # HA - HH |
| 8 | 18 | 2 | 0.500 | 0.103 | -0.108 | 0.3025 | 0.19173 | # HA - CC |
| 8 | 19 | 2 | 0.500 | 0.103 | 0.111 | 0.2460 | 0.12552 | # HA - HC |
| 8 | 20 | 2 | 0.500 | 0.103 | 0.068 | 0.3025 | 0.19173 | # HA - CZ |
| 8 | 21 | 2 | 0.500 | 0.103 | -0.384 | 0.2785 | 0.29880 | # HA - OH |
| 8 | 22 | 2 | 0.500 | 0.103 | 0.362 | 0.1250 | 0.00000 | # HA - HH |
| 8 | 24 | 2 | 0.500 | 0.103 | -0.450 | 0.2730 | 0.33209 | # HA - O |
| 8 | 25 | 2 | 0.500 | 0.103 | -0.464 | 0.2730 | 0.33209 | # HA - O |
| 9 | 12 | 2 | 0.500 | 0.102 | 0.100 | 0.2460 | 0.12552 | # HA - HC |
| 9 | 14 | 2 | 0.500 | 0.102 | 0.102 | 0.2460 | 0.12552 | # HA - HC |
| 9 | 15 | 2 | 0.500 | 0.102 | 0.073 | 0.3025 | 0.19173 | # HA - CC |
| 9 | 16 | 2 | 0.500 | 0.102 | -0.395 | 0.2785 | 0.29880 | # HA - OH |
| 9 | 17 | 2 | 0.500 | 0.102 | 0.340 | 0.1250 | 0.00000 | # HA - HH |
| 9 | 18 | 2 | 0.500 | 0.102 | -0.108 | 0.3025 | 0.19173 | # HA - CC |
| 9 | 19 | 2 | 0.500 | 0.102 | 0.111 | 0.2460 | 0.12552 | # HA - HC |

| | | | | | | | | |
|---|---|---|---|---|---|---|---|---|
| 9 | 20 | 2 | 0.500 | 0.102 | 0.068 | 0.3025 | 0.19173 | # HA - CZ |
| 9 | 21 | 2 | 0.500 | 0.102 | -0.384 | 0.2785 | 0.29880 | # HA - OH |
| 9 | 22 | 2 | 0.500 | 0.102 | 0.362 | 0.1250 | 0.00000 | # HA - HH |
| 9 | 24 | 2 | 0.500 | 0.102 | -0.450 | 0.2730 | 0.33209 | # HA - O |
| 9 | 25 | 2 | 0.500 | 0.102 | -0.464 | 0.2730 | 0.33209 | # HA - O |
| 10 | 17 | 2 | 0.500 | -0.027 | 0.340 | 0.1775 | 0.00000 | # CC - HH |
| 10 | 21 | 2 | 0.500 | -0.027 | -0.384 | 0.3310 | 0.45642 | # CC - OH |
| 10 | 22 | 2 | 0.500 | -0.027 | 0.362 | 0.1775 | 0.00000 | # CC - HH |
| 10 | 24 | 2 | 0.500 | -0.027 | -0.450 | 0.3255 | 0.50728 | # CC - O |
| 10 | 25 | 2 | 0.500 | -0.027 | -0.464 | 0.3255 | 0.50728 | # CC - O |
| 11 | 19 | 2 | 0.500 | -0.110 | 0.111 | 0.2985 | 0.19173 | # CC - HC |
| 11 | 22 | 2 | 0.500 | -0.110 | 0.362 | 0.1775 | 0.00000 | # CC - HH |
| 11 | 23 | 2 | 0.500 | -0.110 | 0.188 | 0.3650 | 0.35870 | # CC - C |
| 11 | 24 | 2 | 0.500 | -0.110 | -0.450 | 0.3255 | 0.50728 | # CC - O |
| 11 | 25 | 2 | 0.500 | -0.110 | -0.464 | 0.3255 | 0.50728 | # CC - O |
| 12 | 14 | 2 | 0.500 | 0.100 | 0.102 | 0.2420 | 0.12552 | # HC - HC |
| 12 | 17 | 2 | 0.500 | 0.100 | 0.340 | 0.1210 | 0.00000 | # HC - HH |
| 12 | 18 | 2 | 0.500 | 0.100 | -0.108 | 0.2985 | 0.19173 | # HC - CC |
| 12 | 19 | 2 | 0.500 | 0.100 | 0.111 | 0.2420 | 0.12552 | # HC - HC |
| 12 | 21 | 2 | 0.500 | 0.100 | -0.384 | 0.2745 | 0.29880 | # HC - OH |
| 12 | 22 | 2 | 0.500 | 0.100 | 0.362 | 0.1210 | 0.00000 | # HC - HH |
| 12 | 23 | 2 | 0.500 | 0.100 | 0.188 | 0.3085 | 0.23483 | # HC - C |
| 12 | 24 | 2 | 0.500 | 0.100 | -0.450 | 0.2690 | 0.33209 | # HC - O |
| 12 | 25 | 2 | 0.500 | 0.100 | -0.464 | 0.2690 | 0.33209 | # HC - O |
| 13 | 16 | 2 | 0.500 | -0.109 | -0.395 | 0.3310 | 0.45642 | # CC - OH |
| 13 | 17 | 2 | 0.500 | -0.109 | 0.340 | 0.1775 | 0.00000 | # CC - HH |
| 13 | 22 | 2 | 0.500 | -0.109 | 0.362 | 0.1775 | 0.00000 | # CC - HH |
| 13 | 23 | 2 | 0.500 | -0.109 | 0.188 | 0.3650 | 0.35870 | # CC - C |
| 13 | 24 | 2 | 0.500 | -0.109 | -0.450 | 0.3255 | 0.50728 | # CC - O |
| 13 | 25 | 2 | 0.500 | -0.109 | -0.464 | 0.3255 | 0.50728 | # CC - O |
| 14 | 15 | 2 | 0.500 | 0.102 | 0.073 | 0.2985 | 0.19173 | # HC - CC |
| 14 | 16 | 2 | 0.500 | 0.102 | -0.395 | 0.2745 | 0.29880 | # HC - OH |
| 14 | 17 | 2 | 0.500 | 0.102 | 0.340 | 0.1210 | 0.00000 | # HC - HH |
| 14 | 21 | 2 | 0.500 | 0.102 | -0.384 | 0.2745 | 0.29880 | # HC - OH |
| 14 | 22 | 2 | 0.500 | 0.102 | 0.362 | 0.1210 | 0.00000 | # HC - HH |
| 14 | 23 | 2 | 0.500 | 0.102 | 0.188 | 0.3085 | 0.23483 | # HC - C |
| 14 | 24 | 2 | 0.500 | 0.102 | -0.450 | 0.2690 | 0.33209 | # HC - O |
| 14 | 25 | 2 | 0.500 | 0.102 | -0.464 | 0.2690 | 0.33209 | # HC - O |
| 15 | 23 | 2 | 0.500 | 0.073 | 0.188 | 0.3650 | 0.35870 | # CC - C |
| 15 | 24 | 2 | 0.500 | 0.073 | -0.450 | 0.3255 | 0.50728 | # CC - O |
| 15 | 25 | 2 | 0.500 | 0.073 | -0.464 | 0.3255 | 0.50728 | # CC - O |
| 16 | 19 | 2 | 0.500 | -0.395 | 0.111 | 0.2745 | 0.29880 | # OH - HC |
| 16 | 22 | 2 | 0.500 | -0.395 | 0.362 | 0.1535 | 0.00000 | # OH - HH |
| 16 | 23 | 2 | 0.500 | -0.395 | 0.188 | 0.3410 | 0.55900 | # OH - C |
| 16 | 24 | 2 | 0.500 | -0.395 | -0.450 | 0.3015 | 0.79054 | # OH - O |
| 16 | 25 | 2 | 0.500 | -0.395 | -0.464 | 0.3015 | 0.79054 | # OH - O |
| 17 | 18 | 2 | 0.500 | 0.340 | -0.108 | 0.1775 | 0.00000 | # HH - CC |
| 17 | 19 | 2 | 0.500 | 0.340 | 0.111 | 0.1210 | 0.00000 | # HH - HC |
| 17 | 21 | 2 | 0.500 | 0.340 | -0.384 | 0.1535 | 0.00000 | # HH - OH |
| 17 | 22 | 2 | 0.500 | 0.340 | 0.362 | 0.0000 | 0.00000 | # HH - HH |
| 17 | 23 | 2 | 0.500 | 0.340 | 0.188 | 0.1875 | 0.00000 | # HH - C |
| 17 | 24 | 2 | 0.500 | 0.340 | -0.450 | 0.1480 | 0.00000 | # HH - O |
| 17 | 25 | 2 | 0.500 | 0.340 | -0.464 | 0.1480 | 0.00000 | # HH - O |
| 18 | 23 | 2 | 0.500 | -0.108 | 0.188 | 0.3650 | 0.35870 | # CC - C |
| 18 | 24 | 2 | 0.500 | -0.108 | -0.450 | 0.3255 | 0.50728 | # CC - O |
| 18 | 25 | 2 | 0.500 | -0.108 | -0.464 | 0.3255 | 0.50728 | # CC - O |
| 19 | 22 | 2 | 0.500 | 0.111 | 0.362 | 0.1210 | 0.00000 | # HC - HH |
| 19 | 23 | 2 | 0.500 | 0.111 | 0.188 | 0.3085 | 0.23483 | # HC - C |
| 19 | 24 | 2 | 0.500 | 0.111 | -0.450 | 0.2690 | 0.33209 | # HC - O |
| 19 | 25 | 2 | 0.500 | 0.111 | -0.464 | 0.2690 | 0.33209 | # HC - O |

```
  20  23   2    0.500    0.068    0.188    0.3650     0.35870     #  CZ   - C
  20  24   2    0.500    0.068   -0.450    0.3255     0.50728     #  CZ   - O
  20  25   2    0.500    0.068   -0.464    0.3255     0.50728     #  CZ   - O
  21  23   2    0.500   -0.384    0.188    0.3410     0.55900     #  OH   - C
  21  24   2    0.500   -0.384   -0.450    0.3015     0.79054     #  OH   - O
  21  25   2    0.500   -0.384   -0.464    0.3015     0.79054     #  OH   - O
  22  23   2    0.500    0.362    0.188    0.1875     0.00000     #  HH   - C
  22  24   2    0.500    0.362   -0.450    0.1480     0.00000     #  HH   - O
  22  25   2    0.500    0.362   -0.464    0.1480     0.00000     #  HH   - O

; Exclusions from default nonbonded
[ exclusions ]
; ai       aj
    1     2
    1     3
    1     4
    1     5
    1     6
    1     7
    1     8
    1     9
    1    10
    1    11
    1    12
    1    13
    1    14
    1    15
    1    16
    1    17
    1    18
    1    19
    1    20
    1    21
    1    22
    1    23
    1    24
    1    25
    2     3
    2     4
    2     5
    2     6
    2     7
    2     8
    2     9
    2    10
    2    11
    2    12
    2    13
    2    14
    2    15
    2    16
    2    17
    2    18
    2    19
    2    20
    2    21
    2    22
    2    23
    2    24
    2    25
```

| | |
|---|---|
| 3 | 4 |
| 3 | 5 |
| 3 | 6 |
| 3 | 7 |
| 3 | 8 |
| 3 | 9 |
| 3 | 10 |
| 3 | 11 |
| 3 | 12 |
| 3 | 13 |
| 3 | 14 |
| 3 | 15 |
| 3 | 16 |
| 3 | 17 |
| 3 | 18 |
| 3 | 19 |
| 3 | 20 |
| 3 | 21 |
| 3 | 22 |
| 3 | 23 |
| 3 | 24 |
| 3 | 25 |
| 4 | 5 |
| 4 | 6 |
| 4 | 7 |
| 4 | 8 |
| 4 | 9 |
| 4 | 10 |
| 4 | 11 |
| 4 | 12 |
| 4 | 13 |
| 4 | 14 |
| 4 | 15 |
| 4 | 16 |
| 4 | 17 |
| 4 | 18 |
| 4 | 19 |
| 4 | 20 |
| 4 | 21 |
| 4 | 22 |
| 4 | 23 |
| 4 | 24 |
| 4 | 25 |
| 5 | 6 |
| 5 | 7 |
| 5 | 8 |
| 5 | 9 |
| 5 | 10 |
| 5 | 11 |
| 5 | 12 |
| 5 | 13 |
| 5 | 14 |
| 5 | 15 |
| 5 | 16 |
| 5 | 17 |
| 5 | 18 |
| 5 | 19 |
| 5 | 20 |
| 5 | 21 |
| 5 | 22 |

| | |
|---|---|
| 5 | 23 |
| 5 | 24 |
| 5 | 25 |
| 6 | 7 |
| 6 | 8 |
| 6 | 9 |
| 6 | 10 |
| 6 | 11 |
| 6 | 12 |
| 6 | 13 |
| 6 | 14 |
| 6 | 15 |
| 6 | 16 |
| 6 | 17 |
| 6 | 18 |
| 6 | 19 |
| 6 | 20 |
| 6 | 21 |
| 6 | 22 |
| 6 | 23 |
| 6 | 24 |
| 6 | 25 |
| 7 | 8 |
| 7 | 9 |
| 7 | 10 |
| 7 | 11 |
| 7 | 12 |
| 7 | 13 |
| 7 | 14 |
| 7 | 15 |
| 7 | 16 |
| 7 | 17 |
| 7 | 18 |
| 7 | 19 |
| 7 | 20 |
| 7 | 21 |
| 7 | 22 |
| 7 | 23 |
| 7 | 24 |
| 7 | 25 |
| 8 | 9 |
| 8 | 10 |
| 8 | 11 |
| 8 | 12 |
| 8 | 13 |
| 8 | 14 |
| 8 | 15 |
| 8 | 16 |
| 8 | 17 |
| 8 | 18 |
| 8 | 19 |
| 8 | 20 |
| 8 | 21 |
| 8 | 22 |
| 8 | 23 |
| 8 | 24 |
| 8 | 25 |
| 9 | 10 |
| 9 | 11 |
| 9 | 12 |

| | |
|---|---|
| 9 | 13 |
| 9 | 14 |
| 9 | 15 |
| 9 | 16 |
| 9 | 17 |
| 9 | 18 |
| 9 | 19 |
| 9 | 20 |
| 9 | 21 |
| 9 | 22 |
| 9 | 23 |
| 9 | 24 |
| 9 | 25 |
| 10 | 11 |
| 10 | 12 |
| 10 | 13 |
| 10 | 14 |
| 10 | 15 |
| 10 | 16 |
| 10 | 17 |
| 10 | 18 |
| 10 | 19 |
| 10 | 20 |
| 10 | 21 |
| 10 | 22 |
| 10 | 23 |
| 10 | 24 |
| 10 | 25 |
| 11 | 12 |
| 11 | 13 |
| 11 | 14 |
| 11 | 15 |
| 11 | 16 |
| 11 | 17 |
| 11 | 18 |
| 11 | 19 |
| 11 | 20 |
| 11 | 21 |
| 11 | 22 |
| 11 | 23 |
| 11 | 24 |
| 11 | 25 |
| 12 | 13 |
| 12 | 14 |
| 12 | 15 |
| 12 | 16 |
| 12 | 17 |
| 12 | 18 |
| 12 | 19 |
| 12 | 20 |
| 12 | 21 |
| 12 | 22 |
| 12 | 23 |
| 12 | 24 |
| 12 | 25 |
| 13 | 14 |
| 13 | 15 |
| 13 | 16 |
| 13 | 17 |
| 13 | 18 |

| | |
|---|---|
| 13 | 19 |
| 13 | 20 |
| 13 | 21 |
| 13 | 22 |
| 13 | 23 |
| 13 | 24 |
| 13 | 25 |
| 14 | 15 |
| 14 | 16 |
| 14 | 17 |
| 14 | 18 |
| 14 | 19 |
| 14 | 20 |
| 14 | 21 |
| 14 | 22 |
| 14 | 23 |
| 14 | 24 |
| 14 | 25 |
| 15 | 16 |
| 15 | 17 |
| 15 | 18 |
| 15 | 19 |
| 15 | 20 |
| 15 | 21 |
| 15 | 22 |
| 15 | 23 |
| 15 | 24 |
| 15 | 25 |
| 16 | 17 |
| 16 | 18 |
| 16 | 19 |
| 16 | 20 |
| 16 | 21 |
| 16 | 22 |
| 16 | 23 |
| 16 | 24 |
| 16 | 25 |
| 17 | 18 |
| 17 | 19 |
| 17 | 20 |
| 17 | 21 |
| 17 | 22 |
| 17 | 23 |
| 17 | 24 |
| 17 | 25 |
| 18 | 19 |
| 18 | 20 |
| 18 | 21 |
| 18 | 22 |
| 18 | 23 |
| 18 | 24 |
| 18 | 25 |
| 19 | 20 |
| 19 | 21 |
| 19 | 22 |
| 19 | 23 |
| 19 | 24 |
| 19 | 25 |
| 20 | 21 |
| 20 | 22 |

| | |
|---|---|
| 20 | 23 |
| 20 | 24 |
| 20 | 25 |
| 21 | 22 |
| 21 | 23 |
| 21 | 24 |
| 21 | 25 |
| 22 | 23 |
| 22 | 24 |
| 22 | 25 |
| 23 | 24 |
| 23 | 25 |
| 24 | 25 |

**droxidopa.itp**

```
; OPLS AA force field parameters of Droxidopa generated with JOYCE

[ moleculetype ]
; Name            nrexcl
DRO     3

[ atoms ]
;   nr       type  resnr residue  atom   cgnr     charge       mass  typeB    chargeB
; residue   1 DRO rtp DRO  q +0.0
     1   oplsm_287      1    DRO      N      1   -0.563874    14.0027
     2   oplsm_290      1    DRO     H1      1    0.334884      1.008
     3   oplsm_290      1    DRO     H2      1    0.383193      1.008
     4   oplsm_290      1    DRO     H3      1    0.347978      1.008
     5   oplsm_293      1    DRO     CA      1    0.025501     12.011
     6   oplsm_140      1    DRO     HA      1    0.138072      1.008
     7   oplsm_149      1    DRO     CB      2    0.018493     12.011
     8   oplsm_140      1    DRO    HB1      2    0.112580      1.008
     9   oplsm_154      1    DRO     OG      3   -0.469561    15.9994
    10   oplsm_155      1    DRO     HG      3    0.286741      1.008
    11   oplsm_145      1    DRO     CG      3   -0.037767     12.011
    12   oplsm_145      1    DRO    CD1      4   -0.106979     12.011
    13   oplsm_146      1    DRO    HD1      4    0.113467      1.008
    14   oplsm_145      1    DRO    CD2      5   -0.106878     12.011
    15   oplsm_146      1    DRO    HD2      5    0.096655      1.008
    16   oplsm_166      1    DRO    CE1      6    0.071455     12.011
    17   oplsm_167      1    DRO    OE1      6   -0.382444    15.9994
    18   oplsm_168      1    DRO    HE1      6    0.363284      1.008
    19   oplsm_145      1    DRO    CE2      7   -0.103248     12.011
    20   oplsm_146      1    DRO    HE2      7    0.108686      1.008
    21   oplsm_166      1    DRO     CZ      8    0.073032     12.011
    22   oplsm_167      1    DRO     OH      8   -0.393531    15.9994
    23   oplsm_168      1    DRO     HH      8    0.341308      1.008
    24   oplsm_271      1    DRO      C      9    0.201167     12.011
    25   oplsm_272      1    DRO     O1      9   -0.421581    15.9994
    26   oplsm_272      1    DRO     O2      9   -0.430621    15.9994   ; qtot 0.000013

; Stretchings
[ bonds ]
     1    2   1     0.1048     373470.846
     1    3   1     0.1021     373470.846
     1    4   1     0.1027     373470.846
     1    5   1     0.1499     207081.212
     5    6   1     0.1096     317299.775
     5    7   1     0.1551     154162.502
     7    8   1     0.1099     305252.010
     7    9   1     0.1420     225968.096
     9   10   1     0.0987     383776.997
     7   11   1     0.1520     208485.422
    11   12   1     0.1404     316021.400
    12   13   1     0.1093     332268.673
    11   14   1     0.1401     316021.400
    14   15   1     0.1089     332268.673
    12   16   1     0.1395     316021.400
    16   17   1     0.1372     289583.572
    17   18   1     0.0966     481033.117
    14   19   1     0.1396     316021.400
    19   20   1     0.1090     332268.673
    16   21   1     0.1404     321359.557
```

| | | | | |
|---|---|---|---|---|
| 19 | 21 | 1 | 0.1395 | 321359.557 |
| 21 | 22 | 1 | 0.1367 | 309471.243 |
| 22 | 23 | 1 | 0.0969 | 481033.117 |
| 5 | 24 | 1 | 0.1558 | 132339.541 |
| 24 | 25 | 1 | 0.1259 | 479501.584 |
| 24 | 26 | 1 | 0.1257 | 479501.584 |

; Bendings
[ angles ]

| | | | | | |
|---|---|---|---|---|---|
| 2 | 1 | 3 | 1 | 111.49 | 343.4745 |
| 2 | 1 | 4 | 1 | 108.24 | 343.4745 |
| 2 | 1 | 5 | 1 | 102.59 | 361.7045 |
| 3 | 1 | 4 | 1 | 108.69 | 343.4745 |
| 3 | 1 | 5 | 1 | 114.05 | 361.7045 |
| 4 | 1 | 5 | 1 | 111.56 | 361.7045 |
| 1 | 5 | 6 | 1 | 107.66 | 519.9423 |
| 1 | 5 | 7 | 1 | 111.87 | 553.7967 |
| 1 | 5 | 24 | 1 | 105.89 | 484.0496 |
| 6 | 5 | 7 | 1 | 108.85 | 417.8032 |
| 6 | 5 | 24 | 1 | 107.90 | 291.6417 |
| 5 | 7 | 8 | 1 | 107.60 | 419.7621 |
| 5 | 7 | 9 | 1 | 108.48 | 744.6744 |
| 5 | 7 | 11 | 1 | 111.66 | 365.0700 |
| 7 | 5 | 24 | 1 | 114.39 | 468.9387 |
| 8 | 7 | 9 | 1 | 106.95 | 492.3242 |
| 8 | 7 | 11 | 1 | 107.98 | 276.5572 |
| 7 | 9 | 10 | 1 | 105.60 | 632.6532 |
| 9 | 7 | 11 | 1 | 113.89 | 449.8951 |
| 7 | 11 | 12 | 1 | 119.34 | 687.2295 |
| 7 | 11 | 14 | 1 | 121.60 | 687.2295 |
| 11 | 12 | 13 | 1 | 120.26 | 298.4541 |
| 12 | 11 | 14 | 1 | 119.01 | 496.0446 |
| 11 | 12 | 16 | 1 | 120.54 | 496.0446 |
| 13 | 12 | 16 | 1 | 119.19 | 298.4541 |
| 11 | 14 | 15 | 1 | 120.01 | 298.4541 |
| 11 | 14 | 19 | 1 | 120.51 | 496.0446 |
| 15 | 14 | 19 | 1 | 119.48 | 298.4541 |
| 12 | 16 | 17 | 1 | 124.40 | 731.9646 |
| 12 | 16 | 21 | 1 | 120.09 | 377.2683 |
| 16 | 17 | 18 | 1 | 110.69 | 453.9904 |
| 17 | 16 | 21 | 1 | 115.50 | 634.4644 |
| 14 | 19 | 20 | 1 | 120.87 | 298.4541 |
| 14 | 19 | 21 | 1 | 120.33 | 377.2683 |
| 20 | 19 | 21 | 1 | 118.80 | 323.2446 |
| 16 | 21 | 19 | 1 | 119.52 | 209.4400 |
| 16 | 21 | 22 | 1 | 120.77 | 671.0560 |
| 19 | 21 | 22 | 1 | 119.71 | 671.0560 |
| 21 | 22 | 23 | 1 | 108.62 | 481.9262 |
| 5 | 24 | 25 | 1 | 116.12 | 324.2452 |
| 5 | 24 | 26 | 1 | 115.50 | 324.2452 |
| 25 | 24 | 26 | 1 | 128.37 | 813.2475 |

; Torsions
[ dihedrals ]

| | | | | | | | |
|---|---|---|---|---|---|---|---|
| 6 | 5 | 24 | 25 | 1 | 328.39 | 10.451 | 2 |
| 6 | 5 | 24 | 26 | 1 | 330.59 | 10.451 | 2 |
| 2 | 1 | 5 | 6 | 1 | 125.59 | 2.547 | 3 |
| 3 | 1 | 5 | 6 | 1 | 123.45 | 2.547 | 3 |
| 19 | 21 | 22 | 23 | 1 | 359.86 | 3.272 | 1 |
| 19 | 21 | 22 | 23 | 1 | 179.73 | 8.853 | 2 |

```
    6   5   7   8   1   -218.42       3.367   1
    6   5   7   8   1    -87.74       9.305   2
    6   5   7   8   1    -25.06       9.134   3
    6   5   7   8   1   -144.67       0.009   4
    8   7  11  14   1   -120.90       0.702   1
    8   7  11  14   1    185.55      10.437   2
    8   7  11  14   1     41.83       0.182   3
    8   7  11  14   1      0.00       0.042   4
    8   7  11  12   1   -120.90       0.702   1
    8   7  11  12   1    185.55      10.437   2
    8   7  11  12   1     41.83       0.182   3
    8   7  11  12   1      0.00       0.042   4
   12  16  17  18   1      0.00       3.272   1
   12  16  17  18   1    179.99       8.853   2
    5   7   9  10   1   -215.05      12.202   1
    5   7   9  10   1    128.96       6.720   2
    5   7   9  10   1    777.43       2.465   3
    5   7   9  10   1     43.77       0.432   4

; Nonbonded terms
[ pairs ]
; 1-4 interactions
    1   8   2   0.500   -0.564   0.113   0.2875   0.14940   #  N    - HA
    1   9   2   0.500   -0.564  -0.470   0.3160   0.35564   #  N    - OH
    1  11   2   0.500   -0.564  -0.038   0.3400   0.22821   #  N    - CC
    1  25   2   0.500   -0.564  -0.422   0.3105   0.39527   #  N    - O
    1  26   2   0.500   -0.564  -0.431   0.3105   0.39527   #  N    - O
    2   6   2   0.500    0.335   0.138   0.1250   0.00000   #  H    - HA
    2   7   2   0.500    0.335   0.018   0.1750   0.00000   #  H    - CB
    2  24   2   0.500    0.335   0.201   0.1875   0.00000   #  H    - C
    3   6   2   0.500    0.383   0.138   0.1250   0.00000   #  H    - HA
    3   7   2   0.500    0.383   0.018   0.1750   0.00000   #  H    - CB
    3  24   2   0.500    0.383   0.201   0.1875   0.00000   #  H    - C
    4   6   2   0.500    0.348   0.138   0.1250   0.00000   #  H    - HA
    4   7   2   0.500    0.348   0.018   0.1750   0.00000   #  H    - CB
    4  24   2   0.500    0.348   0.201   0.1875   0.00000   #  H    - C
    5  10   2   0.500    0.026   0.287   0.1750   0.00000   #  CA   - HO
    5  12   2   0.500    0.026  -0.107   0.3525   0.14219   #  CA   - CC
    5  14   2   0.500    0.026  -0.107   0.3525   0.14219   #  CA   - CC
    6   8   2   0.500    0.138   0.113   0.2500   0.06276   #  HA   - HA
    6   9   2   0.500    0.138  -0.470   0.2785   0.14940   #  HA   - OH
    6  11   2   0.500    0.138  -0.038   0.3025   0.09587   #  HA   - CC
    6  25   2   0.500    0.138  -0.422   0.2730   0.16605   #  HA   - O
    6  26   2   0.500    0.138  -0.431   0.2730   0.16605   #  HA   - O
    7  13   2   0.500    0.018   0.113   0.2960   0.09309   #  CB   - HC
    7  15   2   0.500    0.018   0.097   0.2960   0.09309   #  CB   - HC
    7  16   2   0.500    0.018   0.071   0.3525   0.14219   #  CB   - CC
    7  19   2   0.500    0.018  -0.103   0.3525   0.14219   #  CB   - CC
    7  25   2   0.500    0.018  -0.422   0.3230   0.24629   #  CB   - O
    7  26   2   0.500    0.018  -0.431   0.3230   0.24629   #  CB   - O
    8  10   2   0.500    0.113   0.287   0.1250   0.00000   #  HA   - HO
    8  12   2   0.500    0.113  -0.107   0.3025   0.09587   #  HA   - CC
    8  14   2   0.500    0.113  -0.107   0.3025   0.09587   #  HA   - CC
    8  24   2   0.500    0.113   0.201   0.3125   0.11741   #  HA   - C
    9  12   2   0.500   -0.470  -0.107   0.3310   0.22821   #  OH   - CC
    9  14   2   0.500   -0.470  -0.107   0.3310   0.22821   #  OH   - CC
    9  24   2   0.500   -0.470   0.201   0.3410   0.27950   #  OH   - C
   10  11   2   0.500    0.287  -0.038   0.1775   0.00000   #  HO   - CC
   11  17   2   0.500   -0.038  -0.382   0.3310   0.22821   #  CC   - OH
   11  20   2   0.500   -0.038   0.109   0.2985   0.09587   #  CC   - HC
```

| | | | | | | | | |
|---|---|---|---|---|---|---|---|---|
| 11 | 21 | 2 | 0.500 | -0.038 | 0.073 | 0.3550 | 0.14644 | # CC - CZ |
| 11 | 24 | 2 | 0.500 | -0.038 | 0.201 | 0.3650 | 0.17935 | # CC - C |
| 12 | 15 | 2 | 0.500 | -0.107 | 0.097 | 0.2985 | 0.09587 | # CC - HC |
| 12 | 18 | 2 | 0.500 | -0.107 | 0.363 | 0.1775 | 0.00000 | # CC - HH |
| 12 | 19 | 2 | 0.500 | -0.107 | -0.103 | 0.3550 | 0.14644 | # CC - CC |
| 12 | 22 | 2 | 0.500 | -0.107 | -0.394 | 0.3310 | 0.22821 | # CC - OH |
| 13 | 14 | 2 | 0.500 | 0.113 | -0.107 | 0.2985 | 0.09587 | # HC - CC |
| 13 | 17 | 2 | 0.500 | 0.113 | -0.382 | 0.2745 | 0.14940 | # HC - OH |
| 13 | 21 | 2 | 0.500 | 0.113 | 0.073 | 0.2985 | 0.09587 | # HC - CZ |
| 14 | 16 | 2 | 0.500 | -0.107 | 0.071 | 0.3550 | 0.14644 | # CC - CC |
| 14 | 22 | 2 | 0.500 | -0.107 | -0.394 | 0.3310 | 0.22821 | # CC - OH |
| 15 | 20 | 2 | 0.500 | 0.097 | 0.109 | 0.2420 | 0.06276 | # HC - HC |
| 15 | 21 | 2 | 0.500 | 0.097 | 0.073 | 0.2985 | 0.09587 | # HC - CZ |
| 16 | 20 | 2 | 0.500 | 0.071 | 0.109 | 0.2985 | 0.09587 | # CC - HC |
| 16 | 23 | 2 | 0.500 | 0.071 | 0.341 | 0.1775 | 0.00000 | # CC - HH |
| 17 | 19 | 2 | 0.500 | -0.382 | -0.103 | 0.3310 | 0.22821 | # OH - CC |
| 17 | 22 | 2 | 0.500 | -0.382 | -0.394 | 0.3070 | 0.35564 | # OH - OH |
| 18 | 21 | 2 | 0.500 | 0.363 | 0.073 | 0.1775 | 0.00000 | # HH - CZ |
| 19 | 23 | 2 | 0.500 | -0.103 | 0.341 | 0.1775 | 0.00000 | # CC - HH |
| 20 | 22 | 2 | 0.500 | 0.109 | -0.394 | 0.2745 | 0.14940 | # HC - OH |
| 1 | 10 | 2 | 0.500 | -0.564 | 0.287 | 0.1625 | 0.00000 | # N - HO |
| 1 | 12 | 2 | 0.500 | -0.564 | -0.107 | 0.3400 | 0.45642 | # N - CC |
| 1 | 13 | 2 | 0.500 | -0.564 | 0.113 | 0.2835 | 0.29880 | # N - HC |
| 1 | 14 | 2 | 0.500 | -0.564 | -0.107 | 0.3400 | 0.45642 | # N - CC |
| 1 | 15 | 2 | 0.500 | -0.564 | 0.097 | 0.2835 | 0.29880 | # N - HC |
| 1 | 16 | 2 | 0.500 | -0.564 | 0.071 | 0.3400 | 0.45642 | # N - CC |
| 1 | 17 | 2 | 0.500 | -0.564 | -0.382 | 0.3160 | 0.71128 | # N - OH |
| 1 | 18 | 2 | 0.500 | -0.564 | 0.363 | 0.1625 | 0.00000 | # N - HH |
| 1 | 19 | 2 | 0.500 | -0.564 | -0.103 | 0.3400 | 0.45642 | # N - CC |
| 1 | 20 | 2 | 0.500 | -0.564 | 0.109 | 0.2835 | 0.29880 | # N - HC |
| 1 | 21 | 2 | 0.500 | -0.564 | 0.073 | 0.3400 | 0.45642 | # N - CZ |
| 1 | 22 | 2 | 0.500 | -0.564 | -0.394 | 0.3160 | 0.71128 | # N - OH |
| 1 | 23 | 2 | 0.500 | -0.564 | 0.341 | 0.1625 | 0.00000 | # N - HH |
| 2 | 8 | 2 | 0.500 | 0.335 | 0.113 | 0.1250 | 0.00000 | # H - HA |
| 2 | 9 | 2 | 0.500 | 0.335 | -0.470 | 0.1535 | 0.00000 | # H - OH |
| 2 | 10 | 2 | 0.500 | 0.335 | 0.287 | 0.0000 | 0.00000 | # H - HO |
| 2 | 11 | 2 | 0.500 | 0.335 | -0.038 | 0.1775 | 0.00000 | # H - CC |
| 2 | 12 | 2 | 0.500 | 0.335 | -0.107 | 0.1775 | 0.00000 | # H - CC |
| 2 | 13 | 2 | 0.500 | 0.335 | 0.113 | 0.1210 | 0.00000 | # H - HC |
| 2 | 14 | 2 | 0.500 | 0.335 | -0.107 | 0.1775 | 0.00000 | # H - CC |
| 2 | 15 | 2 | 0.500 | 0.335 | 0.097 | 0.1210 | 0.00000 | # H - HC |
| 2 | 16 | 2 | 0.500 | 0.335 | 0.071 | 0.1775 | 0.00000 | # H - CC |
| 2 | 17 | 2 | 0.500 | 0.335 | -0.382 | 0.1535 | 0.00000 | # H - OH |
| 2 | 18 | 2 | 0.500 | 0.335 | 0.363 | 0.0000 | 0.00000 | # H - HH |
| 2 | 19 | 2 | 0.500 | 0.335 | -0.103 | 0.1775 | 0.00000 | # H - CC |
| 2 | 20 | 2 | 0.500 | 0.335 | 0.109 | 0.1210 | 0.00000 | # H - HC |
| 2 | 21 | 2 | 0.500 | 0.335 | 0.073 | 0.1775 | 0.00000 | # H - CZ |
| 2 | 22 | 2 | 0.500 | 0.335 | -0.394 | 0.1535 | 0.00000 | # H - OH |
| 2 | 23 | 2 | 0.500 | 0.335 | 0.341 | 0.0000 | 0.00000 | # H - HH |
| 2 | 25 | 2 | 0.500 | 0.335 | -0.422 | 0.1480 | 0.00000 | # H - O |
| 2 | 26 | 2 | 0.500 | 0.335 | -0.431 | 0.1480 | 0.00000 | # H - O |
| 3 | 8 | 2 | 0.500 | 0.383 | 0.113 | 0.1250 | 0.00000 | # H - HA |
| 3 | 9 | 2 | 0.500 | 0.383 | -0.470 | 0.1535 | 0.00000 | # H - OH |
| 3 | 10 | 2 | 0.500 | 0.383 | 0.287 | 0.0000 | 0.00000 | # H - HO |
| 3 | 11 | 2 | 0.500 | 0.383 | -0.038 | 0.1775 | 0.00000 | # H - CC |
| 3 | 12 | 2 | 0.500 | 0.383 | -0.107 | 0.1775 | 0.00000 | # H - CC |
| 3 | 13 | 2 | 0.500 | 0.383 | 0.113 | 0.1210 | 0.00000 | # H - HC |
| 3 | 14 | 2 | 0.500 | 0.383 | -0.107 | 0.1775 | 0.00000 | # H - CC |
| 3 | 15 | 2 | 0.500 | 0.383 | 0.097 | 0.1210 | 0.00000 | # H - HC |
| 3 | 16 | 2 | 0.500 | 0.383 | 0.071 | 0.1775 | 0.00000 | # H - CC |

| | | | | | | | | |
|---|---|---|---|---|---|---|---|---|
| 3 | 17 | 2 | 0.500 | 0.383 | -0.382 | 0.1535 | 0.00000 | # H - OH |
| 3 | 18 | 2 | 0.500 | 0.383 | 0.363 | 0.0000 | 0.00000 | # H - HH |
| 3 | 19 | 2 | 0.500 | 0.383 | -0.103 | 0.1775 | 0.00000 | # H - CC |
| 3 | 20 | 2 | 0.500 | 0.383 | 0.109 | 0.1210 | 0.00000 | # H - HC |
| 3 | 21 | 2 | 0.500 | 0.383 | 0.073 | 0.1775 | 0.00000 | # H - CZ |
| 3 | 22 | 2 | 0.500 | 0.383 | -0.394 | 0.1535 | 0.00000 | # H - OH |
| 3 | 23 | 2 | 0.500 | 0.383 | 0.341 | 0.0000 | 0.00000 | # H - HH |
| 3 | 25 | 2 | 0.500 | 0.383 | -0.422 | 0.1480 | 0.00000 | # H - O |
| 3 | 26 | 2 | 0.500 | 0.383 | -0.431 | 0.1480 | 0.00000 | # H - O |
| 4 | 8 | 2 | 0.500 | 0.348 | 0.113 | 0.1250 | 0.00000 | # H - HA |
| 4 | 9 | 2 | 0.500 | 0.348 | -0.470 | 0.1535 | 0.00000 | # H - OH |
| 4 | 10 | 2 | 0.500 | 0.348 | 0.287 | 0.0000 | 0.00000 | # H - HO |
| 4 | 11 | 2 | 0.500 | 0.348 | -0.038 | 0.1775 | 0.00000 | # H - CC |
| 4 | 12 | 2 | 0.500 | 0.348 | -0.107 | 0.1775 | 0.00000 | # H - CC |
| 4 | 13 | 2 | 0.500 | 0.348 | 0.113 | 0.1210 | 0.00000 | # H - HC |
| 4 | 14 | 2 | 0.500 | 0.348 | -0.107 | 0.1775 | 0.00000 | # H - CC |
| 4 | 15 | 2 | 0.500 | 0.348 | 0.097 | 0.1210 | 0.00000 | # H - HC |
| 4 | 16 | 2 | 0.500 | 0.348 | 0.071 | 0.1775 | 0.00000 | # H - CC |
| 4 | 17 | 2 | 0.500 | 0.348 | -0.382 | 0.1535 | 0.00000 | # H - OH |
| 4 | 18 | 2 | 0.500 | 0.348 | 0.363 | 0.0000 | 0.00000 | # H - HH |
| 4 | 19 | 2 | 0.500 | 0.348 | -0.103 | 0.1775 | 0.00000 | # H - CC |
| 4 | 20 | 2 | 0.500 | 0.348 | 0.109 | 0.1210 | 0.00000 | # H - HC |
| 4 | 21 | 2 | 0.500 | 0.348 | 0.073 | 0.1775 | 0.00000 | # H - CZ |
| 4 | 22 | 2 | 0.500 | 0.348 | -0.394 | 0.1535 | 0.00000 | # H - OH |
| 4 | 23 | 2 | 0.500 | 0.348 | 0.341 | 0.0000 | 0.00000 | # H - HH |
| 4 | 25 | 2 | 0.500 | 0.348 | -0.422 | 0.1480 | 0.00000 | # H - O |
| 4 | 26 | 2 | 0.500 | 0.348 | -0.431 | 0.1480 | 0.00000 | # H - O |
| 5 | 13 | 2 | 0.500 | 0.026 | 0.113 | 0.2960 | 0.18618 | # CA - HC |
| 5 | 15 | 2 | 0.500 | 0.026 | 0.097 | 0.2960 | 0.18618 | # CA - HC |
| 5 | 16 | 2 | 0.500 | 0.026 | 0.071 | 0.3525 | 0.28439 | # CA - CC |
| 5 | 17 | 2 | 0.500 | 0.026 | -0.382 | 0.3285 | 0.44319 | # CA - OH |
| 5 | 18 | 2 | 0.500 | 0.026 | 0.363 | 0.1750 | 0.00000 | # CA - HH |
| 5 | 19 | 2 | 0.500 | 0.026 | -0.103 | 0.3525 | 0.28439 | # CA - CC |
| 5 | 20 | 2 | 0.500 | 0.026 | 0.109 | 0.2960 | 0.18618 | # CA - HC |
| 5 | 21 | 2 | 0.500 | 0.026 | 0.073 | 0.3525 | 0.28439 | # CA - CZ |
| 5 | 22 | 2 | 0.500 | 0.026 | -0.394 | 0.3285 | 0.44319 | # CA - OH |
| 5 | 23 | 2 | 0.500 | 0.026 | 0.341 | 0.1750 | 0.00000 | # CA - HH |
| 6 | 10 | 2 | 0.500 | 0.138 | 0.287 | 0.1250 | 0.00000 | # HA - HO |
| 6 | 12 | 2 | 0.500 | 0.138 | -0.107 | 0.3025 | 0.19173 | # HA - CC |
| 6 | 13 | 2 | 0.500 | 0.138 | 0.113 | 0.2460 | 0.12552 | # HA - HC |
| 6 | 14 | 2 | 0.500 | 0.138 | -0.107 | 0.3025 | 0.19173 | # HA - CC |
| 6 | 15 | 2 | 0.500 | 0.138 | 0.097 | 0.2460 | 0.12552 | # HA - HC |
| 6 | 16 | 2 | 0.500 | 0.138 | 0.071 | 0.3025 | 0.19173 | # HA - CC |
| 6 | 17 | 2 | 0.500 | 0.138 | -0.382 | 0.2785 | 0.29880 | # HA - OH |
| 6 | 18 | 2 | 0.500 | 0.138 | 0.363 | 0.1250 | 0.00000 | # HA - HH |
| 6 | 19 | 2 | 0.500 | 0.138 | -0.103 | 0.3025 | 0.19173 | # HA - CC |
| 6 | 20 | 2 | 0.500 | 0.138 | 0.109 | 0.2460 | 0.12552 | # HA - HC |
| 6 | 21 | 2 | 0.500 | 0.138 | 0.073 | 0.3025 | 0.19173 | # HA - CZ |
| 6 | 22 | 2 | 0.500 | 0.138 | -0.394 | 0.2785 | 0.29880 | # HA - OH |
| 6 | 23 | 2 | 0.500 | 0.138 | 0.341 | 0.1250 | 0.00000 | # HA - HH |
| 7 | 17 | 2 | 0.500 | 0.018 | -0.382 | 0.3285 | 0.44319 | # CB - OH |
| 7 | 18 | 2 | 0.500 | 0.018 | 0.363 | 0.1750 | 0.00000 | # CB - HH |
| 7 | 20 | 2 | 0.500 | 0.018 | 0.109 | 0.2960 | 0.18618 | # CB - HC |
| 7 | 21 | 2 | 0.500 | 0.018 | 0.073 | 0.3525 | 0.28439 | # CB - CZ |
| 7 | 22 | 2 | 0.500 | 0.018 | -0.394 | 0.3285 | 0.44319 | # CB - OH |
| 7 | 23 | 2 | 0.500 | 0.018 | 0.341 | 0.1750 | 0.00000 | # CB - HH |
| 8 | 13 | 2 | 0.500 | 0.113 | 0.113 | 0.2460 | 0.12552 | # HA - HC |
| 8 | 15 | 2 | 0.500 | 0.113 | 0.097 | 0.2460 | 0.12552 | # HA - HC |
| 8 | 16 | 2 | 0.500 | 0.113 | 0.071 | 0.3025 | 0.19173 | # HA - CC |
| 8 | 17 | 2 | 0.500 | 0.113 | -0.382 | 0.2785 | 0.29880 | # HA - OH |

```
8  18   2    0.500    0.113    0.363    0.1250    0.00000    #  HA   - HH
8  19   2    0.500    0.113   -0.103    0.3025    0.19173    #  HA   - CC
8  20   2    0.500    0.113    0.109    0.2460    0.12552    #  HA   - HC
8  21   2    0.500    0.113    0.073    0.3025    0.19173    #  HA   - CZ
8  22   2    0.500    0.113   -0.394    0.2785    0.29880    #  HA   - OH
8  23   2    0.500    0.113    0.341    0.1250    0.00000    #  HA   - HH
8  25   2    0.500    0.113   -0.422    0.2730    0.33209    #  HA   - O
8  26   2    0.500    0.113   -0.431    0.2730    0.33209    #  HA   - O
9  13   2    0.500   -0.470    0.113    0.2745    0.29880    #  OH   - HC
9  15   2    0.500   -0.470    0.097    0.2745    0.29880    #  OH   - HC
9  16   2    0.500   -0.470    0.071    0.3310    0.45642    #  OH   - CC
9  17   2    0.500   -0.470   -0.382    0.3070    0.71128    #  OH   - OH
9  18   2    0.500   -0.470    0.363    0.1535    0.00000    #  OH   - HH
9  19   2    0.500   -0.470   -0.103    0.3310    0.45642    #  OH   - CC
9  20   2    0.500   -0.470    0.109    0.2745    0.29880    #  OH   - HC
9  21   2    0.500   -0.470    0.073    0.3310    0.45642    #  OH   - CZ
9  22   2    0.500   -0.470   -0.394    0.3070    0.71128    #  OH   - OH
9  23   2    0.500   -0.470    0.341    0.1535    0.00000    #  OH   - HH
9  25   2    0.500   -0.470   -0.422    0.3015    0.79054    #  OH   - O
9  26   2    0.500   -0.470   -0.431    0.3015    0.79054    #  OH   - O
10  12   2    0.500    0.287   -0.107    0.1775    0.00000    #  HO   - CC
10  13   2    0.500    0.287    0.113    0.1210    0.00000    #  HO   - HC
10  14   2    0.500    0.287   -0.107    0.1775    0.00000    #  HO   - CC
10  15   2    0.500    0.287    0.097    0.1210    0.00000    #  HO   - HC
10  16   2    0.500    0.287    0.071    0.1775    0.00000    #  HO   - CC
10  17   2    0.500    0.287   -0.382    0.1535    0.00000    #  HO   - OH
10  18   2    0.500    0.287    0.363    0.0000    0.00000    #  HO   - HH
10  19   2    0.500    0.287   -0.103    0.1775    0.00000    #  HO   - CC
10  20   2    0.500    0.287    0.109    0.1210    0.00000    #  HO   - HC
10  21   2    0.500    0.287    0.073    0.1775    0.00000    #  HO   - CZ
10  22   2    0.500    0.287   -0.394    0.1535    0.00000    #  HO   - OH
10  23   2    0.500    0.287    0.341    0.0000    0.00000    #  HO   - HH
10  24   2    0.500    0.287    0.201    0.1875    0.00000    #  HO   - C
10  25   2    0.500    0.287   -0.422    0.1480    0.00000    #  HO   - O
10  26   2    0.500    0.287   -0.431    0.1480    0.00000    #  HO   - O
11  18   2    0.500   -0.038    0.363    0.1775    0.00000    #  CC   - HH
11  22   2    0.500   -0.038   -0.394    0.3310    0.45642    #  CC   - OH
11  23   2    0.500   -0.038    0.341    0.1775    0.00000    #  CC   - HH
11  25   2    0.500   -0.038   -0.422    0.3255    0.50728    #  CC   - O
11  26   2    0.500   -0.038   -0.431    0.3255    0.50728    #  CC   - O
12  20   2    0.500   -0.107    0.109    0.2985    0.19173    #  CC   - HC
12  23   2    0.500   -0.107    0.341    0.1775    0.00000    #  CC   - HH
12  24   2    0.500   -0.107    0.201    0.3650    0.35870    #  CC   - C
12  25   2    0.500   -0.107   -0.422    0.3255    0.50728    #  CC   - O
12  26   2    0.500   -0.107   -0.431    0.3255    0.50728    #  CC   - O
13  15   2    0.500    0.113    0.097    0.2420    0.12552    #  HC   - HC
13  18   2    0.500    0.113    0.363    0.1210    0.00000    #  HC   - HH
13  19   2    0.500    0.113   -0.103    0.2985    0.19173    #  HC   - CC
13  20   2    0.500    0.113    0.109    0.2420    0.12552    #  HC   - HC
13  22   2    0.500    0.113   -0.394    0.2745    0.29880    #  HC   - OH
13  23   2    0.500    0.113    0.341    0.1210    0.00000    #  HC   - HH
13  24   2    0.500    0.113    0.201    0.3085    0.23483    #  HC   - C
13  25   2    0.500    0.113   -0.422    0.2690    0.33209    #  HC   - O
13  26   2    0.500    0.113   -0.431    0.2690    0.33209    #  HC   - O
14  17   2    0.500   -0.107   -0.382    0.3310    0.45642    #  CC   - OH
14  18   2    0.500   -0.107    0.363    0.1775    0.00000    #  CC   - HH
14  23   2    0.500   -0.107    0.341    0.1775    0.00000    #  CC   - HH
14  24   2    0.500   -0.107    0.201    0.3650    0.35870    #  CC   - C
14  25   2    0.500   -0.107   -0.422    0.3255    0.50728    #  CC   - O
14  26   2    0.500   -0.107   -0.431    0.3255    0.50728    #  CC   - O
```

```
15  16  2  0.500  0.097  0.071  0.2985  0.19173  #  HC  - CC
15  17  2  0.500  0.097  -0.382  0.2745  0.29880  #  HC  - OH
15  18  2  0.500  0.097  0.363  0.1210  0.00000  #  HC  - HH
15  22  2  0.500  0.097  -0.394  0.2745  0.29880  #  HC  - OH
15  23  2  0.500  0.097  0.341  0.1210  0.00000  #  HC  - HH
15  24  2  0.500  0.097  0.201  0.3085  0.23483  #  HC  - C
15  25  2  0.500  0.097  -0.422  0.2690  0.33209  #  HC  - O
15  26  2  0.500  0.097  -0.431  0.2690  0.33209  #  HC  - O
16  24  2  0.500  0.071  0.201  0.3650  0.35870  #  CC  - C
16  25  2  0.500  0.071  -0.422  0.3255  0.50728  #  CC  - O
16  26  2  0.500  0.071  -0.431  0.3255  0.50728  #  CC  - O
17  20  2  0.500  -0.382  0.109  0.2745  0.29880  #  OH  - HC
17  23  2  0.500  -0.382  0.341  0.1535  0.00000  #  OH  - HH
17  24  2  0.500  -0.382  0.201  0.3410  0.55900  #  OH  - C
17  25  2  0.500  -0.382  -0.422  0.3015  0.79054  #  OH  - O
17  26  2  0.500  -0.382  -0.431  0.3015  0.79054  #  OH  - O
18  19  2  0.500  0.363  -0.103  0.1775  0.00000  #  HH  - CC
18  20  2  0.500  0.363  0.109  0.1210  0.00000  #  HH  - HC
18  22  2  0.500  0.363  -0.394  0.1535  0.00000  #  HH  - OH
18  23  2  0.500  0.363  0.341  0.0000  0.00000  #  HH  - HH
18  24  2  0.500  0.363  0.201  0.1875  0.00000  #  HH  - C
18  25  2  0.500  0.363  -0.422  0.1480  0.00000  #  HH  - O
18  26  2  0.500  0.363  -0.431  0.1480  0.00000  #  HH  - O
19  24  2  0.500  -0.103  0.201  0.3650  0.35870  #  CC  - C
19  25  2  0.500  -0.103  -0.422  0.3255  0.50728  #  CC  - O
19  26  2  0.500  -0.103  -0.431  0.3255  0.50728  #  CC  - O
20  23  2  0.500  0.109  0.341  0.1210  0.00000  #  HC  - HH
20  24  2  0.500  0.109  0.201  0.3085  0.23483  #  HC  - C
20  25  2  0.500  0.109  -0.422  0.2690  0.33209  #  HC  - O
20  26  2  0.500  0.109  -0.431  0.2690  0.33209  #  HC  - O
21  24  2  0.500  0.073  0.201  0.3650  0.35870  #  CZ  - C
21  25  2  0.500  0.073  -0.422  0.3255  0.50728  #  CZ  - O
21  26  2  0.500  0.073  -0.431  0.3255  0.50728  #  CZ  - O
22  24  2  0.500  -0.394  0.201  0.3410  0.55900  #  OH  - C
22  25  2  0.500  -0.394  -0.422  0.3015  0.79054  #  OH  - O
22  26  2  0.500  -0.394  -0.431  0.3015  0.79054  #  OH  - O
23  24  2  0.500  0.341  0.201  0.1875  0.00000  #  HH  - C
23  25  2  0.500  0.341  -0.422  0.1480  0.00000  #  HH  - O
23  26  2  0.500  0.341  -0.431  0.1480  0.00000  #  HH  - O

; Exclusions from default nonbonded
[ exclusions ]
; ai    aj
  1   2
  1   3
  1   4
  1   5
  1   6
  1   7
  1   8
  1   9
  1   10
  1   11
  1   12
  1   13
  1   14
  1   15
  1   16
  1   17
  1   18
```

| | |
|---|---|
| 1 | 19 |
| 1 | 20 |
| 1 | 21 |
| 1 | 22 |
| 1 | 23 |
| 1 | 24 |
| 1 | 25 |
| 1 | 26 |
| 2 | 3 |
| 2 | 4 |
| 2 | 5 |
| 2 | 6 |
| 2 | 7 |
| 2 | 8 |
| 2 | 9 |
| 2 | 10 |
| 2 | 11 |
| 2 | 12 |
| 2 | 13 |
| 2 | 14 |
| 2 | 15 |
| 2 | 16 |
| 2 | 17 |
| 2 | 18 |
| 2 | 19 |
| 2 | 20 |
| 2 | 21 |
| 2 | 22 |
| 2 | 23 |
| 2 | 24 |
| 2 | 25 |
| 2 | 26 |
| 3 | 4 |
| 3 | 5 |
| 3 | 6 |
| 3 | 7 |
| 3 | 8 |
| 3 | 9 |
| 3 | 10 |
| 3 | 11 |
| 3 | 12 |
| 3 | 13 |
| 3 | 14 |
| 3 | 15 |
| 3 | 16 |
| 3 | 17 |
| 3 | 18 |
| 3 | 19 |
| 3 | 20 |
| 3 | 21 |
| 3 | 22 |
| 3 | 23 |
| 3 | 24 |
| 3 | 25 |
| 3 | 26 |
| 4 | 5 |
| 4 | 6 |
| 4 | 7 |
| 4 | 8 |
| 4 | 9 |

| | |
|---|---|
| 4 | 10 |
| 4 | 11 |
| 4 | 12 |
| 4 | 13 |
| 4 | 14 |
| 4 | 15 |
| 4 | 16 |
| 4 | 17 |
| 4 | 18 |
| 4 | 19 |
| 4 | 20 |
| 4 | 21 |
| 4 | 22 |
| 4 | 23 |
| 4 | 24 |
| 4 | 25 |
| 4 | 26 |
| 5 | 6 |
| 5 | 7 |
| 5 | 8 |
| 5 | 9 |
| 5 | 10 |
| 5 | 11 |
| 5 | 12 |
| 5 | 13 |
| 5 | 14 |
| 5 | 15 |
| 5 | 16 |
| 5 | 17 |
| 5 | 18 |
| 5 | 19 |
| 5 | 20 |
| 5 | 21 |
| 5 | 22 |
| 5 | 23 |
| 5 | 24 |
| 5 | 25 |
| 5 | 26 |
| 6 | 7 |
| 6 | 8 |
| 6 | 9 |
| 6 | 10 |
| 6 | 11 |
| 6 | 12 |
| 6 | 13 |
| 6 | 14 |
| 6 | 15 |
| 6 | 16 |
| 6 | 17 |
| 6 | 18 |
| 6 | 19 |
| 6 | 20 |
| 6 | 21 |
| 6 | 22 |
| 6 | 23 |
| 6 | 24 |
| 6 | 25 |
| 6 | 26 |
| 7 | 8 |
| 7 | 9 |

| | |
|---|---|
| 7 | 10 |
| 7 | 11 |
| 7 | 12 |
| 7 | 13 |
| 7 | 14 |
| 7 | 15 |
| 7 | 16 |
| 7 | 17 |
| 7 | 18 |
| 7 | 19 |
| 7 | 20 |
| 7 | 21 |
| 7 | 22 |
| 7 | 23 |
| 7 | 24 |
| 7 | 25 |
| 7 | 26 |
| 8 | 9 |
| 8 | 10 |
| 8 | 11 |
| 8 | 12 |
| 8 | 13 |
| 8 | 14 |
| 8 | 15 |
| 8 | 16 |
| 8 | 17 |
| 8 | 18 |
| 8 | 19 |
| 8 | 20 |
| 8 | 21 |
| 8 | 22 |
| 8 | 23 |
| 8 | 24 |
| 8 | 25 |
| 8 | 26 |
| 9 | 10 |
| 9 | 11 |
| 9 | 12 |
| 9 | 13 |
| 9 | 14 |
| 9 | 15 |
| 9 | 16 |
| 9 | 17 |
| 9 | 18 |
| 9 | 19 |
| 9 | 20 |
| 9 | 21 |
| 9 | 22 |
| 9 | 23 |
| 9 | 24 |
| 9 | 25 |
| 9 | 26 |
| 10 | 11 |
| 10 | 12 |
| 10 | 13 |
| 10 | 14 |
| 10 | 15 |
| 10 | 16 |
| 10 | 17 |
| 10 | 18 |

| | |
|---|---|
| 10 | 19 |
| 10 | 20 |
| 10 | 21 |
| 10 | 22 |
| 10 | 23 |
| 10 | 24 |
| 10 | 25 |
| 10 | 26 |
| 11 | 12 |
| 11 | 13 |
| 11 | 14 |
| 11 | 15 |
| 11 | 16 |
| 11 | 17 |
| 11 | 18 |
| 11 | 19 |
| 11 | 20 |
| 11 | 21 |
| 11 | 22 |
| 11 | 23 |
| 11 | 24 |
| 11 | 25 |
| 11 | 26 |
| 12 | 13 |
| 12 | 14 |
| 12 | 15 |
| 12 | 16 |
| 12 | 17 |
| 12 | 18 |
| 12 | 19 |
| 12 | 20 |
| 12 | 21 |
| 12 | 22 |
| 12 | 23 |
| 12 | 24 |
| 12 | 25 |
| 12 | 26 |
| 13 | 14 |
| 13 | 15 |
| 13 | 16 |
| 13 | 17 |
| 13 | 18 |
| 13 | 19 |
| 13 | 20 |
| 13 | 21 |
| 13 | 22 |
| 13 | 23 |
| 13 | 24 |
| 13 | 25 |
| 13 | 26 |
| 14 | 15 |
| 14 | 16 |
| 14 | 17 |
| 14 | 18 |
| 14 | 19 |
| 14 | 20 |
| 14 | 21 |
| 14 | 22 |
| 14 | 23 |
| 14 | 24 |

| | |
|---|---|
| 14 | 25 |
| 14 | 26 |
| 15 | 16 |
| 15 | 17 |
| 15 | 18 |
| 15 | 19 |
| 15 | 20 |
| 15 | 21 |
| 15 | 22 |
| 15 | 23 |
| 15 | 24 |
| 15 | 25 |
| 15 | 26 |
| 16 | 17 |
| 16 | 18 |
| 16 | 19 |
| 16 | 20 |
| 16 | 21 |
| 16 | 22 |
| 16 | 23 |
| 16 | 24 |
| 16 | 25 |
| 16 | 26 |
| 17 | 18 |
| 17 | 19 |
| 17 | 20 |
| 17 | 21 |
| 17 | 22 |
| 17 | 23 |
| 17 | 24 |
| 17 | 25 |
| 17 | 26 |
| 18 | 19 |
| 18 | 20 |
| 18 | 21 |
| 18 | 22 |
| 18 | 23 |
| 18 | 24 |
| 18 | 25 |
| 18 | 26 |
| 19 | 20 |
| 19 | 21 |
| 19 | 22 |
| 19 | 23 |
| 19 | 24 |
| 19 | 25 |
| 19 | 26 |
| 20 | 21 |
| 20 | 22 |
| 20 | 23 |
| 20 | 24 |
| 20 | 25 |
| 20 | 26 |
| 21 | 22 |
| 21 | 23 |
| 21 | 24 |
| 21 | 25 |
| 21 | 26 |
| 22 | 23 |
| 22 | 24 |

| | |
|---|---|
| 22 | 25 |
| 22 | 26 |
| 23 | 24 |
| 23 | 25 |
| 23 | 26 |
| 24 | 25 |
| 24 | 26 |
| 25 | 26 |
